# Cardiac anxiety in the perioperative period of patients undergoing cardiac surgical procedures: an observational study

**DOI:** 10.1590/0034-7167-2022-0250

**Published:** 2022-12-16

**Authors:** Bruna Sonego Kazitani, Letícia Mansano Martins, Vitor Melz da Silva, Paolla Algarte Fernandes, Suellen Rodrigues de Oliveira Maier, Carina Aparecida Marosti Dessotte

**Affiliations:** I Universidade de São Paulo. Ribeirão Preto, São Paulo, Brazil

**Keywords:** Anxiety, Thoracic Surgery, Preoperative Period, Cardiac Surgical Procedures, Nursing Care, Ansiedad, Cirugía Torácica, Periodo Perioperatorio, Procedimientos Quirúrgicos Cardíacos, Atención de Enfermería, Ansiedade, Cirurgia Torácica, Período Perioperatório, Procedimentos Cirúrgicos Cardíacos, Cuidados de Enfermagem

## Abstract

**Objective:**

to compare cardiac anxiety symptoms in patients undergoing coronary artery bypass graft and valve surgery repair in the preoperative period, on the day of hospital discharge and on the first return visit after hospital discharge.

**Methods:**

an observational study, carried out in inpatient units and in outpatient clinic of a university hospital. Data were collected through interviews. Cardiac anxiety symptoms were assessed using the Cardiac Anxiety Questionnaire.

**Results:**

we observed the effect of time on cardiac anxiety symptoms of patients undergoing coronary artery bypass graft in the total score and in the “Avoidance” domain at discharge and at the first return visit. In patients undergoing valve repair surgery, the effect of time on symptoms was observed only in the first return visit, when compared with the preoperative period.

**Conclusion:**

the findings revealed increased cardiac anxiety symptoms in the postoperative period, discharge and first return, when compared to the preoperative period.

## RESUMO

**Objetivo:**

comparar os sintomas de ansiedade cardíaca em pacientes submetidos às cirurgias de revascularização do miocárdio e de correção de valvopatias no pré-operatório, no dia da alta hospitalar e no primeiro retorno após a alta hospitalar.

**Métodos:**

estudo observacional, realizado nas unidades de internação e no ambulatório de um hospital universitário. Os dados foram coletados por entrevistas. Os sintomas de ansiedade cardíaca foram avaliados utilizando o Questionário de Ansiedade Cardíaca.

**Resultados:**

observamos o efeito do tempo nos sintomas de ansiedade cardíaca dos pacientes submetidos à cirurgia de revascularização do miocárdio no escore total e no domínio “Evitação” na alta e no primeiro retorno. Em pacientes submetidos à correção cirúrgica de valvopatias, observou-se o efeito do tempo nos sintomas apenas no primeiro retorno, quando comparados com o pré-operatório.

**Conclusão:**

os achados revelaram o aumento dos sintomas de ansiedade cardíaca no pós-operatório, alta e primeiro retorno, quando comparados com o pré-operatório.

## RESUMEN

**Objetivo:**

comparar los síntomas de ansiedad cardiaca en pacientes sometidos a cirugía de revascularización miocárdica y reparación valvular en el preoperatorio, en el día del alta hospitalaria y en la primera visita posterior al alta hospitalaria.

**Métodos:**

estudio observacional, realizado en las unidades de hospitalización y en la consulta externa de un hospital universitario. Los datos fueron recolectados a través de entrevistas. Los síntomas de ansiedad cardíaca se evaluaron mediante el Cuestionario de Ansiedad Cardíaca.

**Resultados:**

observamos el efecto del tiempo sobre los síntomas de ansiedad cardiaca de los pacientes sometidos a cirugía de revascularización miocárdica en la puntuación total y en el dominio “Evitación” al alta y en la primera visita de regreso. En pacientes sometidos a corrección quirúrgica de cardiopatía valvular, el efecto del tiempo sobre los síntomas se observó sólo en la primera visita de seguimiento, en comparación con el período preoperatorio.

**Conclusión:**

los hallazgos revelaron un aumento de los síntomas de ansiedad cardiaca en el postoperatorio, alta y primer retorno, en comparación con el preoperatorio.

## INTRODUCTION

Due to the restrictions imposed by the COVID-19 pandemic, patients with cardiovascular diseases (CVD) have sought specialized services only when the symptoms of such diseases are imminent, which may have contributed to the decrease in the number of hospitalizations of these patients from 2020. Among the various diseases that make up the CVD, coronary artery disease (CAD) and valvular heart disease (insufficiency and/or stenosis) are among the most prevalent^(1-2)^.

CVD treatment can be clinical, hemodynamic or surgical, the third being quite prevalent in the national scenario, given that, in the pre-pandemic period, in 2019, 20,674 coronary artery bypass graft (CABG) surgeries and 9,805 valve repair surgeries were performed^(2)^.

Although advances related to the treatment of such diseases have made it possible to increase the life expectancy of patients affected by CAD and valvular heart disease^(3)^, during hospitalization, they encounter adversities related to the threat to their bodily integrity by the procedures to which they were submitted, exposure of their intimacy to strangers, living in an environment of illness, pain and death, in addition to suffering due to uncertainty regarding the evolution of their clinical condition^(3-4)^.

In this context, patients awaiting cardiac surgery may experience high levels of anxiety symptoms, due to fears, concerns and uncertainties about the surgery. Studies have shown that anxiety symptoms have influenced patients’ recovery in the postoperative period (PO) of cardiac surgeries, in addition to potentiating the occurrence of complications, increasing hospital stay, decreasing the effectiveness of cardiac rehabilitation programs and increasing postoperative mortality^(5-7)^.

From this perspective, a specific type of anxiety called cardiac anxiety (CA) has caused several changes in clinical conditions during the perioperative period. It is a syndrome characterized by aversive sensations or recurrent chest pain, in the absence of physical changes that explain them^(8-9)^. Studies show that, after receiving the diagnosis of heart disease, most of the time, individuals start to focus on the functioning of the heart, being dominated by fear and concern about cardiac symptoms^(10-12)^.

Despite this, to date, few studies have been developed with the objective of investigating the presence of CA symptoms in the perioperative period of cardiac surgeries^(9,13)^, which justifies the need to search for data in the Brazilian context on CA symptoms in the preoperative period, on the day of discharge and in the outpatient follow-up. It is worth mentioning that the early identification of the presence of these symptoms, as well as the understanding of their influence on patients’ clinical condition, can enable adequate health interventions, with a view to patients’ recovery^(14)^.

## OBJECTIVE

To compare cardiac anxiety symptoms in patients undergoing coronary artery bypass graft and valve surgery repair in the preoperative period, on the day of hospital discharge and on the first return visit after hospital discharge.

## METHODS

### Ethical aspects

The present study complied with all ethical aspects concerning the development of research with human beings, as described in Resolution 466/2012 of the Brazilian National Health Council. The research project was approved by the Research Ethics Committee (REC) of the *Escola de Enfermagem de Ribeirão Preto*. Participants were invited to participate in the study voluntarily, during hospitalization, prior to the surgical procedure. Upon verbal consent, participants signed the Informed Consent Form, with one copy destined for the participant and the other remaining with the researchers.

### Study design, period, and place

This is an observational and prospective study, built according to the Strengthening the Reporting of Observational Studies in Epidemiology (STROBE), carried out in the inpatient units of a surgical clinic and in the cardiac surgery outpatient clinic of a university hospital in the countryside of São Paulo, between February 2018 and August 2019.

### Sample and inclusion and exclusion criteria

A consecutive, non-probabilistic sample consisted of adult patients, of both sexes, with a recommendation for CABG or surgery for valve repair surgery, regardless of whether it was the first surgery or reoperation, and who had elective scheduling of their surgeries more than 12 hours in advance. We excluded patients who did not have cognitive conditions to answer the questionnaires on the day of preoperative data collection or who had clinical decompensation of heart disease in the first phase of data collection. We discontinued patients who underwent a second surgical approach during hospitalization or who were readmitted before the first return visit after hospital discharge.

Participants were identified through an active search, according to elective scheduling of the surgical procedures of interest; for this reason, the consecutive and non-probabilistic convenience sample was adopted, in accordance with the objective of this study.

### Study protocol

Data collection was carried out through individual interviews and consultation of participants’ medical records in the preoperative period, in the inpatient units of the surgical clinic and on the day before surgery. Data were collected for sociodemographic and clinical characterization and assessment of CA symptoms in the PO on the day of hospital discharge, with the second assessment of CA symptoms. On the day of the first outpatient return after hospital discharge, which occurs on average 14 days after hospital discharge, CA symptoms were again assessed.

For participant sociodemographic and clinical characterization, the researchers developed an instrument based on the literature review and on previous studies, containing sociodemographic data (dates of birth, hospitalization and interview, sex, presence of a partner, education, professional status, monthly family income and number of people who depend on income, age was calculated by subtracting the date of the interview from the date of birth) and clinical data (main diagnosis, presence of associated diseases, lifestyle habits (smoking), previous use of psychotropic drugs at home, rescheduling of surgery and reason (when necessary), surgery performed, preoperative length of stay, length of stay in the Intensive Care Unit, length of stay in the ward after discharge from the Intensive Care Unit, overall length of stay, and time of first return).

To assess CA symptoms, the Cardiac Anxiety Questionnaire (CAQ)^(8)^, Brazilian version, was used^(15)^. The CAQ consists of 14 items assessed using a five-point Likert-type scale: (0) never, (1) rarely, (2) sometimes, (3) often and (4) always. This questionnaire has two domains: “Fear and hypervigilance of cardiac-related stimutli” (items 1, 3, 4, 8, 10, 11, 12, 13 and 14) and “Avoidance of activities that may trigger symptoms” (items 2, 5, 6, 7 and 9). The total score is obtained by summing the responses to the 14 items, with a possible variation from 0-56, with higher values indicating greater perception of CA by patients. It is also possible to obtain the scores of the two domains. Thus, the domain “Fear and hypervigilance of cardiac-related stimuli” (9 items) may have a range of 0-36, and the domain “Avoidance of activities that may trigger symptoms” (5 items), a range of 0 -20, both with higher values indicating greater perception of CA by patients.

### Analysis of results, and statistics

Data were entered, processed and analyzed using the IBM-SPSS program, version 22.0 for Windows (SPSS, Inc., Chicago, IL, USA). For sociodemographic and clinical characterization, descriptive analyzes of simple frequency were performed for nominal or categorical variables, and analysis of central tendency (mean and median) and dispersion (standard deviation) for numerical variables. To compare CA symptoms in patients in the preoperative period, on the day of hospital discharge and on the first return visit after hospital discharge (median), the Friedman test was used. We used “Friedman’s two-factor analysis of variance of samples related by rank” in the Friedman tests in which a statistically significant difference was found between the investigated times.

To assess the CAQ’s internal consistency, Cronbach’s Alpha Coefficient was calculated. Values above 0.70^(16)^ were considered adequate, and the significance level adopted was 0.05.

## RESULTS

During the period of recruitment of participants, 145 cardiac surgeries were performed, six patients did not meet the inclusion criteria, leaving 79 undergoing valve repair surgeries and 60 undergoing CABG.

Among the participants who underwent CABG, eight did not accept to participate in the study, 20 were considered data collection losses (did not attend the return visit to the clinic, they were not found when returning from the clinic, or were not assessed by those responsible for the research), three died and three were discontinued from the study (surgical re-approach or hospitalization soon after hospital discharge before the first outpatient return visit).

As for patients who underwent valve repair surgeries, three did not accept to participate in the study, seven were considered data collection losses, six died and five were discontinued from the study.

Thus, in the end, the sample consisted of 34 (36.9%) patients with coronary artery disease undergoing CABG and 58 (63.1%) patients with valvular heart disease undergoing valve repair surgeries, totaling 92 participants.

Table 1 shows the sociodemographic and clinical characterization of the 92 patients, 34 of whom underwent CABG and 58 underwent valve repair surgeries.

**Table 1:** Sociodemographic and clinical characterization of patients (92) undergoing coronary artery bypass graft and valve repair surgery, according to sex, age, marital status, education, monthly income, number of people who depend on income, professional status, presence of associated diseases, lifestyle habits, use of psychotropic drugs and surgery suspension, Ribeirão Preto, São Paulo, Brazil, 2018-2019

Variable	CABG^*^	CABG	CABG	Valve repair surgery	Valve repair surgery	Valve repair surgery
	n (%)	Mean (SD) ^**^	Median	n (%)	Mean (SD) ^**^	Median
Sex						
Male	22 (64.7)			30 (51.7)		
Female	12 (35.7)			28 (48.3)		
Age		62.4 (9.8)	61.5		54.7 (14.1)	57.2
Partner presence						
With a partner	23 (67.6)			41 (70.7)		
Without a partner	11 (32.4)			17 (29.3)		
Education (years)		7.2 (4.0)	6.5		6.4 (4.9)	4.5
Monthly income (reais)		2,936.2 (2,619.3)	2,390.0		2,450.93 (1,624.7)	2,000.0
Income dependents		2.7 (1.4)	2.0		2.3 (1.2)	2.0
Professional status						
Inactive	20 (58.8)			35 (60.3)		
Active	14 (41.2)			23 (39.7)		
Presence of associated diseases						
Hypertension	33 (97.1)			42 (72.4)		
Overweight or obese	27 (79.4)			39 (67.2)		
Dyslipidemia	24 (70.6)			25 (41.3)		
Diabetes mellitus	20 (58.8)			9 (15.5)		
Hypothyroidism	4 (11.8)			8 (13.8)		
Lifestyles						
Past smoking	18 (52.9)			24 (41.4)		
Active smoking	4 (11.8)			6 (10.3)		
Use of psychotropic drugs						
No	27 (79.4)			36 (62.1)		
Yes	7 (20.6)			22 (37.9)		
Surgery suspension						
Surgeries suspended and rescheduled at least once	9 (26.5)			11(19.0)		
Surgeries suspended and rescheduled more than twice	3 (8.8)			3 (5.2)		

* Coronary artery bypass graft;

** Mean (standard deviation).

Regarding CABG suspension, nine patients (26.5%) had their surgeries suspended and rescheduled at least once, and three patients (8.8%) had their surgeries suspended and rescheduled more than twice. The reasons for suspension were lack of material (n=2; 11.1%), energy failure in the operating room (n=3; 16.6%), emergencies (n=7; 38.8%) and complications with patients (n=6; 33.3%).

Regarding surgery suspension in patients with valve disease, 11 (19.0%) had their surgeries suspended and rescheduled at least once; three patients (5.2%) had their surgeries suspended and rescheduled twice; and three patients had their surgeries suspended and rescheduled more than twice (5.2%). The reasons for suspension were lack of material (n=3; 11.1%), power outages in the operating room (n=4; 14.8%), emergencies (n=12; 44.4%) and complications with patients (n=8; 29.6%).

Table 2 shows the length of stay and first return, in days, of patients undergoing CABG and patients undergoing surgical repair of valvular heart disease.

**Table 2:** Length of hospital stay in days in the preoperative period, in the immediate postoperative period, in the immediate postoperative period, in the total length of hospital stay and time of the first return of patients (34) submitted to coronary artery bypass graft, Ribeirão Preto, São Paulo, Brazil, 2018-2019

	Preoperative period	Immediate postoperative period	Mediate postoperative period	Total	First return
Length of hospital stay - CABG^*^					
Mean (SD)^**^	8.5 (8.7)	4.7 (2.5)	5 (2.4)	18.2 (8.7)	13.8 (5.9)
Median	4	4	5	16	14
Minimum	1	2	1	7	5
Maximum	33	12	11	39	29
Length of hospital stay - Valve repair surgery					
Mean (SD)^**^	6.5 (7.3)	5.3 (5.3)	8.3 (5.2)	20.2 (13.6)	14.8 (4.6)
Median	3	4	7	15.5	15
Minimum	1	2	1	7	4
Maximum	32	40	29	78	24

* Coronary artery bypass graft;

** Standard deviation.

Table 3 shows the medians of CA measurements and their comparison at the three times proposed in this study: preoperative, hospital discharge day and first return after hospital discharge of the 34 patients submitted to CABG.

**Table 3:** Comparison of cardiac anxiety symptoms in patients (92) undergoing coronary artery bypass graft preoperatively, at hospital discharge and at the first outpatient return and the probability values (p) associated with the Friedman test, Ribeirão Preto, São Paulo, Brazil, 2018-2019

Variable	Preoperative period	Discharge	First return
	Median (Min-Max)^*^	Median (Min-Max)^*^	Median (Min-Max)^*^
Cardiac anxiety	34.0 (4 - 50)	38.0 (25 - 54)	38.5 (18 - 48)
*p* ^†^ = 0.008			
CAQ domains^‡^			
Fear and hypervigilance	18.5 (2 - 30)	19.0 (6 - 34)	18 (5 - 32)
*p* = 0.154			
Avoidance	16.0 (0 - 20)	20.0 (8 - 20)	20.0 (2 - 20)
*p* ^†^ = **0.001**			

* Minimum-Maximum;

† Statistical significance;

‡ Cardiac Anxiety Questionnaire domains.

The effect of time on CA symptoms of patients undergoing CABG can be observed both in the total score and in the “Avoidance” domain. The multiple comparison test showed that values of total CA symptoms in the preoperative period and on the day of discharge were different, with statistical significance (p=0.027), as well as in the preoperative period and in the first hospital return (p=0.023). In other words, patients had more CA symptoms on the day of discharge, when compared to the preoperative period, as well as more symptoms on the day of the first return visit, when compared to the preoperative period.

As for the “Avoidance” domain, the values found in the preoperative period and at discharge, as well as in the preoperative period and return, were different with statistical significance, p=0.027 and p=0.039, respectively. Thus, patients presented more CA symptoms on the day of hospital discharge and on the first return visit, when compared to preoperatively.

Table 4 shows the medians of CA measurements and their comparison at the three times proposed in this study: preoperative period, day of hospital discharge and first return visit after hospital discharge of the 58 patients who underwent surgical correction of valvular heart disease.

**Table 4:** Comparison of cardiac anxiety symptoms in patients (92) who underwent valve repair surgery in the preoperative period, at hospital discharge and at the first outpatient return visit and the probability (p) values associated with the Friedman test, Ribeirão Preto, São Paulo, Brazil, 2018-2019

Variable	Preoperative period	Discharge	First return
	Median (Min-Max)^*^	Median (Min-Max)^*^	Median (Min-Max)^*^
Cardiac anxiety	36.0 (7 - 55)	39.0 (16 - 50)	38.5 (13 - 56)
*p* ^†^ = 0.040			
CAQ domains^‡^			
Fear and hypervigilance	21.5 (2 - 35)	20.0 (4 - 31)	19.5 (5 - 36)
*p* = 0.687			
Avoidance	16.0 (0 - 20)	20.0 (0 - 20)	20.0 (2 - 20)
*p* ^†^ = 0.002			

* Minimum-Maximum;

† Statistical significance;

‡ Cardiac Anxiety Questionnaire domains.

The effect of time on CA symptoms of patients undergoing surgical repair of valvular heart disease was also observed, both in the total score and in the “Avoidance” domain. The multiple comparison test showed that the total CA symptom scores in the preoperative period and in the first hospital return were different, with statistical significance (p=0.042), as well as in the “Avoidance” domain (p=0.021). Patients had more CA symptoms at the first return visit when compared with patients with preoperative symptoms.

As for the “Avoidance” domain, the values found in the preoperative period and in the first hospital return were different, with statistical significance (p=0.021). Thus, patients presented more CA symptoms on the day of the first return visit, when compared with those in the preoperative period.

As for the CAQ internal consistency assessment, we found a Cronbach’s Alpha Coefficient of 0.81 in the preoperative period, 0.70 on the day of hospital discharge and 0.75 on the day of the first return, data that showed a good internal consistency of the questionnaire at the three times investigated.

## DISCUSSION

In the present study, we found the effect of time on CA symptoms both in patients undergoing CABG and in patients undergoing valve repair surgery. Overall, all patients, regardless of the type of surgery, presented greater CA symptoms in the PO. This reinforces the need for Brazilian longitudinal studies, given that we did not find in the literature studies that investigated the CA of patients undergoing CABG and surgical repair of valvular heart disease at the three times investigated in the present study.

We found a study conducted in Germany, in which researchers assessed the CA of 90 patients undergoing CABG (n=60), valve replacement (n=22) or combined surgeries (n=8) at three times: preoperatively, two weeks before surgery, six weeks after surgery and six months after surgery. Data were analyzed considering the total sample, which revealed a decrease in symptoms six weeks and six months after surgery, when compared to the preoperative period (p<0.001). As for the “Fear” domain, patients showed improvement in symptoms in the PO, when compared with those in the preoperative period, but with no difference between the times, six weeks and six months after the surgical procedure. Regarding the “Avoidance” domain, there was no difference in the preoperative period and six weeks, but patients showed improvement in symptoms six months after surgery. The authors did not find differences in the perception of symptoms at the three times in the “Attention” domain^(13)^.

It is worth mentioning that the original instrument contained 18 items, and these items were distributed in three domains, “Fear”, “Avoidance” and “Attention”, in addition to being possible to assess the overall sum of items. In the validity process for Brazil, the instrument remained with 14 items, and “Fear” and “Attention” domains were combined.

In the present study, patients presented greater CA symptoms on the day of discharge and on the most recent PO, whereas, in a German study^(13)^, the results showed that these symptoms subsided over the long term. These results may reflect the patients’ adaptation to their new health condition after surgery, as surgical treatment is expected to improve the physical symptoms caused by underlying diseases.

Another important issue to be highlighted regarding the greater CA symptoms on the day of discharge and on the first return refers to the routine of the hospital in which the data were collected, since all the guidelines about care in the late PO at home and the importance of outpatient follow-up are passed on only at the time of hospital discharge.

Regarding the presence of greater CA symptoms in patients on the day of the first return visit, when compared to the preoperative period, in the “Avoidance” domain, when we return to the items that specifically make up this domain, such as “I avoid exertion, I take it easy as much as possible, I avoid exercising or other physical activities, I avoid activities that speed up my heart, I avoid activities that make me sweat”, it is noteworthy that, in the hospital of the study, the cardiac rehabilitation service does not absorb 100% of these patients. All guidelines related to effort and resumption of physical activities are provided on the day of hospital discharge and reinforced on the first return visit, factors that may have contributed to the worsening of these symptoms in the PO. Most of this information is provided by doctors and physical therapists.

We also found a study carried out in Bahia, in northeastern Brazil, whose objective was to investigate CA symptoms in 25 patients undergoing CABG, mitral and/or aortic valve replacement and correction of atrial septal defect in the preoperative period and seven days postoperatively. Patients had more CA symptoms in the preoperative period (mean=50; SD=17), when compared with patients who had it in the PO period (mean=29; SD=11), with a significant difference (p=0.001), unlike results found in this research^(17)^.

Given the scarcity of longitudinal studies on CA symptoms in patients undergoing cardiac surgery, as well as the different results found, further research is needed to elucidate the role of this specific type of patient anxiety in the perioperative period of these surgeries.

We also found, in the literature, a German study that investigated CA in patients undergoing cardiac surgery, in order to compare quality of life, symptoms of anxiety, depression and CA of 166 young patients, who underwent three types of valve repair surgery: valve repair, valve replacement by mechanical prosthesis and Ross operation (pulmonary autograft). Patients undergoing valve replacement by mechanical prosthesis had a higher score in the “Fear” domain, when compared with patients undergoing Ross operation. Regarding the “Attention” domain, patients who underwent repair and the Ross operation had a higher score when compared with patients who underwent replacement with a mechanical prosthesis. No significant differences were found in the “Avoidance” domain^(18)^.

In a study carried out in Pernambuco, in northeastern Brazil, aiming at assessing the CA of 91 patients who had undergone cardiac surgery within a period of up to five years, the authors did not specify the type of cardiac surgery^(19)^. Moreover, they used the translated version of the instrument, so the results contain 18 items^(20)^. The authors used a cut-off point to classify patients as “mild to moderate” and “moderate to severe” symptoms. There were no associations of CA symptoms with age group and sex^(19)^.

### Study limitations

The results found require careful interpretation, since it is a non-probabilistic sample, which does not allow extrapolating the comparative data presented here to all patients with heart disease, regardless of the severity of the disease. Another limitation is in the times investigated, since, as the last collection ended on the day of the first return, we are not aware of CA symptoms in the long term, as evidenced in some studies in the literature.

### Contributions to nursing

Considering that the presence of CA symptoms for a long time can impair physical recovery and psychosocial rehabilitation^(5,12,15,21)^, we believe that early identification of CA symptoms is important, as these patients are at higher risk for serious adverse cardiac events^(10,12-13,22)^. From this perspective, actions aimed at providing information at admission, throughout hospitalization, at discharge and at the outpatient return by the care team can help to minimize CA symptoms, improve health-related quality of life and avoid potential mediate and late postoperative complications in patients undergoing cardiac surgery.

## CONCLUSION

We concluded that patients undergoing CABG had more symptoms of total CA and in the “Avoidance” domain on the day of hospital discharge, when compared to preoperative patients, as well as presented greater CA symptoms on the day of the first return, when compared with preoperative patients. Patients undergoing valve repair surgeries had greater symptoms of total CA and in the “Avoidance” domain in the first hospital return, when compared with those in the preoperative period.

SUPPLEMENTARY MATERIAL

Kazitani BS. *Ansiedade cardíaca no perioperatório de cirurgias de revascularização do miocárdio e de valve repair surgery* [dissertation]. Ribeirão Preto: Universidade de São Paulo, Escola de Enfermagem de Ribeirão Preto; 2020 [citado 2022-05-02]. https://doi.org/10.11606/D.22.2020.tde-17032021-093121

0034-7167-reben-76-01-e20220250-sup01

## References

[1] Ministério da Saúde (BR), Secretaria de Atenção Primária à Saúde, Departamento de Promoção da Saúde (2022). Estratégia de Saúde Cardiovascular na Atenção Primária à Saúde: instrutivo para profissionais e gestores.

[2] Ministério da Saúde (BR), Secretaria Executiva, Datasus (2019). Informações de Saúde. Informações epidemiológicas e morbidade.

[3] Silva FL, Melo MAB, Neves RA (2019). Perfil clínico-epidemiológico dos pacientes internados por infarto agudo do miocárdio em hospital de Goiás. RBMC.

[4] Fiksdal A, Hanlin L, Kuras Y, Gianferante D, Chen X, Toma MV (2019). Associations between symptoms of depression and anxiety and cortisol responses to and recovery from acute stress. Psychoneuroendocrinol.

[5] Botzet K, Dalyanoglu H, SchäferR R, Lichtenberg A, Schipke JD, Korbmacher B (2018). Anxiety and depression in patients undergoing mitral valve surgery: a prospective clinical study. Thorac Cardiovasc Surg.

[6] Chen Y, Fu G, Liang F, Wei J, He J, Bai J (2019). Symptoms, hope, self-management behaviors, and quality of life among Chinese preoperative patient with symptomatic valvular heart diseases. J Transcult Nurs.

[7] Rodrigues HF, Furuya RK, Dantas RAS, Morelato RDC, Dessotte CAM (2020). Relationship between emotional states before cardiac valve surgeries with postoperative complications. Rev Gaúcha Enferm.

[8] Eifert GH, Thompson RN, Zvolensky MJ, Edwards K, Frazer NL, Haddad JW (2000). The Cardiac Anxiety Questionnaire: development and preliminary validity. Behav Res Ther.

[9] Salzmann S, Salzmann-Djufri M, Wilhelm M, Euteneuer F (2020). Psychological preparation for cardiac surgery. Curr Cardiol Rep.

[10] Hohls JK, Beer K, Arolt V, Haverkamp W, Kuhlmann SL, Martus P (2020). Association between heart-focused anxiety, depressive symptoms, health behaviors and healthcare utilization in patients with coronary heart disease. J Psychosom Res.

[11] Murphy B, Grande ML, Alvarenga M, Worcester M, Jackson A (2020). Anxiety and depression after a cardiac event: prevalence and predictors. Front Psychol.

[12] Tremblay MA, Denis I, Turcotte S, Fleet R, Archambault P, Dionne CE (2018). Heart-focused anxiety and health care seeking in patients with non-cardiac chest pain: a prospective study. Gen Hosp Psychiatry.

[13] Hoyer J, Eifert GH, Einsle F, Zimmermann K, Krauss S, Knaut M, Matschke K (2008). Heart-focused anxiety before and after cardiac surgery. J Psychosom Res.

[14] Silva LDC, Melo MVP, Rolim ILTP, Dias RS (2018). Intervenções de enfermagem em pacientes da unidade de terapia intensiva cardiológica de um hospital universitário submetidos à cirurgia de revascularização do miocárdio. J Manag Prim Health Care.

[15] Sardinha A, Nardi AE, Araújo CGS, Ferreira MC, Eifertt GH (2013). Validação da versão Brasileira do Questionário de Ansiedade Cardíaca. Arq Bras Cardiol.

[16] Fayers PM, Machin D (2016). Quality of Live: the assessment, analisys and interpretation of patient-report outcomes.

[17] Cordeiro ALL, Freire L, Mendes R, Bastos A, Carvalho S, Melo T (2015). Aplicação do questionário de ansiedade cardíaca no pós-operatório de cirurgia cardíaca. RBPFEX.

[18] Aicher D, Holz A, Feldner S, Köllner V, Schäfers H-J (2011). Quality of life after aortic valve surgery: Replacement versus Reconstruction. J Thorac Cardiovasc Surg.

[19] Moraes VCS, Sobreira BCU, Pinto TAS, Santos AAS, Correia AF (2013). Avaliação da ansiedade cardíaca no pós-operatório de cirurgia cardíaca. Rev Bras Neurol Psiquiatr.

[20] Sardinha A, Nardi AE, Eifert GH (2008). Tradução e Adaptação transcultural da Versão Brasileira do Questionário de Ansiedade Cardíaca. Rev Psiquiatr RGS.

[21] Petersen J, Vettorazzi E, Winter L, Schmied W, Kindermann I, Schäfers H-J (2016). Physical and mental recovery after conventional aortic valve surgery. J Thorac Cardiovasc Surg.

[22] Van Beek MHC, Zuidersma M, Lappenschaar M, Pop G, Roest AM, Van Balkom AJLM (2016). Prognostic association of cardiac anxiety with new cardiac events and mortality following myocardial infarction. Br J Psychiatry.

