# Supplementary material for: Cardiac anxiety in the perioperative period of patients undergoing cardiac surgical procedures: an observational study
Source: Rev Bras Enferm. 2022 Dec 16;76(1):e20220250. doi: 10.1590/0034-7167-2022-0250 (PMC9749766; doi:10.1590/0034-7167-2022-0250)
Supplement: 0034-7167-reben-76-01-e20220250-sup01 [file 0034-7167-reben-76-01-e20220250-sup01.pdf]

UNIVERSIDADE DE SÃO PAULO
ESCOLA DE ENFERMAGEM DE RIBEIRÃO PRETO

BRUNA SONEGO KAZITANI

Ansiedade cardíaca no perioperatório de cirurgias de revascularização do miocárdio e de correção de valvopatias

Ribeirão Preto
2020

BRUNA SONEGO KAZITANI

Ansiedade cardíaca no perioperatório de cirurgias de revascularização do miocárdio e de correção de valvopatias

Dissertação apresentada à Escola de Enfermagem de Ribeirão Preto da Universidade de São Paulo, para obtenção do título de Mestre em Ciências, Programa de Enfermagem Fundamental.

Linha de pesquisa: O cuidar de adultos e idosos.

Orientadora: Profa. Dra. Carina Aparecida Marosti Dessotte

Ribeirão Preto

2020

Autorizo a reprodução e divulgação total ou parcial deste trabalho, por qualquer meio convencional ou eletrônico, para fins de estudo e pesquisa, desde que citada a fonte.

Kazitani, Bruna Sonego

Ansiedade cardíaca no perioperatório de cirurgias de revascularização do miocárdio e de correção de valvopatias / Bruna Sonego Kazitani; orientadora, Carina Aparecida Marosti Dessotte. – 2020

87 f. : il. ; 30 cm

Dissertação (Mestrado em Enfermagem) – Programa de Pós-Graduação em Enfermagem Fundamental, Escola de Enfermagem de Ribeirão Preto, Universidade de São Paulo, Ribeirão Preto, 2020

1. Ansiedade Cardíaca. 2. Depressão. 3. Ansiedade. 4. Procedimentos Cirúrgicos Cardíacos. 5. Enfermagem

Nome: KAZITANI, Bruna Sonego

Título: Ansiedade cardíaca no perioperatório de cirurgias de revascularização do miocárdio e de correção de valvopatias

Dissertação apresentada à Escola de Enfermagem de Ribeirão Preto da Universidade de São Paulo, para obtenção do título de Mestre em Ciências, Programa de Enfermagem Fundamental.

Aprovado em: _____/_____/______

Banca Examinadora

Prof. Dr. ____

Instituição: ____

Julgamento: ____

Prof. Dr. ____

Instituição: ____

Julgamento: ____

Prof. Dr. ____

Instituição: ____

Julgamento: ____

Prof. Dr. ____

Instituição: ____

Julgamento: ____

Á *minha família*, por sempre estar ao meu lado.

Ao *meu noivo*, por toda compreensão, incentivo e carinho durante todo o desenvolvimento deste projeto.

AGRADECIMENTOS

Aos meu pais, **Marco** e **Lia**, e minha irmã, **Jaqueline**, por estarem ao meu lado durante essa jornada, me apoiando, incentivando e motivando durante todo o processo de desenvolvimento deste projeto.

Ao meu noivo, **Vitor**, por acreditar em mim e por todo companheirismo durante esse momento de minha vida.

Aos meu sogros **Ivone** e **Sérgio**, e ao **Biscoito**, por fazerem parte de minha vida e me proporcionarem momentos de alegria e felicidade.

À minha amiga **Elisa**, pelos cafés, conversas e colaboração durante todo esse período.

À minha colega **Letícia**, por sua ajuda e responsabilidade durante minha coleta de dados.

Aos funcionários do 9º andar, **Ulisses**, **Márcia** e **Patrícia**, por tornarem meus dias de coleta de dados mais alegres e felizes, me ajudando e motivando sempre.

À professora **Drª Rosana** e ao professor **Dr. Alfredo** pela atenção, participação e contribuição ao longo deste projeto.

À minha orientadora professora **Drª Carina**, por todo incentivo e apoio desde o início da idealização deste projeto até sua finalização.

À **Coordenação de Aperfeiçoamento de Pessoal de Nível Superior – Brasil (CAPES)**, pela bolsa concedida durante o desenvolvimento do projeto.

E a todos os **pacientes** que aceitaram participar e doar um tempo de suas vidas, mesmo que muitas vezes estivessem cansados ou chateados, para que essa pesquisa pudesse ser realizada.

RESUMO

KAZITANI, B. S. **Ansiedade cardíaca no perioperatório de cirurgias de revascularização do miocárdio e de correção de valvopatias.** 2020. 87 f. Dissertação (Mestrado). Escola de Enfermagem de Ribeirão Preto, Universidade de São Paulo, Ribeirão Preto, 2020.

Objetivo: Comparar os sintomas de Ansiedade Cardíaca (AC) em pacientes submetidos à cirurgia cardíaca no pré-operatório, no dia da alta hospitalar e no primeiro retorno, e investigar a associação desses sintomas com variáveis sociodemográficas, clínicas e com os sintomas de ansiedade e depressão. **Método:** Estudo observacional analítico, de coorte prospectivo, realizado em um hospital universitário do interior paulista. Uma amostra consecutiva e não probabilística foi constituída por pacientes de ambos os sexos, maiores de 18 anos, submetidos a Cirurgia de Revascularização do Miocárdio (CRM) e cirurgias para correção de valvopatia. Para a avaliação dos sintomas de AC foi utilizado o "Questionário de Ansiedade Cardíaca". A coleta de dados foi realizada por meio de entrevistas individuais e consulta aos prontuários dos participantes em três momentos: T_0 (pré-operatório), T_1 (pós-operatório, no dia da alta hospitalar) e T_2 [primeiro retorno, que ocorre 14 dias (± 7 dias) após alta hospitalar]. Para compararmos os sintomas de AC nos três tempos, utilizamos o teste de Friedman e para investigarmos a correlação dos sintomas de AC com os sintomas de ansiedade e depressão, utilizamos o teste de Correlação de Spearman. O nível de significância foi de 5%. **Resultados**: Participaram do estudo 92 pacientes, sendo 34 submetidos à CRM e 58 a correção cirúrgica de valvopatia. Com relação aos pacientes submetidos a CRM, a média de idade encontrada foi de 62,4 anos, maioria do sexo masculino (64%) e com companheiro (67,6%). Os valores dos postos dos sintomas de AC total foram maiores no dia da alta quando comparados com o pré-operatório, assim como os sintomas de AC no dia do primeiro retorno quando comparados com o pré-operatório. Pacientes idosos apresentaram maiores escores de AC no pré-operatório. Quanto aos sintomas de ansiedade, foi encontrada correlação moderada com os sintomas de AC nos três tempos. Já nos pacientes submetidos a correção cirúrgica de valvopatia, a média de idade encontrada foi de 54,4 anos, maioria do sexo feminino (51,7%) e com companheiro (70,7%). Os valores dos postos dos sintomas de AC total foram maiores no primeiro retorno quando comparados com o pré-operatório. Pacientes inativos no pré-operatório apresentaram maiores escores de AC. Encontramos fraca correlação da AC dos pacientes com sintomas de ansiedade no pré-operatório e correlação moderada com os sintomas de depressão no pré-operatório. **Conclusão:** Pacientes submetidos à CRM apresentaram maior sintomatologia de AC no dia da alta e no primeiro retorno quando comparados com o pré-operatório, e quanto mais sintomas de AC, mais sintomas de ansiedade nos três tempos estudados. Pacientes idosos apresentaram maior sintomatologia de AC. Os pacientes submetidos à correção de valvopatias apresentaram maior sintomatologia de AC no primeiro retorno quando comparada com o pré-operatório, e quanto mais sintomas de AC no pré-operatório, mais sintomas de ansiedade e depressão. Pacientes inativos apresentaram maior sintomatologia de AC.

Descritores: Ansiedade Cardíaca. Depressão. Ansiedade. Procedimentos Cirúrgicos Cardíacos. Enfermagem.

.

ABSTRACT

KAZITANI, B. S. **Cardiac anxiety in the perioperative period of coronary artery bypass grafting and valvular heart disease surgeries**. 2020. 87 pp. Thesis (Master's Degree). Nursing School of Ribeirão Preto, University of São Paulo, Ribeirão Preto. 2020.

Objective: To compare the symptoms of Cardiac Anxiety (CA) in patients who underwent cardiac surgery preoperatively, on the day of hospital discharge and on the first return, and investigate the association of these symptoms with sociodemographic and clinical variables and with the symptoms of anxiety and depression. **Method:** Analytical observational study, with prospective cohort, held in a university hospital in the countryside of São Paulo. A consecutive and non-probabilistic sample consisted of patients of both genders, over 18 years old, who underwent Coronary Artery Bypass Grafting (CABG) and surgeries for correction of valvular heart disease. The "Cardiac Anxiety Questionnaire" was used to assess the symptoms of CA. Data collection took place through individual interviews and consultation of the participants' medical records in three moments: T_0 (preoperative), T_1 (postoperative, on the day of hospital discharge) and T_2 [first return, which happens 14 days (± 7 days) after hospital discharge]. To compare the symptoms of CA in the three stages, we used the Friedman's test; and, to investigate the correlation of the symptoms of CA with the symptoms of anxiety and depression, we used the Spearman's Correlation test. The level of significance was 5%. **Results:** The study was attended by 92 patients, 34 underwent CABG and 58 surgical correction of valvular heart disease. Regarding patients who underwent CABG, the average age found was 62.4 years, mostly male (64%) and with a partner (67.6%). The presented values of the total symptoms of CA were higher on the day of discharge when compared to the preoperative period, as well as the symptoms of CA on the day of the first return when compared to the preoperative period. Elderly patients presented higher preoperative scores of CA. Regarding the symptoms of anxiety, we found a moderate correlation with the symptoms of CA in the three moments. As for the patients who underwent the surgical correction of valvular heart disease, the average age found was 54.4 years, mostly female (51.7%) and with a partner (70.7%). The presented values of the total symptoms of CA were higher in the first return when compared to the preoperative period. Preoperative inactive patients presented higher scores of CA. We found a weak correlation of CA in patients with symptoms of anxiety in the preoperative period and a moderate correlation with symptoms of depression in the preoperative period. **Conclusion:** Patients who underwent CABG presented a greater number of symptoms of CA on the day of discharge and on the first return when compared with the preoperative period; and the more symptoms of CA, the more symptoms of anxiety in the three studied periods. Elderly patients presented a greater number of symptoms of CA. Patients who underwent correction of valvular diseases presented a greater number of symptoms of CA in the first return when compared to the preoperative period; and the more symptoms of CA in the preoperative period, the more symptoms of anxiety and depression. Inactive patients presented a greater number of symptoms of CA.

Descriptors: Cardiac Anxiety; Depression; Anxiety; Cardiac Surgical Procedures; Nursing.

RESUMEN

KAZITANI, B. S. **Ansiedad cardíaca en el período preoperatorio de cirugías de revascularización miocárdica y reparación valvular**. 2020. 87 h. Tesis (Maestría). Escuela de Enfermería de Ribeirão Preto, Universidad de São Paulo, Ribeirão Preto, 2020.

Objetivo: Comparar los síntomas de la Ansiedad Cardíaca (AC) en pacientes sometidos a cirugía cardíaca en el preoperatorio, en el postoperatorio y en el primer regreso, e investigar la asociación de estos síntomas con variables sociodemográficas y clínicas y con los síntomas de ansiedad y depresión. **Método:** Estudio observacional analítico, de cohorte prospectivo, efectuado en un hospital universitario del interior de São Paulo. Una muestra consecutiva y no probabilística compuesta por pacientes de ambos sexos, mayores de 18 años, sometidos a Cirugía de Revascularización Miocárdica (CRM) y cirugías de reparación valvular. Para la evaluación de los síntomas de la AC, se utilizó el "Cuestionario de Ansiedad Cardíaca". La recopilación de datos tuvo lugar mediante entrevistas individuales y consulta de las historias clínicas de los participantes en tres momentos: T_0 (preoperatorio), T_1 (postoperatorio, el día del alta) y T_2 [primer regreso, 14 días (± 7 días) después del alta]. Para comparar los síntomas de la AC en las tres etapas, usamos la prueba de Friedman; y, para investigar la correlación de los síntomas de la AC con los síntomas de ansiedad y depresión, usamos la prueba de Correlación de Spearman. El nivel de significación fue del 5%. **Resultados:** El estudio tuvo la participación de 92 pacientes, siendo que 34 fueron sometidos a CRM y 58 a cirugías de reparación valvular. En lo que atañe a los pacientes sometidos a CRM, la edad promedio encontrada fue de 62,4 años, mayoritariamente varones (64%) y con pareja (67,6%). Los valores del rango total de síntomas de la AC fueron mayores para el postoperatorio en comparación con el preoperatorio, así como los síntomas de la AC para el día del primer regreso en comparación con el preoperatorio. Los pacientes ancianos presentaron puntuaciones mayores de la AC en el preoperatorio. En cuanto a los síntomas de ansiedad, se encontró una correlación moderada con los síntomas de la AC en los tres momentos. En los pacientes sometidos a cirugías de reparación valvular, la edad promedio encontrada fue de 54,4 años, mayoritariamente mujeres (51,7%) y con pareja (70,7%). Los valores del rango total de síntomas de la AC fueron mayores en el primer regreso en comparación con el preoperatorio. Los pacientes inactivos presentaron puntuaciones mayores de la AC en el preoperatorio. Se encontró una escasa correlación de la AC de los pacientes con síntomas de ansiedad en el preoperatorio y una correlación moderada con síntomas de depresión en el preoperatorio. **Conclusión:** Los pacientes sometidos a CRM presentaron más síntomas de la AC en el postoperatorio y en el primer regreso en comparación con el preoperatorio; y a más síntomas de la AC, más síntomas de ansiedad en los tres momentos estudiados. Los pacientes ancianos presentaron más síntomas de la AC. Los pacientes sometidos a cirugías de reparación valvular presentaron más síntomas de la AC en el primer regreso en comparación con el preoperatorio; y a más síntomas de la AC en el preoperatorio, más síntomas de ansiedad y depresión. Los pacientes inactivos presentaron más síntomas de la AC.

Descriptores: Ansiedad Cardíaca; Depresión; Ansiedad; Procedimientos Quirúrgicos Cardíacos; Enfermería.

LISTA DE TABELAS

Tabela 1 -	Relação dos motivos das perdas da coleta de dados, do momento do óbito e da descontinuação dos pacientes submetidos à cirurgia de revascularização do miocárdio e cirurgia para correção de valvopatia Ribeirão Preto, 2018 – 2019	38
Tabela 2 -	Caracterização sociodemográfica dos 34 pacientes submetidos à cirurgia de revascularização do miocárdio, segundo sexo, idade, situação conjugal, escolaridade, renda mensal, número de pessoas que dependem da renda e situação profissional. Ribeirão Preto, 2018 – 2019	39
Tabela 3 -	Caracterização clínica dos 34 pacientes submetidos à cirurgia de revascularização do miocárdio, segundo a presença de doenças associadas e hábitos de vida. Ribeirão Preto, 2018 – 2019	40
Tabela 4 -	Caracterização sociodemográfica dos 58 pacientes submetidos a cirurgia para correção de valvopatia segundo sexo, idade, situação conjugal, escolaridade, renda mensal, número de pessoas que dependem da renda e situação profissional. Ribeirão Preto, 2018 – 2019	41
Tabela 5 -	Caracterização clínica dos 58 pacientes submetidos à cirurgia para correção de valvopatia segundo a presença de doenças associadas e hábitos de vida. Ribeirão Preto, 2018 – 2019	42
Tabela 6 -	Tempo de internação em dias no pré-operatório, no pós-operatório imediato, no pós-operatório mediato, na internação total e tempo do primeiro retorno dos 34 pacientes submetidos à cirurgia de revascularização do miocárdio. Ribeirão Preto, 2018-2019	43
Tabela 7 -	Tempo de internação em dias no pré-operatório, no pós-operatório imediato, no pós-operatório mediato, na internação total e tempo do primeiro retorno dos 58 pacientes submetidos a cirurgia para correção de valvopatia. Ribeirão Preto, 2018-2019	43
Tabela 8 -	Comparação das medianas dos sintomas de ansiedade cardíaca dos 34 pacientes submetidos à cirurgia de revascularização do miocárdio no pré-operatório, dia da alta e no primeiro retorno após alta hospitalar e os valores de probabilidade (*p*) associados ao teste de Friedman. Ribeirão Preto, 2018 – 2019	44

Tabela 9 - Descrição dos valores do qui-quadrado e graus de liberdade do teste de Friedman referente aos 34 pacientes submetidos à cirurgia de revascularização do miocárdio. Ribeirão Preto, 2018 – 2019.................. 45

Tabela 10 - Comparação das medianas dos sintomas de ansiedade cardíaca dos 58 pacientes submetidos à cirurgia para correção de valvopatia no pré-operatório, dia da alta e no primeiro retorno após alta hospitalar e os valores de probabilidade (*p*) associados ao teste de Friedman. Ribeirão Preto, 2018-2019... 46

Tabela 11 - Descrição dos valores do qui-quadrado e graus de liberdade do teste de Friedman referente aos 58 pacientes submetidos à cirurgia para correção de valvopatia. Ribeirão Preto, 2018 – 2019.. 46

Tabela 12 - Análise descritiva dos sintomas de ansiedade cardíaca no pré-operatório dos 34 pacientes submetidos à cirurgia de revascularização do miocárdio, conforme sexo, idade, situação conjugal, situação profissional, uso de psicotrópicos em casa e valores de probabilidade (*p*) associados ao teste de Mann-Whitney. Ribeirão Preto, 2018-2019... 47

Tabela 13 - Análise descritiva dos sintomas de ansiedade cardíaca no pré-operatório dos 58 pacientes submetidos à cirurgia para correção de valvopatia, conforme sexo, idade, situação conjugal, situação profissional, uso de psicotrópicos em casa e valores de probabilidade (*p*) associados ao teste de Mann-Whitney. Ribeirão Preto, 2018-2019....................................... 48

Tabela 14 - Análise descritiva dos sintomas de ansiedade cardíaca no primeiro retorno hospitalar dos 34 pacientes submetidos à cirurgia de revascularização do miocárdio, conforme sexo, idade, situação conjugal, situação profissional e valores de probabilidade (*p*) associados ao teste de Mann-Whitney. Ribeirão Preto, 2018-2019....................................... 49

Tabela 15 - Análise descritiva dos sintomas de ansiedade cardíaca no primeiro retorno hospitalar dos 58 pacientes submetidos à cirurgia para correção de valvopatia, segundo sexo, idade, situação conjugal, situação profissional e valores de probabilidade (*p*) associados ao teste de Mann-Whitney. Ribeirão Preto, 2018-2019.. 50

Tabela 16 - Correlação dos sintomas de ansiedade cardíaca com os sintomas de ansiedade dos 34 pacientes submetidos à cirurgia de revascularização do miocárdio com os respectivos valores de probabilidade (*p*) associados ao teste de correlação de Spearman. Ribeirão Preto, 2018-2019.. 51

Tabela 17 - Correlação dos sintomas de ansiedade cardíaca com os sintomas de depressão dos 34 pacientes submetidos à cirurgia de revascularização do miocárdio com os respectivos valores de probabilidade (*p*) associados ao teste de correlação de Spearman. Ribeirão Preto, 2018-2019.. 51

Tabela 18 - Correlação dos sintomas de ansiedade cardíaca com os sintomas de ansiedade dos 58 pacientes submetidos à cirurgia para correção de valvopatia com os respectivos valores de probabilidade (*p*) associados ao teste de correlação de Spearman. Ribeirão Preto, 2018-2019.. 52

Tabela 19 - Correlação dos sintomas de ansiedade cardíaca com os sintomas de depressão dos 58 pacientes submetidos à cirurgia para correção de valvopatia com os respectivos valores de probabilidade (*p*) associados ao teste de correlação de Spearman. Ribeirão Preto, 2018-2019.............. 52

LISTA DE SIGLAS

AC	Ansiedade Cardíaca
AE	Angina Estável
AI	Angina Instável
ARS	Ansiedade Relacionada à Saúde
ATP	Angioplastia Transluminal Percutânea
CEC	Circulação Extracorpórea
CRM	Cirurgia de Revascularização do Miocárdio
DAC	Doença Arterial Coronariana
DCV	Doenças Cardiovasculares
FEVE	Fração de Ejeção de Ventrículo Esquerdo
HADS	Hospital Anxiety and Depression Scale
HAS	Hipertensão Arterial Sistêmica
IAM	Infarto Agudo do Miocárdio
PO	Pós-Operatório
QAC	Questionário de Ansiedade Cardíaca
SAG	Síndrome da Adaptação Geral
SNA	Sistema Nervoso Autônomo
UTI	Unidade de Terapia Intensiva

SUMÁRIO

1. INTRODUÇÃO 15
1.1 JUSTIFICATIVA E RELEVÂNCIA DO ESTUDO 15
1.2 REVISÃO DA LITERATURA 16
1.2.1 Doenças cardiovasculares 16
1.2.1.1 Perfil do paciente coronariopata 17
1.2.1.2 Perfil do paciente valvopata 18
1.2.2 Sintomas de ansiedade cardíaca e sua repercussão biopsicossocial 19
1.2.3 Instrumento de avaliação dos sintomas de ansiedade cardíaca 23
1.2.4 Ansiedade cardíaca no perioperatório de cirurgias cardíacas 25
1.3 QUESTÕES DO ESTUDO E HIPÓTESES A SEREM TESTADAS 26
2. OBJETIVOS 28
3. MÉTODO 29
3.1 DELINEAMENTO DO ESTUDO 29
3.2 LOCAL E CASUÍSTICA 29
3.3 ASPECTOS ÉTICOS 30
3.4 PROCEDIMENTOS PARA COLETA DE DADOS 30
3.5 LINHA DO TEMPO REFERENTE AOS TRÊS MOMENTOS DE COLETA DE DADOS 31
3.6 INSTRUMENTOS DE COLETA DE DADOS 32
3.6.1 Instrumento de caracterização sociodemográfica e clínica 32
3.6.2 Instrumento para avaliação dos sintomas de ansiedade cardíaca 33
3.6.3 Instrumento para avaliação dos sintomas de ansiedade e depressão 34
3.7 PROCESSAMENTO E ANÁLISE DOS DADOS 34
4. RESULTADOS 36
4.1 CARACTERIZAÇÃO SOCIODEMOGRÁFICA E CLÍNICA DOS PACIENTES NO PRÉ-OPERATÓRIO 38
4.2 COMPARAÇÃO DOS SINTOMAS DE ANSIEDADE CARDÍACA, NO PRÉ-OPERATÓRIO, NO DIA DA ALTA E NO PRIMEIRO RETORNO APÓS ALTA HOSPITALAR DOS PACIENTES 44
4.3 RELAÇÃO DOS SINTOMAS DE ANSIEDADE CARDÍACA NO PRÉ-OPERATÓRIO E NO PRIMEIRO RETORNO HOSPITALAR COM AS CARACTERÍSTICAS SOCIODEMOGRÁFICAS E CLÍNICAS DOS PACIENTES 47
4.4 CORRELAÇÃO DOS SINTOMAS DE ANSIEDADE CARDÍACA COM OS SINTOMAS DE ANSIEDADE E DEPRESSÃO DOS PACIENTES NO PRÉ-

OPERATÓRIO, NO DIA DA ALTA HOSPITALAR E NO PRIMEIRO RETORNO APÓS A ALTA HOSPITALAR 51
5. DISCUSSÃO 55
5.1 LIMITAÇÕES DO ESTUDO 64
5.2 CONTRIBUIÇÕES DO ESTUDO PARA A PRÁTICA CLÍNICA 64
6. CONCLUSÃO 66
REFERÊNCIAS 67
APÊNDICES 81
ANEXOS 85

1. INTRODUÇÃO

1.1 JUSTIFICATIVA E RELEVÂNCIA DO ESTUDO

Os pacientes que aguardam a cirurgia cardíaca podem vivenciar altos níveis de sintomas de ansiedade devido a medos, preocupações e incertezas sobre a cirurgia (GUO et al., 2015). Estudos têm demonstrado que sintomas de ansiedade têm influenciado a recuperação fisiológica no pós-operatório (PO) de cirurgias cardíacas (PINTON et al., 2006; ASSIS et al., 2014), além de potencializar a ocorrência de complicações (TULLY; BAKER, 2012; STENMAN; HOLZMANN; SARTIPY, 2013; TULLY; NEWLAND; BAKER, 2015; KALOGIANNI et al., 2016), aumentar o tempo de internação (STENMAN; HOLZMANN; SARTIPY, 2013), aumentar o número de reinternações hospitalares (BLUMENTHAL et al., 2003; STENMAN; HOLZMANN; SARTIPY, 2013), diminuir a eficácia de programa de reabilitação cardíaca (SZCZEPANSKA-GIERACHA et al., 2012) e aumentar a mortalidade pós-operatória (STENMAN; HOLZMANN; SART IPY, 2013; WILLIAMS et al., 2013).

Até o momento, encontramos poucos estudos desenvolvidos com o objetivo de investigar a presença de sintomas de ansiedade cardíaca no perioperatório de cirurgias cardíacas (HOYER et al., 2008; MORAES et al., 2013). A ansiedade cardíaca é definida como o medo a estímulos e sensações relacionadas às doenças cardíacas e que são percebidos como negativos ou perigosos. A ansiedade cardíaca potencializa a ansiedade porque os eventos cardíacos são percebidos pelos pacientes como aversivos e perigosos. Ela está diretamente relacionada com o medo, sensações e funcionalidade dos eventos cardíacos, como infarto agudo do miocárdio e arritmias graves (EIFERTT et al., 2000).

Muitos pacientes têm vivenciado estressores de ordem psicológica no perioperatório de cirurgias cardíacas (KRANNICHI et al., 2007; TORRATI, 2009; CSERÉP et al., 2012; KELPIS et al., 2013; POOLE et al., 2014; TULLY; NEWLAND; BAKER, 2015; KAZITANI et al., 2018), o que denota a importância da avaliação da condição emocional destes pacientes (STENMAN; HOLZMANN; SARTIPY, 2013).

O profissional de enfermagem pode auxiliar o paciente no enfrentamento da doença cardíaca e no processo cirúrgico (KOERICH et al., 2013), contribuindo tanto para o aumento do conforto psicológico quanto para melhores resultados clínicos e cirúrgicos (KALOGIANNI et al., 2016; WILLIAMS et al., 2013).

Frente ao exposto, o enfermeiro necessita de conhecimentos técnico-científicos sobre a fisiopatologia dos estressores psicoemocionais envolvidos, como os sintomas de ansiedade cardíaca, para realizar o planejamento da assistência de enfermagem perioperatória aos pacientes com doença cardíaca. A identificação precoce da presença desses sintomas, bem como o entendimento de sua influência no quadro clínico do paciente, possibilitará intervenções de enfermagem adequadas para favorecer sua recuperação.

Considerando que o perfil do paciente a ser submetido à cirurgia de revascularização do miocárdio difere do perfil do paciente submetido ao tratamento cirúrgico das doenças valvares, investigamos a AC dos dois grupos separadamente.

1.2 REVISÃO DA LITERATURA

1.2.1 Doenças cardiovasculares

As Doenças Cardiovasculares (DCV) lideram as causas de óbitos globalmente com 17,9 milhões de mortes registradas em 2016, o equivalente a 31% de todas as mortes no mundo (WHO, 2018).

No Brasil, em 2013, a proporção de óbitos por DCV em todas as faixas etárias foi de 29,88%, sendo a principal causa a partir dos 40 anos. No ano de 2016, o número de óbitos por todas as doenças do aparelho circulatório foi de 339.628 no Brasil e 82.295 no Estado de São Paulo, o maior índice de mortalidade do país por estas causas (BRASIL, 2016).

A elevada incidência de DCV pode ser justificada pelos fenômenos urbanos, sociais e econômicos que ocorreram ao longo do século XX e que tiveram impacto tanto no surgimento como na disseminação dos clássicos fatores de risco para as DCV (LOTUFO, 2015).

As DCV são associadas a quatro fatores de risco principais, sendo eles o tabagismo, o uso abusivo do álcool, o sedentarismo e a alimentação inadequada (com elevado teor de caloria e sódio), que são hábitos que geram, na maioria das vezes, excesso de peso, que por sua vez aumenta significativamente o risco de Hipertensão Arterial Sistêmica (HAS), dislipidemias, diabetes mellitus e doença aterosclerótica (SARAIVA; GAGLIARDI, 2015). Os grupos de pessoas mais suscetíveis à apresentação dos referidos fatores de risco são as pessoas com baixa renda, baixa escolaridade e idosos (SARAIVA; GAGLIARDI, 2015).

Das inúmeras apresentações clínicas das DCV, enfocaremos, neste estudo, os pacientes cardiopatas submetidos ao tratamento cirúrgico da Doença Arterial Coronariana (DAC), à

cirurgia de revascularização do miocárdio (CRM) e à correção cirúrgica das doenças valvares (plastia e/ou troca de valva).

1.2.1.1 Perfil do paciente coronariopata

Podemos conceituar a DAC como uma alteração funcional e/ou anatômica das artérias coronárias, o que pode causar isquemia miocárdica em decorrência da diminuição da oferta de oxigênio ou do aumento da demanda do miocárdio. A DAC pode se manifestar através de sintomas, como dor precordial, ou permanecer assintomática (REGGI; STEFANINI; CARVALHO, 2015). De acordo com Reggi e colaboradores (2015), dentre as inúmeras situações clínicas que podem acarretar a descompensação da demanda e da oferta de oxigênio, a aterosclerose pode ser identificada com uma das mais comuns. A DAC pode se manifestar por meio do Infarto Agudo do Miocárdio (IAM) sem supradesnivelamento do segmento ST, IAM com supradesnivelamento do seguimento ST, Angina Instável (AI) ou Angina Estável (AE) (BAGNATORI, 2009).

Silva, Melo e Neves (2019) realizaram um estudo retrospectivo com o objetivo de traçar o perfil de pacientes internados por IAM a partir da revisão de prontuários de 64 pacientes internados com esse diagnóstico de 01 de agosto de 2016 a 31 de julho de 2017 em um hospital de Goiás. De acordo com os resultados encontrados, houve predomínio do sexo masculino (64,5%), com faixa etária de maior frequência entre 50 a 59 anos para os homens e 60 a 69 para as mulheres, sendo que as comorbidades mais notadas foram hipertensão arterial sistêmica (76,2%), dislipidemia (42,8%) e diabetes mellitus (38,1%). Além disso, 67,39% dos pacientes possuíam histórico de tabagismo ativo ou eram tabagistas pregressos. Quanto aos sintomas, precordialgia foi o mais relatado pelos pacientes (98,4%), seguido de dispneia (40,6%).

Em outro estudo retrospectivo, realizado por Janssen e colaboradores (2015), em um hospital do Maranhão, foram analisados prontuários de pacientes que foram submetidos à CRM no período de 2011 a 2013, com o objetivo de investigar características sociodemográficas e clínicas, e os pesquisadores obtiveram os seguintes resultados: maior frequência do sexo masculino (68,5%) com faixa etária de 61 a 70 anos, em união estável (74,2%) e que exerciam atividade laboral remunerada (54,2%). Além disso, as comorbidade mais frequentemente identificadas foram hipertensão arterial sistêmica (87,1%), diabetes mellitus (44,2%) e dislipidemia (18,5%). Com relação às manifestações clínicas, destacou-se os seguintes sintomas: dispneia (81,4%), dor precordial (52,8%) e cansaço (11,4%).

Já Silva, L. N. et al. (2018) analisaram o prontuário de 367 atendidos em um pronto-socorro com diagnóstico de Síndrome Coronariana Aguda de julho a setembro de 2015, em um estudo retrospectivo, com o objetivo de identificar o perfil epidemiológico, evolução clínica e desfecho dos pacientes. Houve prevalência de indivíduos do sexo masculino (54,8%) e casados (59,1%). Dentre as comorbidades encontradas, houve predomínio de hipertensão arterial sistêmica (62,4%), dislipidemia (23,7%) e diabetes mellitus (13,9%), sendo que 31% relataram serem tabagistas e 31,9% etilistas. Além disso, dentre os pacientes que permaneceram internados, 13,2% foram a óbito.

Diante do exposto, podemos identificar que os pacientes coronariopatas são, na sua maioria, do sexo masculino e já apresentavam a hipertensão arterial sistêmica, uma condição que eleva o risco de desenvolvimento de novos eventos cardíacos. Além disso, observamos que muitos deles também já apresentavam dislipidemia e diabetes mellitus, bem como o hábito do tabagismo, que são fatores de risco para eventos cardíacos primários e secundários. Assim, observa-se a complexidade clínica dos pacientes coronariopatas.

1.2.1.2 Perfil do paciente valvopata

A principal causa de valvopatia no Brasil ainda é a de etiologia reumática, diferentemente do observado nos Estados Unidos e Europa, por exemplo, nos quais as principais causas são as doenças degenerativas (MORAES, 2013; TARASOUTCHI et al., 2011).

De acordo com Rodrigues e colaboradores (2009), pacientes submetidos à intervenções cirúrgicas para correção de valvopatia poderão apresentar maior comprometimento de sua condição hemodinâmica devido à alta complexidade envolvida no procedimento, pois são necessárias intervenções intracardíacas, as quais envolvem a reconstrução ou a substituição do aparelho valvar acometido, geralmente resultando em prolongamento do tempo de Circulação Extracorpórea (CEC).

É possível encontrar na literatura diversos estudos que avaliaram o perfil destes pacientes. No estudo de Andrade e colaboradores (2010), os autores realizaram a avaliação de 840 prontuários de pacientes que foram submetidos à cirurgia valvar, retrospectivamente, entre janeiro de 2001 e junho de 2009, e encontraram que 25,6% já possuía cirurgia cardíaca prévia, 54,3% eram do sexo feminino, 28% possuía idade acima de 60 anos, 22,4% possuía hipertensão pulmonar e 11,2% possuía Fração de Ejeção do Ventrículo Esquerdo (FEVE) entre 30%-50%.

Outro estudo, com o objetivo de analisar as características sociodemográficas e clínicas de 86 pacientes valvopatas que realizavam acompanhamento ambulatorial foi realizado por Anjos e colaboradores (2016), no qual encontraram que 58,1% pertenciam ao sexo feminino, com idade média de 52,7 anos (DP=12,9), 37,2% possuíam lesão valvar única e 31,4% possuíam dupla lesão valvar. Além disso, evidenciaram que a maior parte dos pacientes (71,3%) realizava tratamento clínico, além de já ter sido submetida a um tratamento cirúrgico.

Já Moraes (2013) realizou a avaliação prospectiva de 540 pacientes submetidos à correção de valvopatias, de fevereiro a dezembro de 2009, em um hospital de grande porte de São Paulo, e encontrou os seguintes resultados: idade média de 56 anos de idade (DP=16), 50% pertenciam ao sexo feminino, 51% possuía hipertensão arterial sistêmica no pré-operatório, 42% possuía fibrilação atrial, 27% possuía disfunção diastólica, 23% possuía disfunção ventricular global e 22% tinham disfunção ventricular direita.

Dessa forma, podemos perceber a mudança no perfil dos pacientes valvopatas que podem receber indicação cirúrgica ao longo do tempo. A indicação cirúrgica ocorre apenas quando não há outro mecanismo que consiga compensar a disfunção anatômica e/ou funcional da válvula acometida e há o aparecimento dos sintomas, e como muitas vezes os pacientes são assintomáticos, pode ocorrer a indicação cirúrgica apenas quando o paciente já apresenta idade avançada, dupla lesão, comorbidades associadas e outras complicações, como, por exemplo, fibrilação atrial (ANDRADE et al., 2010; ANJOS et al., 2016).

Observa-se que o perfil dos pacientes valvopatas difere do perfil dos coronariopatas, uma vez que a maioria dos valvopatas é do sexo feminino, com menos de 60 anos. Porém, ambos apresentam doenças concomitantes diversas, que pioram a funcionalidade cardíaca.

1.2.2 Sintomas de ansiedade cardíaca e sua repercussão biopsicossocial

A ansiedade pode ser definida como uma sensação de mal-estar psíquico caracterizado pelo temor de um perigo iminente, real ou imaginário (GONÇALVES; MEDEIROS, 2016). A ansiedade faz parte da vida, sendo uma reação emocional normal e esperada diante de situações novas e desconhecidas (CABRERA; SPONHOLZ, 2012; NASSER et al., 2016). Quando não é desproporcionalmente intensa, pode melhorar o desempenho, estimular a cooperação e promover soluções criativas. Porém, quando há uma resposta não adequada a um estímulo em razão de sua duração ou intensidade pode ser considerada patológica (CABRERA; SPONHOLZ, 2012).

No hospital, o paciente encontra um universo de ameaças internas e externas que ameaçam a sua integridade corporal por conta dos procedimentos à que é submetido, da exposição de sua intimidade à estranhos, da convivência em um ambiente de doença, da dor e morte e, além disso, do sofrimento pela incerteza quanto à evolução da sua doença (CABRERA; SPONHOLZ, 2012).

A ansiedade pode ser benéfica quando associada ao mecanismo de "luta ou fuga", pois prepara o indivíduo para a situação estressante (GONÇALVES; MEDEIROS, 2016). Entretanto, em situações como em um procedimento anestésico-cirúrgico, essa reação, devido aos mesmos mecanismos (autonômica e ativação endócrina), pode acarretar alterações nos parâmetros dos sinais vitais, como o aumento da frequência cardíaca e respiratória, elevação da pressão arterial sistêmica, sudorese, boca seca, palpitações, vômitos, calafrios e, assim, exacerbar os sintomas somáticos (CABRERA; SPONHOLZ, 2012; GONÇALVES; MEDEIROS, 2016) e interferir no processo de recuperação clínica, com consequente aumento do tempo de internação (CABRERA; SPONHOLZ, 2012; NASSER et al., 2016).

No ano de 1992, Eifert desenvolveu a teoria da cardiofobia e atrelada a essa condição a definição de Ansiedade Cardíaca (AC). Uma diferença importante a ser observada entre a cardiofobia e outros tipos de ansiedade está na natureza dos sintomas, no cardiofóbico prevalece os sintomas de precordialgia quando ele sente medo da morte ou de um iminente ataque cardíaco, destoando do padrão clássico de pessoas ansiosas, nas quais não ocorre essa prevalência. Em outras palavras, a cardiofobia não é apenas uma fobia de doenças cardíacas, é uma complexa interação de precordialgia, resposta autonômica, atenção focada no coração, ansiedade e comportamento de evitação. Assim, o paciente que vivencia a ansiedade cardíaca tende a evitar atividades do dia a dia na tentativa de evitar o desencadeamento de um ataque cardíaco. Muitas vezes, mesmo com o comportamento de evitação, o paciente vivencia sinais e sintomas de um ataque cardíaco quando ele não existe (EIFERT, 1992).

Baseado no "American Psychiatric Association, 1987", Eifert define que o cardiofóbico vivencia um tipo específico de ansiedade, a AC, porque o medo é persistente e fora de proporção frente ao real perigo, ou seja, os cardiofóbicos interpretam a precordialgia e/ou taquicardia de uma maneira catastrófica. Eles temem um ataque cardíaco ou doença cardíaca mesmo diante da certeza de que os sintomas vivenciados de precordialgia e/ou taquicardia não estão relacionados à uma doença cardíaca e/ou ataque cardíaco; o medo não pode ser explicado, ou seja, estas pessoas geralmente recebem garantias dos médicos e de outros profissionais de saúde que seu coração é organicamente saudável, mas persistem com sua crença de uma doença cardíaca; os estímulos temidos são evitados e o medo interfere na qualidade de vida dessa pessoa, pois os

pacientes acreditam que se deixarem de realizar determinados tipos de tarefas/atividades, eles podem prevenir um ataque cardíaco ou doença cardíaca, a ponto de deixarem de ser capazes de trabalhar. Eifert ainda afirma que a etiologia, os sintomas e o tratamento para a ansiedade cardíaca diferem dos da ansiedade (EIFERT, 1992).

A necessidade de realizar uma cirurgia cardíaca pode gerar estresse para o paciente, pois trata-se de um procedimento de alta complexidade (CAMPONOGARA et al., 2012).

No ano de 1946, Selye definiu estresse como um fator interno ou externo, imaginário ou real, que afeta o estado normal do equilíbrio dinâmico de uma pessoa (homeostasia) e pode ser físico ou psicológico (SELYE, 1946). Frente ao estresse, a pessoa pode classificá-lo como ameaça (estímulo negativo) ou desafio (estímulo positivo) e a estimulação e a resposta do organismo ocorrem devido à liberação de catecolaminas e corticosteroides (GONÇALVES; MEDEIROS, 2016). Essa resposta é conhecida como Síndrome da Adaptação Geral (SAG) e depende da intensidade, da qualidade e da quantidade dos estressores (SELYE, 1946). As fases da SAG são: alarme, resistência e exaustão (GONÇALVES; MEDEIROS, 2016).

A fase de alarme ocorre no momento de contato com o estressor e caracteriza-se por uma mobilização total das forças de defesa, que ocorre em frações de segundos e é dependente do estressor. Na fase de resistência, acontece a persistência do estressor e a tentativa de equilíbrio do organismo, podendo se adaptar ou eliminar o estressor. Já a fase de exaustão ocorre quando há uma falha no sistema de controle do estressor e manifesta-se com o surgimento de doenças relacionadas ao estresse, ou seja, pode ocorrer o comprometimento físico e psicológico decorrentes de controle malsucedido contra o estressor (SELYE, 1946).

Na SAG, durante a resposta ao estresse, duas vias podem ser utilizadas pelo hipotálamo, concomitantemente ou não: o eixo hipotálamo-hipófise-adrenal e a via do Sistema Nervoso Autônomo (SNA) (GUYTON; HALL, 2011).

A via hipotálamo-hipófise-córtex da adrenal é regulada pelas informações enviadas aos núcleos hipotalâmicos. Os principais fatores hipotalâmicos envolvidos são o fator de liberação da corticotropina, o hormônio de liberação do hormônio tireoestimulante, o fator de liberação de gonadotropinas, o hormônio de liberação ou inibição do hormônio do crescimento e o hormônio de inibição da prolactina (GUYTON, 2011). A neuro-hipófise sustenta feixes nervosos e fibras terminais provenientes do hipotálamo e essas terminações nervosas secretam os hormônios do lobo posterior da hipófise: a ocitocina e o hormônio antidiurético ou vasopressina (GUYTON; HALL, 2011).

O hipotálamo, diante de um estressor, estimula a liberação do hormônio adrenocorticotrófico pela hipófise, que atua no córtex adrenal, estimulando a secreção de

hormônios glicocorticoides e mineralocorticoides. O cortisol é o principal glicocorticoide e tem efeito na gliconeogênese realizada pelo fígado, elevando a glicose do sangue. A importância deste hormônio deve-se ao fato de que, em situações de estresse, ocorre rápida mobilização dos aminoácidos e líquidos das reservas celulares como fonte energética e síntese de substâncias para os diferentes tipos de tecidos orgânicos. O cortisol ainda promove a fadiga dos músculos esqueléticos, atua no sistema cardiovascular, aumentando a resposta vasomotora e o débito cardíaco, atua na produção de ácido clorídrico e do pepsinogênio no sistema gastrointestinal, dentre outras funções. Os mineralocorticoides têm como principal produto a aldosterona que atua nos eletrólitos dos líquidos extracelulares (potássio e sódio), no transporte de sódio e potássio, bem como no controle do volume de líquidos corporais (GUYTON; HALL, 2011).

As funções vegetativas do organismo são controladas pelo SNA e durante a fase de alarme existe a predominância da ação excitatória do SNA simpático, por intermédio de neurônios pós-ganglionares e pelas fibras simpáticas que secretam a adrenalina, provocando intenso metabolismo corporal e aumento da excitabilidade (GUYTON; HALL, 2011).

Diante de um estressor, a adrenalina e a noradrenalina secretadas produzem o aumento da broncodilatação, da frequência e da força da contração cardíaca, o aumento da contração dos vasos periféricos, o aumento da secreção gástrica, a redução ou o aumento do peristaltismo, o aumento do tônus esfincteriano, a diminuição do débito urinário, bem como o aumento da sudorese, do metabolismo basal, da atividade mental e da atividade musculoesquelética (GUYTON; HALL, 2011).

A exposição crônica ao estresse e consequentemente ao cortisol podem levar ao desgaste corporal e à respostas fisiológicas desreguladas, sendo capaz de causar mudanças nas regiões do cérebro que modulam a resposta ao estresse, o que pode contribuir com a fisiopatologia dos transtornos de humor, ansiedade e outras doenças (SELYE, 1946; SAPOLSKY; ROMERO; MUNK, 2000; MCEWEN, 2003, 2008; MCEWEN; GIANAROS, 2010).

Além disso, essa exposição crônica pode resultar no aparecimento de distúrbios do sono, sofrimento psicológico, diabetes por excesso de esteroides, degradação e inibição de síntese proteica nos músculos, tecido adiposo, linfoide, conjuntivo e adiposo, imunossupressão, alteração na liberação de hormônios ovarianos e ciclo menstrual, início precoce de doenças relacionadas com a idade e quadros típicos de ansiedade crônica e estresse pós-traumático, distúrbios psicóticos transitórios e mudanças no comportamento alimentar (MONTELEONE et al., 2005; ZORN et al., 2017; MAENG; MILAD, 2017; SEILER; FAGUNDES; CHRISTIAN, 2020; MILLER; CHEN; ZHOU, 2007; MORENO-PERAL et al., 2014; MCEWEN, 2004; RODENBECK et al., 2001; GLASER; KIECOLT-GLASER, 2005; MATHEVON et al., 2004;

KSIAZEK et al., 2007; CORTEZ; SILVA, 2007; VAN DER KOOY et al., 2007; STANSFELD et al., 2002; MYIN-GERMEYS et al., 2001; JONHSON, 1998; LUENG; MUNCK, 1975; MITRA et al., 2005).

As manifestações emocionais desencadeadas pelo estresse também são evidenciadas de maneira intensa sobre o coração, decorrentes da liberação dos corticosteroides e catecolaminas sobre o sistema cardiovascular, provocando aumento da frequência cardíaca e da pressão arterial sistêmica, do débito cardíaco, do consumo de oxigênio e excitabilidade cardíaca, aumento da adesividade plaquetária, lesão celular por entrada de sódio e saída de potássio e magnésio, retenção de sódio e água, vasoconstricção periférica, hemoconcentração, aumento da coagulação sanguínea, aumento dos ácidos e do colesterol e aumento da glicose e do ácido lático (RICE, 2012).

A resposta fisiológica ao estresse, envolvendo os sistemas endócrino e autonômico, pode influenciar a recuperação e reabilitação de pacientes submetidos à cirurgias cardíacas. O impacto do estresse pré-operatório no curso do PO pode aumentar o risco de desfechos indesejados, bem como pode aumentar o tempo de internação hospitalar (CUNHA, 2014).

Diante do exposto, acreditamos que os pacientes que serão submetidos a uma cirurgia cardíaca e que apresentarem altos níveis de ansiedade cardíaca poderão apresentar maior prejuízo no perioperatório, podendo interferir inclusive na adesão aos programas de reabilitação.

Assim, o reconhecimento precoce da ansiedade cardíaca pode favorecer o tratamento do paciente no perioperatório, diminuindo o nível de seu comprometimento funcional, aumentando a qualidade de vida e reduzindo custos dos serviços de saúde (EIFERT et al., 2000).

1.2.3 Instrumento de avaliação dos sintomas de ansiedade cardíaca

No ano 2000, foi criado um instrumento para a avaliação dos sintomas de AC que podia ser aplicado a pessoas com ou sem doença cardíaca. Em um primeiro momento, os autores criaram 63 itens, extraídos de três fontes, a saber: 16 itens de uma versão considerada um estudo piloto de avaliação de ansiedade cardíaca (EIFERT, 1992), entrevistas semiestruturadas realizadas com pacientes com cardiopatias em tratamento psicológico e revisão de literatura sobre a temática (EIFERT et al., 2000).

Os itens abordavam as principais características da ansiedade cardíaca, incluindo os medos e as preocupações das doenças cardíacas, o medo de precordialgia e outras sensações relacionadas ao coração, a atenção centrada no coração, a ajuda e a busca de confiança, e a

evitação de atividades que possam causar sintomas cardíacos. Para a obtenção da resposta em cada um dos itens, foi utilizada uma escala do tipo *Likert* de cinco pontos, que variava de zero (nunca) a quatro (sempre). O escore final era obtido com a soma das respostas dos itens, sendo que maiores valores indicavam maior presença de sintomas de ansiedade cardíaca (EIFERT et al., 2000).

A versão preliminar do instrumento foi aplicada em 188 pacientes. Desse total, 178 foram submetidos à Angioplastia Transluminal Percutânea (ATP) e dez pacientes com cardiopatias faziam acompanhamento psicológico no "*West Virginia University"*. Com esses resultados, os autores identificaram a estrutura fatorial e a consistência interna, e o instrumento passou a ter 18 itens. Em um segundo estudo, realizado com 30 pacientes submetidos à ATP e 12 pacientes com cardiopatias em acompanhamento psicológico, os autores realizaram a etapa de validade de constructo divergente e convergente e obtiveram como resultado um instrumento de 18 itens (EIFERT et al., 2000).

No ano de 2008, Sardinha e colaboradores (2008) realizaram a tradução e adaptação transcultural do Questionário de Ansiedade Cardíaca (QAC) para o Brasil, resultando desta pesquisa um questionário com 18 itens. No ano de 2013, Sardinha e colaboradores (2013) realizaram a etapa de validação do questionário, resultando em uma versão composta por 14 itens divididos em dois domínios: "Medo e hipervigilância de estímulos relacionados ao coração" (9 itens) e "Evitação de atividades que possam desencadear os sintomas" (5 itens). Dessa forma, diferindo da versão original, composta por 18 itens e três domínios. As respostas, como abordado anteriormente, são dadas por uma escala do tipo *Likert* de cinco pontos: nunca (0), raramente (1), às vezes (2), frequentemente (3) e sempre (4).

De acordo com os autores que realizaram a validação do QAC para o Brasil, a retirada dos quatro itens e a junção de dois domínios ("Medo" e "Atenção") foi necessária diante das respostas da avaliação psicométrica obtidas, mantendo-se o domínio "Evitação", ficando assim com 14 itens. Apesar disso, segundo os autores, os itens que compunham os domínios originais foram confirmados na versão brasileira (SARDINHA et al., 2013).

As questões que compõem o domínio "Medo e hipervigilância de estímulos relacionados ao coração" são: presto atenção nas batidas do meu coração; meu coração acelerado me acorda à noite; dor ou desconforto no peito me acordam à noite; mesmo que os exames estejam normais, eu continuo e preocupando com o meu coração; preocupa-me que os médicos não acreditem que meus sintomas sejam verdadeiros; quando tenho desconforto no peito ou meu coração está acelerado, preocupa-me que posso ter um ataque cardíaco; quando tenho desconforto no peito ou meu coração está acelerado, tenho dificuldade de me concentrar

em qualquer outra coisa; quando tenho desconforto no peito ou meu coração está acelerado, fico com medo e quando tenho desconforto no peito ou meu coração está acelerado, gosto de ser examinado por um médico. Já as questões que compõem o domínio "Evitação de atividades que possam desencadear os sintomas" são: evito esforço físico; pego leve o máximo possível; evito fazer exercícios ou outras atividades físicas; evito atividades que aceleram o meu coração e evito atividades que me façam suar (SARDINHA et al., 2013).

Os sintomas de ansiedade cardíaca podem desempenhar o papel de estressores adicionais, potencializando a resposta à SAG, o que pode prejudicar a recuperação e a reabilitação do paciente submetido à cirurgia cardíaca. Assim, a sua detecção precoce pode auxiliar a equipe multidisciplinar no planejamento psicoemocional desses pacientes.

1.2.4 Ansiedade cardíaca no perioperatório de cirurgias cardíacas

Na literatura, são escassos os artigos que investigaram a sintomatologia de AC no perioperatório de cirurgias cardíacas (HOYER et al., 2008; MORAES et al., 2013).

Na Alemanha, Hoyer e colaboradores (2008) realizaram a avalição dos sintomas de AC, de ansiedade, de depressão e da qualidade de vida de 90 pacientes submetidos às cirurgias de revascularização do miocárdio, de correção de valvopatia ou ambas. A coleta de dados foi realizada duas semanas antes da cirurgia cardíaca, seis semanas e seis meses após a cirurgia. Ao analisarem o escore total do questionário de avaliação dos sintomas de AC, os pacientes apresentaram menos sintomas de AC seis semanas e seis meses após a cirurgia cardíaca. Ao analisarem especificamente o escore do domínio "Medo e hipervigilância de estímulos relacionados ao coração", encontraram que estava significativamente reduzido seis semanas e seis meses após a cirurgia, assim como o escore total, entretanto, o escore do domínio "Evitação de atividades que possam desencadear os sintomas" não sofreu alterações significativas seis semanas após a cirurgia, mas apresentou redução significativa aos seis meses.

No Brasil, Moraes e colaboradores (2013) avaliaram o domínio "Medo e hipervigilância de estímulos relacionados ao coração" da AC de 91 pacientes que foram submetidos à cirurgia cardíaca há, no máximo, cinco anos, segundo o sexo e faixa etária (40 – 60 e 61 – 70), e não encontraram associação destas variáveis com AC.

Diante do exposto, a realização da presente investigação se justifica, já que além da escassez de investigações sobre sintomas de AC no perioperatório de cirurgias cardíacas, não encontramos estudos realizados com essa temática nos últimos cinco anos.

1.3 QUESTÕES DO ESTUDO E HIPÓTESES A SEREM TESTADAS

Considerando a proposta do estudo, de comparar os sintomas de ansiedade cardíaca de pacientes no pré e no pós-operatório de cirurgias cardíacas e de associar e correlacionar os sintomas com o sexo, a idade, a situação conjugal, o vínculo empregatício, o uso de medicamentos psicotrópicos antes da internação e os sintomas de ansiedade e depressão, pretendemos responder às seguintes questões:

Questão 1: Pacientes submetidos às cirurgias de revascularização do miocárdio e de correção de valvopatias apresentarão diferenças na presença de sintomas de ansiedade cardíaca, avaliados pelo Questionário de Ansiedade Cardíaca, no pré-operatório, no dia da alta hospitalar e no primeiro retorno após a alta hospitalar?

De acordo com a revisão da literatura e nossa experiência clínica, estabelecemos a hipótese verdadeira (H1):

H1: Pacientes submetidos às cirurgias de revascularização do miocárdio e de correção de valvopatias apresentarão diferenças na presença de sintomas de ansiedade cardíaca, avaliados pelo Questionário de Ansiedade Cardíaca, no pré-operatório, no dia da alta hospitalar e no primeiro retorno após a alta hospitalar.

Questão 2: Existe associação dos sintomas de ansiedade cardíaca, avaliados pelo Questionário de Ansiedade Cardíaca, com o sexo, a idade, a situação conjugal e o vínculo empregatício dos pacientes submetidos às cirurgias de revascularização do miocárdio e de correção de valvopatias no pré-operatório e no primeiro retorno após a alta hospitalar?

De acordo com a revisão da literatura e nossa experiência clínica, estabelecemos a hipótese verdadeira (H1):

H1: Haverá associação dos sintomas de ansiedade cardíaca com o sexo, a idade, o estado civil e o vínculo empregatício dos pacientes submetidos às cirurgias de revascularização do miocárdio e de correção de valvopatias no pré-operatório e no primeiro retorno após a alta hospitalar.

Questão 3: Pacientes submetidos às cirurgias de revascularização do miocárdio e de correção de valvopatias que fazem uso de medicamentos psicotrópicos em casa apresentarão diferenças na presença de sintomas de ansiedade cardíaca, avaliados pelo Questionário de Ansiedade

Cardíaca, no pré-operatório, quando comparados com pacientes que não fazem uso desses medicamentos?

De acordo com a revisão da literatura e nossa experiência clínica, estabelecemos a hipótese verdadeira (H1):

H1: Pacientes submetidos às cirurgias de revascularização do miocárdio e de correção de valvopatias que fazem uso de psicotrópicos em casa apresentarão diferenças na presença de sintomas de ansiedade cardíaca, avaliados pelo Questionário de Ansiedade Cardíaca, no pré-operatório, quando comparados com pacientes que não fazem uso desses medicamentos.

Questão 4: Existe correlação dos sintomas de ansiedade cardíaca, avaliados pelo Questionário de Ansiedade Cardíaca, com os sintomas de ansiedade e depressão, avaliados pelo *Hospital Anxiety and Depression Scale* (HADS), dos pacientes submetidos às cirurgias de revascularização do miocárdio e de correção de valvopatias no pré-operatório, no dia da alta hospitalar e no primeiro retorno após a alta hospitalar?

De acordo com a revisão da literatura e nossa experiência clínica, estabelecemos a hipótese verdadeira (H1):

H1: Haverá correlação dos sintomas de ansiedade cardíaca, avaliados pelo Questionário de Ansiedade Cardíaca, com os sintomas de ansiedade e depressão, avaliados pelo Hospital Anxiety and Depression Scale, dos pacientes submetidos às cirurgias de revascularização do miocárdio e de correção de valvopatias no pré-operatório, no dia da alta hospitalar e no primeiro retorno após a alta hospitalar.

2. OBJETIVOS

- Comparar os sintomas de ansiedade cardíaca em pacientes submetidos às cirurgias de revascularização do miocárdio e de correção de valvopatias no pré-operatório, no dia da alta hospitalar e no primeiro retorno após a alta hospitalar.

- Investigar a associação dos sintomas de ansiedade cardíaca com o sexo, a idade, a situação conjugal e o vínculo empregatício em pacientes submetidos às cirurgias de revascularização do miocárdio e de correção de valvopatias no pré-operatório e no primeiro retorno após a alta hospitalar.

- Investigar a associação dos sintomas de ansiedade cardíaca com uso de medicamentos psicotrópicos em casa em pacientes submetidos às cirurgias de revascularização do miocárdio e de correção de valvopatias, no pré-operatório.

- Investigar a correlação dos sintomas de ansiedade cardíaca com os sintomas de ansiedade e depressão em pacientes submetidos às cirurgias de revascularização do miocárdio e de correção de valvopatias no pré-operatório, no dia da alta hospitalar e no primeiro retorno após a alta hospitalar.

3. MÉTODO

3.1 DELINEAMENTO DO ESTUDO

Trata-se de um estudo observacional analítico de coorte prospectiva.

Os estudos observacionais analíticos têm como objetivo caracterizar as associações entre as variáveis de interesse. Na pesquisa clínica, coorte é um grupo de sujeitos especificados no início do estudo e seguidos no tempo. Na coorte prospectiva, seleciona-se uma amostra de sujeitos e, então, medem-se, em cada sujeito, características que poderão predizer desfechos subsequentes. Então, esses sujeitos são seguidos no tempo por meio de medições periódicas dos desfechos de interesse (HULLEY; CUMMINGS; NEWMAN, 2015).

3.2 LOCAL E CASUÍSTICA

O estudo foi realizado nas Unidades de Internação de Clínica Cirúrgica e no Ambulatório de Cirurgia Cardíaca de um hospital universitário do interior paulista no período de fevereiro de 2018 a agosto de 2019.

Uma amostra consecutiva e não probabilística foi constituída pelos pacientes que atenderam aos critérios de inclusão: ambos os sexos, com idade acima de 18 anos, que foram submetidos à CRM ou à cirurgias para correção de valvopatias (plastia e/ou troca), independentemente de ser primeira cirurgia ou reoperação, e que tiveram o agendamento eletivo de suas cirurgias com mais de 12 horas de antecedência.

Foram excluídos os pacientes que não apresentaram condições cognitivas para responder aos questionários no dia da coleta de dados no pré-operatório (primeira avaliação) e/ou que apresentaram descompensação clínica da doença cardíaca na primeira fase da coleta de dados (presença de dispneia, precordialgia ou entubação orotraqueal)

Foram descontinuados os pacientes que foram submetidos à uma segunda abordagem cirúrgica durante o tempo de internação e/ou que reinternaram antes do primeiro retorno após a alta hospitalar.

Para identificarmos os pacientes que possuíam condições cognitivas para responder aos questionários, foram utilizadas seis questões, sendo quatro adaptadas do instrumento elaborado por Pfeiffer (1975) que compreendem: “Qual a data de hoje?”, “Qual a sua idade?”, “Em que dia da semana estamos?”, “Qual o nome do local que estamos nesse momento?”; e duas elaboradas por Silva (2016), que compreendem: “Qual o seu nome completo?” e “Qual o nome

da cidade em que você nasceu?". Os participantes foram excluídos do estudo se erraram ou não souberem informar três ou mais questões.

Optamos pelo uso da amostra consecutiva e não probabilística devido à falta de dados sobre a variância da variável desfecho na população-alvo, a saber: sintomas de AC, o que impossibilita o cálculo do tamanho amostral. Sendo assim, as hipóteses elaboradas no estudo são hipóteses exploratórias, e não confirmatórias.

3.3 ASPECTOS ÉTICOS

O projeto de pesquisa foi elaborado de acordo com os preceitos éticos da Resolução do Conselho Nacional de Saúde n.466 de 12 dezembro de 2012 e encontra-se aprovado pelo Comitê de Ética em Pesquisa da Escola de Enfermagem de Ribeirão Preto da Universidade de São Paulo, número de Certificação de Apresentação e Apreciação Ética (CAAE): 75120717.9.0000.5393 (ANEXO A). Os pacientes foram convidados a participar da pesquisa e, após concordarem, foram realizadas a leitura e a assinatura do Termo de Consentimento Livre e Esclarecido (TCLE) (APÊNDICE A) e fornecida, em duas vias, uma via para o paciente e a outra será arquivada pelo pesquisador.

3.4 PROCEDIMENTOS PARA COLETA DE DADOS

A coleta de dados foi realizada por meio de entrevistas individuais e consulta aos prontuários dos participantes, em três momentos:

(T_0): no período pré-operatório, nas Unidades de Internação da Clínica Cirúrgica, no dia que antecedeu a cirurgia, foram coletados os dados para a caracterização sociodemográfica e clínica, por meio de entrevista com os pacientes e consulta aos prontuários, além da avaliação dos sintomas de ansiedade cardíaca, ansiedade e depressão por meio de entrevista.

(T_1): no pós-operatório, no dia da alta hospitalar, os sintomas de ansiedade cardíaca, ansiedade e depressão foram novamente avaliados por meio de entrevista.

(T_2): no dia do primeiro retorno após a alta hospitalar, que ocorre 14 dias (± 7 dias) após alta hospitalar, os sintomas de ansiedade cardíaca, ansiedade e depressão foram novamente avaliados, por meio de entrevista, no Ambulatório de Cirurgia Cardíaca (Figura 1).

Figura 1 - Diagrama da coleta de dados

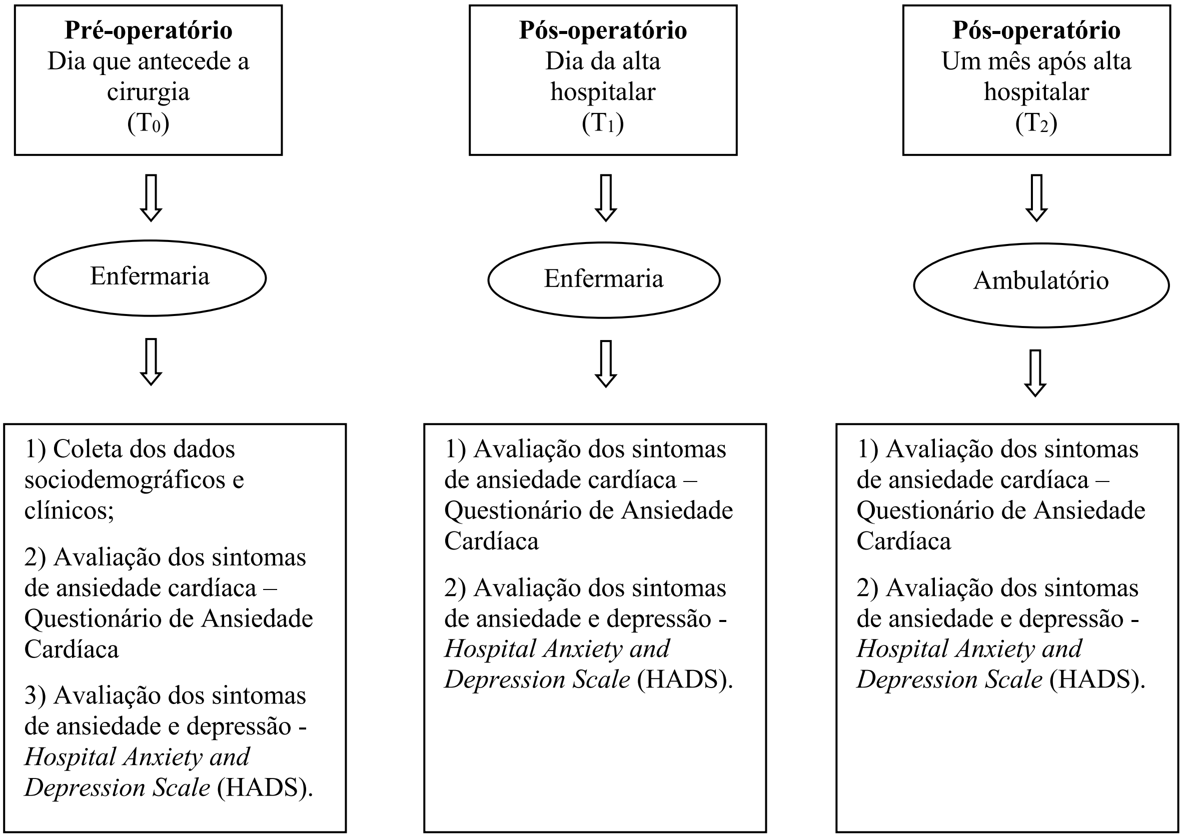

Fonte: autora, 2020.

3.5 LINHA DO TEMPO REFERENTE AOS TRÊS MOMENTOS DE COLETA DE DADOS

A internação do paciente na Unidade de Internação de Clínica Cirúrgica do referido hospital ocorre, em média, três dias antes da abordagem cirúrgica para a realização dos exames pré-operatórios necessários. Após a abordagem cirúrgica, o paciente permanece, em média, 72 horas na Unidade de Terapia Intensiva (UTI). Após a alta da UTI e retorno para a enfermaria, o paciente permanece, em média, quatro dias internado para a realização dos exames pós-operatórios, que são necessários para a alta hospitalar. O primeiro retorno no hospital, após a alta hospitalar, ocorre 14 dias após a alta, às sextas-feiras, no Ambulatório de Cirurgia Cardíaca, com possível antecipação de sete dias caso o paciente tenha apresentado intercorrências durante a internação, e atraso de sete dias, caso o retorno coincida com feriados (Figura 2).

Figura 2 - Linha do tempo referente a coleta de dados

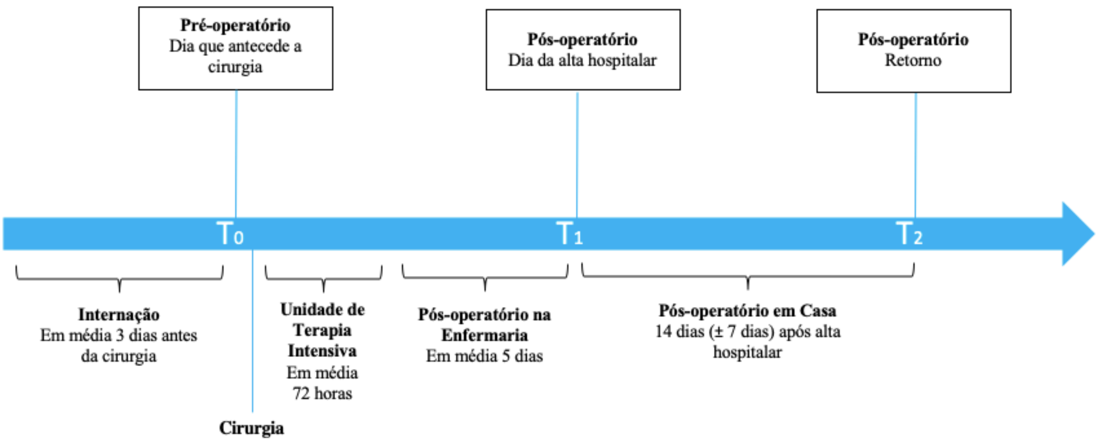

Fonte: autora, 2020

3.6 INSTRUMENTOS DE COLETA DE DADOS

3.6.1 Instrumento de caracterização sociodemográfica e clínica

Para a caracterização sociodemográfica e clínica dos participantes, foi elaborado um instrumento, com base na revisão da literatura e em estudos anteriores, contendo os dados (APÊNDICE B):

Sociodemográficos: datas de nascimento, de internação e da entrevista, sexo, presença de companheiro, escolaridade, situação profissional, renda mensal familiar e número de pessoas que dependem da renda. A idade foi calculada subtraindo a data da entrevista da data de nascimento.

Clínicos: diagnóstico principal (DAC ou valvopatias), presença de patologias associadas, hábitos de vida (tabagismo), utilização prévia de psicotrópicos no domicílio, remarcação da cirurgia e motivo (quando ela foi necessária), data da cirurgia, cirurgia realizada, tempo de internação pré-operatório, tempo de internação na UTI, tempo de internação na enfermaria pós-alta da UTI, tempo de internação geral e tempo do primeiro retorno.

O tempo de internação pré-operatório foi calculado subtraindo a data da cirurgia da data de internação, já o tempo de internação na UTI foi calculado subtraindo a data da alta da UTI da data da cirurgia. Para o cálculo do tempo de internação no PO na enfermaria, subtraímos a

data da alta hospitalar da data da alta da UTI. O cálculo do tempo total de internação foi encontrado ao subtrairmos a data da alta hospitalar da data da internação. Por fim, o tempo entre a alta hospitalar e o primeiro retorno foi calculado subtraindo a data do retorno da data da alta hospitalar.

O uso de psicofármacos foi investigado por estarmos avaliando, por meio de instrumentos psicométricos, constructos subjetivos. Tais avaliações podem ser comprometidas pelo uso de psicofármacos do tipo antidepressivos, ansiolíticos e estabilizadores do humor (como os anticonvulsivantes) (LARAIA, 2001).

Investigamos a utilização prévia de psicotrópicos no domicílio, anteriormente à internação, pois identificamos em estudos anteriores que a utilização destes medicamentos durante a internação são, em sua maioria, indutores de sono (KAZITANI et al., 2018; RODRIGUES et al., 2018). Assim, consideramos a utilização durante a internação como uma situação pontual que se caracteriza distintamente do uso clínico prévio ao tratamento cirúrgico.

3.6.2 Instrumento para avaliação dos sintomas de ansiedade cardíaca

Para a avaliação dos sintomas de ansiedade cardíaca, foi utilizado o "Questionário de Ansiedade Cardíaca" (EIFERT et al., 2000), em sua versão validada para o português (SARDINHA et al., 2013) (ANEXO B).

O "Questionário de Ansiedade Cardíaca" (QAC) é composto por 14 itens avaliados por meio de Escala tipo *Likert* de cinco pontos: (0) nunca, (1) raramente, (2) às vezes, (3) frequentemente e (4) sempre. Esse questionário possui dois domínios: "Medo e hipervigilância de estímulos relacionados ao coração" (itens 1, 3, 4, 8, 10, 11, 12, 13 e 14) e "Evitação de atividades que possam desencadear os sintomas" (itens 2, 5, 6, 7 e 9). O escore total é obtido por meio da soma das respostas aos 14 itens, sendo possível uma variação de 0-56, com maiores valores indicando maior percepção de ansiedade cardíaca pelo paciente. Também é possível obter os escores dos dois domínios, sendo assim, o domínio "Medo e hipervigilância de estímulos relacionados ao coração" (9 itens) poderá apresentar uma variação de 0-36 e o domínio "Evitação de atividades que possam desencadear os sintomas" (5 itens) uma variação de 0-20, ambos com maiores valores indicando maior percepção de ansiedade cardíaca pelo paciente.

3.6.3 Instrumento para avaliação dos sintomas de ansiedade e depressão

Para a avaliação dos sintomas de ansiedade e depressão, foi utilizado o instrumento *Hospital Anxiety and Depression Scale* (HADS) (ZIGMOND; SNAITH, 1983) validado para o português (BOTEGA et al., 1995) (ANEXO C).

O instrumento HADS possui 14 questões (sete para cada sintoma), que abordam sintomas psicológicos e somáticos. A resposta se dá por meio de uma escala de quatro pontos, cujos valores variam de zero a três, assim, a soma pode variar de zero a 21 (vinte e um) pontos para cada um dos transtornos emocionais pesquisados.

No presente estudo, a avaliação das respostas foi feita com o valor total de cada subescala (HADS-ansiedade e HADS-depressão), sendo que valores maiores estão associados à maior percepção dos sintomas de ansiedade e depressão.

3.7 PROCESSAMENTO E ANÁLISE DOS DADOS

Os dados foram inseridos no programa Office Excel 2010 com a técnica da dupla digitação, seguida da validação. Após a validação, os dados foram transferidos para o Programa IBM-SPSS, versão 22.0 para Windows (SPSS, Inc., Chicago, IL, USA).

Para a caracterização sociodemográfica e clínica, foram realizadas análises descritivas de frequência simples para variáveis nominais ou categóricas e análise de tendência central (média e mediana) e dispersão (desvio-padrão) para as variáveis numéricas.

Para a comparação dos sintomas de ansiedade cardíaca de pacientes no pré-operatório, no dia da alta hospitalar e no primeiro retorno após a alta hospitalar, foi utilizado o teste de Friedman.

Para a investigação da associação dos sintomas de ansiedade cardíaca com o sexo (masculino ou feminino), a idade (adulto ou idoso), a presença de companheiro (sim ou não), o desempenho de atividades remuneradas (sim ou não) no pré-operatório e no primeiro retorno após a alta hospitalar, foi utilizado o teste de Mann-Whitney.

Para a investigação da associação dos sintomas de ansiedade cardíaca com o uso de psicotrópicos em casa (sim ou não) no pré-operatório, foi utilizado o teste de Mann-Whitney.

Para a investigação da correlação dos sintomas de ansiedade cardíaca com os sintomas de ansiedade e depressão no pré-operatório (T_0), no dia da alta hospitalar (T_1) e no primeiro retorno após a alta hospitalar (T_2), foi utilizado o teste de Correlação de Spearman.

Para análise das forças de correlação linear entre as medidas, foi utilizada a classificação proposta por Ajzen e Fishbein (1998), que determina que valores de correlação menores do que 0,30, mesmo quando estatisticamente significantes, não apresentam relevância clínica, valores entre 0,30 – 0,50 indicam moderada correlação e acima de 0,50 apresentam correlação forte.

O nível de significância adotado foi de 0,05.

4. RESULTADOS

No período de coleta de dados, de fevereiro de 2018 a agosto de 2019, foram realizadas 145 cirurgias cardíacas, sendo que 79 foram correções de valvopatias e 60 CRM. Desse total, seis pacientes não atenderam aos critérios de inclusão. Com relação aos pacientes submetidos às CRM, oito não aceitaram participar do estudo, 20 foram considerados perdas da coleta de dados, três vieram à óbito e três descontinuados do estudo. Quanto aos pacientes submetidos às cirurgias de correções de valvopatias, três não aceitaram participar do estudo, sete foram considerados perdas da coleta de dados, seis vieram à óbito e cinco foram descontinuados do estudo. Assim, ao final, a amostra foi constituída por 34 (36,9%) coronariopatas submetidos à CRM e 58 (63,1%) pacientes valvopatas submetidos à correção cirúrgica de valvopatia, totalizando 92 participantes. A seguir um fluxograma dos participantes do estudo será apresentado (figura 3).

Figura 3 - Fluxograma dos participantes do estudo

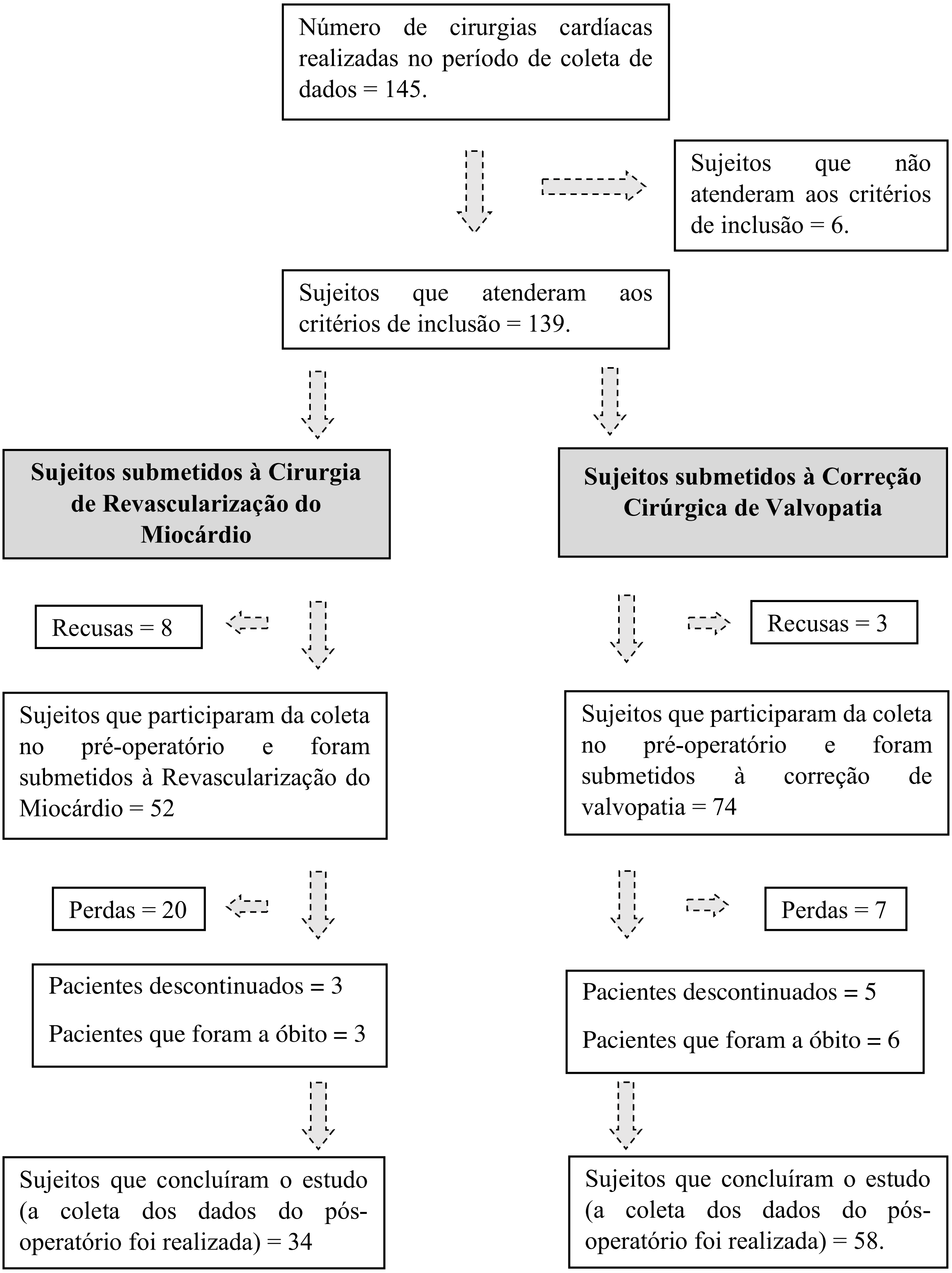

Fonte: autora, 2020

Na tabela 1 encontra-se o detalhamento dos motivos das perdas da coleta de dados, do momento do óbito e da descontinuação dos pacientes, segundo o tipo de cirurgia.

Tabela 1 - Relação dos motivos das perdas da coleta de dados, do momento do óbito e da descontinuação dos pacientes submetidos à cirurgia de revascularização do miocárdio e cirurgia para correção de valvopatia. Ribeirão Preto, 2018-2019.

	**Revascularização do Miocárdio n (%)**	**Correção de valvopatias n (%)**
**Perdas**		
Não comparecimento no retorno ambulatorial	2 (10%)	1 (14,2%)
Não encontrado no retorno ambulatorial	3 (15%)	1 (14,2%)
Alta hospitalar sem aviso	6 (30%)	2 (28,5%)
Mudança na escala cirúrgica	9 (45%)	3 (42,8%)
**Óbitos**		
Pré-operatório		1 (16,6%)
Intraoperatório		1 (16,6%)
Pós-operatório		
Imediato		2 (33,3%)
Mediato	3 (100%)	2 (33,3%)
**Descontinuação**		
Reabordagem cirúrgica	1 (33,3%)	3 (60%)
Reinternação logo após a alta hospitalar	2 (66.6%)	2 (40%)

Fonte: autora, 2020

4.1 CARACTERIZAÇÃO SOCIODEMOGRÁFICA E CLÍNICA DOS PACIENTES NO PRÉ-OPERATÓRIO

A caracterização sociodemográfica e clínica dos 34 pacientes que foram submetidos à CRM encontra-se nas tabelas 2 e 3. A Tabela 2 apresenta a caracterização sociodemográfica dos pacientes segundo o sexo, a idade, a situação conjugal, a escolaridade, a renda mensal, o número de pessoas que dependem da renda e a situação profissional.

Tabela 2 - Caracterização sociodemográfica dos 34 pacientes submetidos à cirurgia de revascularização do miocárdio, segundo sexo, idade, situação conjugal, escolaridade, renda mensal, número de pessoas que dependem da renda e situação profissional. Ribeirão Preto, 2018-2019.

Variável		n (%)	Média (DP)*	Mediana
**Sexo**				
	Masculino	22 (64,7)		
	Feminino	12 (35,7)		
**Idade**			62,4 (9,8)	61,5
**Situação conjugal**				
	Com companheiro	23 (67,6)		
	Sem companheiro	11 (32,4)		
**Escolaridade (anos completos)**			7,2 (4,0)	6,5
**Renda mensal familiar (em reais)**			2.936,2 (2.619,3)	2.390,0
**Pessoas Dependentes da Renda**			2,7 (1,4)	2,0
**Situação Profissional**				
	Inativo	20 (58,8)		
	Ativo	14 (41,2)		

Média (DP)* = Média (Desvio-Padrão).
Fonte: autora, 2020.

Observa-se que a maioria dos pacientes submetida à CRM era do sexo masculino, vivia com companheiro e estava inativa na internação. A média de idade foi de 62,4 anos, configurando pacientes mais idosos. Os pacientes apresentaram baixa escolaridade e renda mensal.

Na tabela 3 encontra-se a caracterização clínica dos 34 participantes submetidos à CRM, de acordo com as doenças associadas e hábitos de vida.

Tabela 3 - Caracterização clínica dos 34 pacientes submetidos à cirurgia de revascularização do miocárdio, segundo a presença de doenças associadas e hábitos de vida. Ribeirão Preto, 2018 – 2019.

Variável	n (%)
**Presença de doenças associadas**	
Hipertensão arterial sistêmica	33 (97,1)
Sobrepeso/obesidade	27 (79,4)
Dislipidemia	24 (70,6)
Diabetes mellitus	20 (58,8)
Hipotireoidismo	4 (11,8)
**Hábitos de Vida**	
Tabagismo	
Pregresso	18 (52,9)
Ativo	4 (11,8)

Fonte: autora, 2020

Observa-se que a maioria dos pacientes submetida à CRM já apresentava hipertensão arterial sistêmica, sobrepeso/obesidade, dislipidemia e diabetes mellitus na internação e 52,9% relataram tabagismo pregresso.

A caracterização sociodemográfica e clínica dos 58 pacientes que foram submetidos à cirurgia de correção de valvopatias encontra-se nas Tabelas 4 e 5. A Tabela 4 apresenta a caracterização sociodemográfica dos 58 pacientes segundo o sexo, a idade, a situação conjugal, a escolaridade, a renda mensal, o número de pessoas que dependem da renda e a situação profissional.

Tabela 4 - Caracterização sociodemográfica dos 58 pacientes submetidos a cirurgia para correção de valvopatia segundo sexo, idade, situação conjugal, escolaridade, renda mensal, número de pessoas que dependem da renda e situação profissional. Ribeirão Preto, 2018-2019.

Variável		n (%)	Média (DP)*	Mediana
**Sexo**				
	Feminino	30 (51,7)		
	Masculino	28 (48,3)		
**Idade**			54,7 (14,1)	57,2
**Situação conjugal**				
	Com companheiro	41 (70,7)		
	Sem companheiro	17 (29,3)		
**Escolaridade (anos completos)**			6,4 (4,9)	4,5
**Renda Mensal (em reais)**			2.450,93 (1.624,7)	2.000,0
**Pessoas Dependentes da Renda**			2,3 (1,2)	2,0
**Situação Profissional**				
	Inativo	35 (60,3)		
	Ativo	23 (39,7)		

Média (DP)* = Média (Desvio-Padrão); Renda mensal familiar (reais).
Fonte: autora, 2020

Observa-se que a maioria dos pacientes submetida à correção cirúrgica de valvopatias era do sexo feminino, vivia com companheiro e estava inativa na internação. A média de idade foi de 54,7 anos. Os pacientes apresentaram baixa escolaridade e renda mensal.

Na tabela 5 encontra-se a caracterização clínica dos 58 participantes submetidos à correção cirúrgica de valvopatias, de acordo com as doenças associadas e hábitos de vida.

Tabela 5 - Caracterização clínica dos 58 pacientes submetidos à cirurgia para correção de valvopatia segundo a presença de doenças associadas e hábitos de vida. Ribeirão Preto, 2018 – 2019.

Variável	n (%)
**Presença de doenças associadas**	
Hipertensão arterial sistêmica	42 (72,4)
Sobrepeso/obesidade	39 (67,2)
Dislipidemia	25 (41,3)
Hipotireoidismo	9 (15,5)
Diabetes mellitus	8 (13,8)
**Hábitos de Vida**	
Tabagismo	
Pregresso	24 (41,4)
Ativo	6 (10,3)

Fonte: autora, 2020

Observa-se que a maioria dos pacientes submetida à correção cirúrgica de valvopatias já apresentava hipertensão arterial sistêmica e sobrepeso/obesidade na internação e 41,4% relataram tabagismo pregresso.

Com relação ao uso de psicotrópicos dos pacientes submetidos à CRM, do total da amostra, sete pacientes (20,6%) faziam uso em casa. Quanto à suspensão da cirurgia de CRM, nove pacientes (26,5%) tiveram suas cirurgias suspensas e remarcadas pelo menos uma vez, e três pacientes (8,8%) tiveram suas cirurgias suspensas e remarcadas mais de duas vezes. Os motivos da suspensão foram: falta de material (n=2; 11,1%), queda de energia no centro cirúrgico (n=3; 16,6%), urgências (n=7; 38,8%) e intercorrências com o paciente (n=6; 33,3%).

Já os pacientes submetidos às cirurgias para correção de valvopatias, do total da amostra, 22 pacientes (37,9%) faziam uso de psicotrópicos em casa. Quanto à suspensão da cirurgia, 11 pacientes (19,0%) tiveram suas cirurgias suspensas e remarcadas pelo menos uma vez, três pacientes (5,2%) tiveram suas cirurgias suspensas e remarcadas duas vezes e três pacientes tiveram suas cirurgias suspensas e remarcadas mais de duas vezes (5,2%). Os motivos da suspensão foram: falta de material (n=3; 11,1 %), queda de energia no centro cirúrgico (n=4; 14,8%), urgências (n=12; 44,4%) e intercorrências com o paciente (n=8; 29,6%).

Quanto à presença de dor precordial, apenas 11 pacientes (32,4%) submetidos à CRM relataram esse sintoma, ao passo que metade dos pacientes submetidos às cirurgias de correção de valvopatias (n=29; 50%) relataram esse sintoma.

Nas tabelas 6 e 7 encontram-se os tempos de internação e tempo do primeiro retorno, em dias, dos pacientes submetidos à CRM e pacientes submetidos à correção cirúrgica de valvopatias, respectivamente.

Tabela 6 - Tempo de internação em dias no pré-operatório, no pós-operatório imediato, no pós-operatório mediato, na internação total e tempo do primeiro retorno dos 34 pacientes submetidos à cirurgia de revascularização do miocárdio. Ribeirão Preto, 2018-2019.

	**Tempo de Internação**				
	**Pré-Operatório**	**Pós-Operatório Imediato**	**Pós-Operatório Mediato**	**Total**	**Primeiro Retorno**
**Média (DP)***	8,5 (8,7)	4,7 (2,5)	5 (2,4)	18,2 (8,7)	13,8 (5,9)
**Mediana**	4	4	5	16	14
**Mínimo**	1	2	1	7	5
**Máximo**	33	12	11	39	29

(DP)*= Desvio-Padrão
Fonte: autora, 2020

Tabela 7 - Tempo de internação em dias no pré-operatório, no pós-operatório imediato, no pós-operatório mediato, na internação total e tempo do primeiro retorno dos 58 pacientes submetidos à cirurgia para correção de valvopatia. Ribeirão Preto, 2018-2019.

	**Tempo de Internação**				
	**Pré-Operatório**	**Pós-Operatório Imediato**	**Pós-Operatório Mediato**	**Total**	**Primeiro Retorno**
**Média (DP)***	6,5 (7,3)	5,3 (5,3)	8,3 (5,2)	20,2 (13,6)	14,8 (4,6)
**Mediana**	3	4	7	15,5	15
**Mínimo**	1	2	1	7	4
**Máximo**	32	40	29	78	24

(DP)*= Desvio-Padrão
Fonte: autora, 2020

Já de acordo com o número de cirurgias, apenas dois pacientes (5,9%) do grupo de CRM foram submetidos à uma reoperação, ao passo no grupo de correção de valvopatias, 10 pacientes (17,2%) foram submetidos à reoperação.

4.2 COMPARAÇÃO DOS SINTOMAS DE ANSIEDADE CARDÍACA NO PRÉ-OPERATÓRIO, NO DIA DA ALTA E NO PRIMEIRO RETORNO APÓS ALTA HOSPITALAR DOS PACIENTES

Na tabela 8 encontram-se as medianas das medidas de ansiedade cardíaca e sua comparação nos três tempos propostos nesse estudo, pré-operatório, dia da alta hospitalar e no primeiro retorno após alta hospitalar dos 34 pacientes submetidos à CRM.

Tabela 8 - Comparação das medianas dos sintomas de ansiedade cardíaca dos 34 pacientes submetidos à cirurgia de revascularização do miocárdio no pré-operatório, dia da alta e no primeiro retorno após alta hospitalar e os valores de probabilidade (*p*) associados ao teste de Friedman. Ribeirão Preto, 2018-2019.

**Variável**	**Pré-Operatório** Mediana (Mín-Máx)*	**Alta** Mediana (Mín-Máx)*	**Primeiro Retorno** Mediana (Mín-Máx)*
Ansiedade Cardíaca	34,0 (4 – 50)	38,0 (25 – 54)	38,5 (18 – 48)
	p** **= 0,008**		
**Domínios QAC*****			
Medo e Hipervigilância	18,5 (2 – 30)	19,0 (6 – 34)	18 (5 – 32)
	p = 0,154		
Evitação	16,0 (0 – 20)	20,0 (8 – 20)	20,0 (2 – 20)
	p** **= 0,001**		

* Mín-Máx: Mínimo-Máximo; ** $p<0,05$: significância estatística; *** Domínios QAC: Domínios do Questionário de Ansiedade Cardíaca.
Fonte: autora, 2020

Na tabela 9 encontram-se os valores do qui-quadrado e graus de liberdade do teste de Friedman referentes à tabela 8.

Tabela 9- Descrição dos valores do qui-quadrado e graus de liberdade do teste de Friedman referente aos 34 pacientes submetidos à cirurgia de revascularização do miocárdio. Ribeirão Preto, 2018-2019.

	**Ansiedade Cardíaca**	**Medo e Hipervigilância**	**Evitação**
Qui-quadrado	9,8	3,7	13,8
Graus de liberdade	2	2	2

Fonte: autora, 2020

Podemos observar o efeito do tempo nos sintomas de ansiedade cardíaca dos pacientes submetidos à CRM tanto no escore total quanto no domínio "Evitação". O teste de comparações múltiplas mostrou que os valores dos postos dos sintomas de ansiedade cardíaca total no pré-operatório e no dia da alta foram diferentes com significância estatística (p=0,027), bem como no pré-operatório e no primeiro retorno hospitalar (p=0,023), ou seja, os pacientes apresentaram mais sintomas de AC no dia da alta quando comparados com o pré-operatório, assim como apresentaram mais sintomas no dia do primeiro retorno comparados com o pré-operatório.

Quanto ao domínio "Evitação", os valores dos postos encontrados no pré-operatório e na alta, assim como no pré-operatório e retorno, foram diferentes com significância estatística, p=0,027 e p=0,039, respectivamente. Assim, os pacientes apresentaram mais sintomatologia da AC no dia da alta hospitalar e no primeiro retorno quando comparados com o pré-operatório.

Na tabela 10 encontram-se as medianas das medidas de ansiedade cardíaca e sua comparação nos três tempos propostos nesse estudo, pré-operatório, dia da alta hospitalar e no primeiro retorno após alta hospitalar, dos 58 pacientes submetidos à correção cirúrgica de valvopatias.

Tabela 10 - Comparação das medianas dos sintomas de ansiedade cardíaca dos 58 pacientes submetidos à cirurgia para correção de valvopatia no pré-operatório, dia da alta e no primeiro retorno após alta hospitalar e os valores de probabilidade (*p*) associados ao teste de Friedman. Ribeirão Preto, 2018-2019.

**Variável**	**Pré-Operatório** Mediana (Mín-Máx)*	**Alta** Mediana (Mín-Máx)*	**Primeiro Retorno** Mediana (Mín-Máx)*
Ansiedade Cardíaca	36,0 (7 – 55)	39,0 (16 – 50)	38,5 (13 – 56)
	p **** = 0,040**		
**Domínios QAC*****			
Medo e Hipervigilância	21,5 (2 – 35)	20,0 (4 – 31)	19,5 (5 – 36)
	p = 0,687		
Evitação	16,0 (0 – 20)	20,0 (0 – 20)	20,0 (2 – 20)
	p **** = 0,002**		

* Min-Máx: Mínimo-Máximo; ** *p*<0,05: significância estatística; *** Domínios QAC: Domínios do Questionário de Ansiedade Cardíaca.
Fonte: autora, 2020

Na tabela 11 encontram-se os valores do qui-quadrado e graus de liberdade do teste de Friedman referentes à tabela 10.

Tabela 11 - Descrição dos valores do qui-quadrado e graus de liberdade do teste de Friedman referente aos 58 pacientes submetidos à cirurgia para correção de valvopatia. Ribeirão Preto, 2018-2019.

	**Ansiedade Cardíaca**	**Medo e Hipervigilância**	**Evitação**
Qui-quadrado	6,4	0,7	12,3
Graus de liberdade	2	2	2

Fonte: autora, 2020

Podemos observar o efeito do tempo nos sintomas de ansiedade cardíaca dos pacientes submetidos à correção cirúrgica de valvopatias tanto no escore total quanto no domínio "Evitação". O teste de comparações múltiplas mostrou que os valores dos postos dos sintomas de ansiedade cardíaca total no pré-operatório e no primeiro retorno hospitalar foram diferentes com significância estatística (*p*=0,042). Os pacientes apresentaram mais sintomas de AC no primeiro retorno quando comparados com a sintomatologia no pré-operatório.

Quanto ao domínio “Evitação”, os valores dos postos encontrados no pré-operatório e no primeiro retorno hospitalar foram diferentes com significância estatística (*p*=0,021). Assim, os pacientes apresentaram mais sintomatologia da AC no dia do primeiro retorno quando comparados com o pré-operatório.

4.3 RELAÇÃO DOS SINTOMAS DE ANSIEDADE CARDÍACA NO PRÉ-OPERATÓRIO E NO PRIMEIRO RETORNO HOSPITALAR COM AS CARACTERÍSTICAS SOCIODEMOGRÁFICAS E CLÍNICAS DOS PACIENTES

Na tabela 12, encontram-se as medianas e os valores mínimos e máximos dos sintomas de ansiedade cardíaca no pré-operatório, segundo o sexo, a idade, a situação conjugal, a situação profissional e o uso de psicotrópicos em casa dos 34 pacientes submetidos à CRM.

Tabela 12 - Análise descritiva dos sintomas de ansiedade cardíaca no pré-operatório dos 34 pacientes submetidos à cirurgia de revascularização do miocárdio, conforme sexo, idade, situação conjugal, situação profissional, uso de psicotrópicos em casa e valores de probabilidade (*p*) associados ao teste de Mann-Whitney. Ribeirão Preto, 2018-2019.

**Variáveis**	**Ansiedade Cardíaca**		
	**Escore total**<br>Mediana<br>(Mín-Máx)*	**Medo e Hipervigilância**<br>Mediana<br>(Mín-Máx)*	**Evitação**<br>Mediana<br>(Mín-Máx)*
**Sexo**			
Masculino (n = 22)	34,0 (4 – 46)	19,5 (4 – 29)	16,0 (0 – 20)
Feminino (n = 12)	35,0 (19 – 50)	18,0 (2 -30)	16,0 (9 – 20)
	p = 0, 631	*p* = 0,790	*p* = 0,873
**Idade**			
Idoso (n = 18)	35,5 (4 – 50)	16,5 (2 – 30)	18,5 (0 – 20)
Adulto (n = 16)	31,0 (13 – 46)	20,5 (9 – 20)	11,0 (0 – 20)
	p = 0,528	*p* = 0,330	***p*** = 0,036**
**Situação conjugal**			
Com companheiro (n = 23)	32,0 (4 – 50)	18,0 (2 – 30)	18,0 (0 – 20)
Sem companheiro (n = 11)	34,0 (13 – 46)	22,0 (5 – 29)	13,0 (2 – 20)
	p = 0,885	*p* = 0,561	*p* = 0, 383
**Situação profissional**			
Inativo (n = 20)	34,5 (15 – 50)	18,5 (5 – 30)	16,0 (9 – 20)
Ativo (n = 14)	30,0 (4 – 46)	19,0 (2 – 29)	15,0 (0 – 20)
	p = 0,245	*p* = 0,500	*p* = 0,274
**Uso de Psicotrópico em Casa**			
Não (n = 27)	34,0 (4 – 50)	21,0 (4 – 30)	17,0 (0 – 20)
Sim (n = 7)	20,0 (13 – 41)	9, 0 (2 – 26)	15, 0 (4 – 20)
	p = 0,066	***p*** = 0,031**	*p* = 0,357

* Min-Máx: Mínimo-Máximo; ** p<0,05: significância estatística;
Fonte: autora,2020.

Pacientes idosos submetidos à CRM apresentaram mediana maior no domínio "Evitação" (mediana=18,5) quando comparados com os adultos (mediana=11,0) e essa diferença foi estatisticamente significante (*p*=0,036). Já os pacientes que faziam uso de medicamentos psicotrópicos em casa apresentaram a mediana menor no domínio "Medo e hipervigilância" (9,0) quando comparados com aqueles que não faziam uso de psicotrópicos antes da internação (21,0) e essa diferença também estatisticamente significante (*p*=0,031).

Na tabela 13, encontram-se as medianas e os valores mínimos e máximos dos sintomas de ansiedade cardíaca no pré-operatório segundo o sexo, a idade, a situação conjugal, a situação profissional e o uso de psicotrópicos no domicílio dos 58 pacientes submetidos à correção cirúrgica de valvopatias.

Tabela 13 - Análise descritiva dos sintomas de ansiedade cardíaca no pré-operatório dos 58 pacientes submetidos à cirurgia para correção de valvopatia, conforme sexo, idade, situação conjugal, situação profissional, uso de psicotrópicos em casa e valores de probabilidade (*p*) associados ao teste de Mann-Whitney. Ribeirão Preto, 2018-2019.

**Variáveis**	**Ansiedade Cardíaca**		
	**Escore total**	**Medo e Hipervigilância**	**Evitação**
	Mediana (Mín-Máx)*	Mediana (Mín-Máx)*	Mediana (Mín-Máx)*
**Sexo**			
Feminino (n = 30)	39,0 (7 – 53)	23,5 (2 -34)	16,0 (0 – 20)
Masculino (n = 28)	30,5 (11 – 55)	20,0 (4 – 35)	20,0 (0 – 20)
	p = 0, 123	*p* = 0,161	*p* = 0,961
**Idade**			
Adulto (n = 33)	33,0 (17 – 51)	21,0 (4 – 34)	16,0 (5 – 20)
Idoso (n = 25)	34,0 (7 – 55)	22,0 (2 – 35)	18,0 (0 – 20)
	p = 0,392	*p* = 0,777	*p* = 0,444
**Situação conjugal**			
Com companheiro (n = 41)	37,0 (7 – 55)	23,0 (4 – 35)	20,0 (0 – 20)
Sem companheiro (n = 17)	32,0 (12 – 48)	16,0 (2 – 32)	16,0 (4 – 20)
	p = 0,169	*p* = 0,089	*p* = 0,394
**Situação profissional**			
Inativo (n = 35)	39,0 (7 – 55)	23,0 (2 – 35)	20,0 (0 – 20)
Ativo (n = 23)	30,0 (11 – 50)	21,0 (4 – 32)	15,0 (0 – 20)
	p = 0,059	*p*= 0,395	***p** = 0,028***
**Uso de Psicotrópico em Casa**			
Não (n = 36)	33,0 (11 – 55)	21,0 (2 – 35)	15,5 (0 – 20)
Sim (n = 22)	38,5 (7 – 53)	23, 0 (7 – 33)	19, 0 (0 – 20)
	p = 0,092	*p*= 0,251	*p* = 0,236

* Min-Máx: Mínimo-Máximo; ** *p*<0,05: significância estatística; *** Domínios QAC: Domínios do Questionário de Ansiedade Cardíaca.

Fonte: autora, 2020

Pacientes inativos submetidos à correção cirúrgica de valvopatias apresentaram mediana maior no domínio "Evitação" (mediana=20,0) quando comparados com os pacientes com situação profissional ativa (mediana=15,0) e essa diferença foi estatisticamente significante (*p*=0,028).

A tabela 14 apresenta as medianas e os valores mínimos e máximos dos sintomas de ansiedade cardíaca dos 34 pacientes submetidos à CRM no primeiro retorno hospitalar, segundo o sexo, a idade, a situação conjugal e a situação profissional.

Tabela 14 - Análise descritiva dos sintomas de ansiedade cardíaca no primeiro retorno hospitalar dos 34 pacientes submetidos à cirurgia de revascularização do miocárdio, conforme sexo, idade, situação conjugal, situação profissional e valores de probabilidade (*p*) associados ao teste de Mann-Whitney. Ribeirão Preto, 2018-2019.

Variáveis	Ansiedade Cardíaca		
	**Escore total**	**Medo e Hipervigilância**	**Evitação**
	Mediana (Mín-Máx)*	Mediana (Mín-Máx)*	Mediana (Mín-Máx)*
**Sexo**			
Masculino (n = 22)	36,0 (26 – 48)	18,0 (8 – 29)	20,0 (8 – 20)
Feminino (n = 12)	42,5 (18 – 48)	22,5 (6 – 30)	20,0 (8 – 20)
	p = 0,403	*p* = 0,582	*p* = 0,557
**Idade**			
Idoso (n = 18)	37,5 (28 – 48)	17,5 (6 – 29)	20,0 (15 – 20)
Adulto (n = 16)	40,5 (18 – 48)	21,5 (10 – 30)	20,0 (8 – 20)
	p = 0,905	*p* = 0,463	*p*= 0,211
**Situação conjugal**			
Com companheiro (n = 23)	38,0 (18 – 48)	18,0 (6 – 29)	20,0 (8 – 20)
Sem companheiro (n = 11)	43,0 (28 – 46)	25,0 (12 – 30)	20,0 (8 – 20)
	p = 0,612	*p* = 0,274	*p* = 0,201
**Situação profissional**			
Inativo (n = 20)	41,0 (18 – 48)	21,0 (6 – 30)	20,0 (8 – 20)
Ativo (n = 14)	36,5 (26 – 48)	18,5 (8 – 28)	20,0 (8 – 20)
	p = 0,717	*p* = 0,666	*p* = 0,877

* Min-Máx: Mínimo-Máximo;
Fonte: autora: 2020

Não foram encontradas associações dos sintomas de ansiedade cardíaca dos pacientes submetidos à CRM no momento do primeiro retorno hospitalar após a cirurgia cardíaca com o sexo, a idade, a situação conjugal e a situação profissional.

Na tabela 15 encontram-se as medianas e os valores mínimos e máximos dos sintomas de ansiedade cardíaca dos 58 pacientes submetidos às cirurgias para correção de valvopatias no dia do primeiro retorno hospitalar, segundo sexo, idade, situação conjugal e a situação profissional.

Tabela 15 - Análise descritiva dos sintomas de ansiedade cardíaca no primeiro retorno hospitalar dos 58 pacientes submetidos à cirurgia para correção de valvopatia, segundo sexo, idade, situação conjugal, situação profissional e valores de probabilidade (*p*) associados ao teste de Mann-Whitney. Ribeirão Preto, 2018-2019.

**Variáveis**	**Ansiedade Cardíaca**		
	**Escore total**	**Medo e Hipervigilância**	**Evitação**
	Mediana (Mín-Máx)*	Mediana (Mín-Máx)*	Mediana (Mín-Máx)*
**Sexo**			
Feminino (n = 30)	38,5 (20 – 56)	19,0 (6 – 36)	20,0 (10 – 20)
Masculino (n = 28)	40,0 (13 – 51)	22,0 (5 – 32)	20,0 (2 – 20)
	p = 0,815	*p* = 0,870	*p* = 0,923
**Idade**			
Adulto (n = 33)	39,0 (20 – 51)	19,0 (7 – 31)	20,0 (10 – 20)
Idoso (n = 25)	38,0 (13 – 56)	22,0 (5 – 36)	20,0 (2 – 20)
	p = 0,905	*p* = 0,463	*p*= 0,211
**Situação conjugal**			
Com companheiro (n = 41)	40,0 (13 – 53)	22,0 (5 – 33)	20,0 (2 – 20)
Sem companheiro (n = 17)	37,0 (20 – 56)	17,0 (7 – 36)	20,0 (8 – 20)
	p = 0,228	*p* = 0,190	*p* = 0,603
**Situação profissional**			
Inativo (n = 35)	38,0 (20 – 56)	18,0 (6 – 36)	20,0 (10 – 20)
Ativo (n = 23)	39,0 (13 – 51)	22,0 (5 – 32)	20,0 (2 – 20)
	p = 0,622	*p* = 0,981	*p* = 0,152

* Min-Máx: Mínimo-Máximo.
Fonte: autora, 2020

Não foram encontradas associações dos sintomas de ansiedade cardíaca dos pacientes submetidos à correção cirúrgica de valvopatias no momento do primeiro retorno hospitalar após a cirurgia cardíaca com o sexo, a idade, a situação conjugal e a situação profissional.

4.4 CORRELAÇÃO DOS SINTOMAS DE ANSIEDADE CARDÍACA COM OS SINTOMAS DE ANSIEDADE E DEPRESSÃO DOS PACIENTES NO PRÉ-OPERATÓRIO, NO DIA DA ALTA HOSPITALAR E NO PRIMEIRO RETORNO APÓS A ALTA HOSPITALAR

Os resultados das correlações entre os sintomas de ansiedade cardíaca e os sintomas de ansiedade e depressão no pré-operatório, no dia da alta e no primeiro retorno dos 34 pacientes submetidos à CRM encontram-se nas tabelas 16 e 17.

Tabela 16 - Correlação dos sintomas de ansiedade cardíaca com os sintomas de ansiedade dos 34 pacientes submetidos à cirurgia de revascularização do miocárdio com os respectivos valores de probabilidade (*p*) associados ao teste de correlação de Spearman. Ribeirão Preto, 2018-2019.

**Variáveis**	**HADS* - Ansiedade**					
	**Pré-Operatório**		**Alta**		**Primeiro Retorno**	
	**r**	***p***	**r**	***p***	**r**	***p***
Ansiedade Cardíaca	0,395	**0,021****	0,377	**0,028****	0,468	**0,005****
**Domínios QAC*****						
Medo e Hipervigilância	0,543	**0,001****	0,481	**0,004****	0,476	**0,004****
Evitação	-0,002	0,992	-0,028	0,877	-0,001	0,994

*HADS - *Hospital Anxiety and Depression Scale*; ** *p*<0,05: significância estatística; *** Domínios QAC: Domínios do Questionário de Ansiedade Cardíaca.
Fonte: autora, 2020

Foram encontradas correlações de moderada magnitude dos sintomas de AC (escore total) com os sintomas de ansiedade nos três tempos investigados e todas foram estatisticamente significantes. Ao se analisar a correlação do domínio “Medo e hipervigilância” da AC com os sintomas de ansiedade no pré-operatório, observa-se que foi de forte magnitude e significativa, ao passo que no momento da alta e primeiro retorno, as correlações foram moderadas e significativas.

Não foram encontradas correlações do domínio “Evitação” da AC com os sintomas de ansiedade nos três tempos.

Tabela 17 - Correlação dos sintomas de ansiedade cardíaca com os sintomas de depressão dos 34 pacientes submetidos à cirurgia de revascularização do miocárdio com os respectivos valores de probabilidade (*p*) associados ao teste de correlação de Spearman. Ribeirão Preto, 2018-2019.

**Variáveis**	**HADS* - Depressão**					
	**Pré-Operatório**		**Alta**		**Primeiro Retorno**	
	**r**	***p***	**r**	***p***	**r**	***p***
Ansiedade Cardíaca	0,214	0,225	0,144	0,418	0,297	0,088
**Domínios QAC****						
Medo e Hipervigilância	0,279	0,110	0,077	0,663	0,300	0,085
Evitação	0,063	0,725	0,244	0,164	-0,019	0,913

*HADS - *Hospital Anxiety and Depression Scale*; ** Domínios QAC: Domínios do Questionário de Ansiedade Cardíaca.
Fonte: autora, 2020

Não foram encontradas correlações dos sintomas de AC com os sintomas de depressão nos três momentos avaliados.

Os resultados das correlações entre os sintomas de ansiedade cardíaca e os sintomas de ansiedade e depressão no pré-operatório, no dia da alta e no primeiro retorno dos 58 pacientes submetidos à correção cirúrgica de valvopatias encontram-se nas tabelas 18 e 19.

Tabela 18 - Correlação dos sintomas de ansiedade cardíaca com os sintomas de ansiedade dos 58 pacientes submetidos à cirurgia para correção de valvopatia com os respectivos valores de probabilidade (*p*) associados ao teste de correlação de Spearman. Ribeirão Preto, 2018-2019.

**Variáveis**	**HADS* - Ansiedade**					
	**Pré-Operatório**		**Alta**		**Primeiro Retorno**	
	**r**	***p***	**r**	***p***	**r**	***p***
Ansiedade Cardíaca	0,276	**0,036****	0,234	0,077	0,241	0,068
**Domínios QAC*****						
Medo e Hipervigilância	0,220	0,097	0,250	0,058	-0,040	0,764
Evitação	0,209	0,115	0,037	0,780	0,249	0,060

*HADS - *Hospital Anxiety and Depression Scale*; ** p<0,05: significância estatística; *** Domínios QAC: Domínios do Questionário de Ansiedade Cardíaca.
Fonte: autora, 2020

Foi encontrada correlação de fraca magnitude dos sintomas de AC (escore total) com os sintomas de ansiedade no pré-operatório, com significância estatística.

Tabela 19 - Correlação dos sintomas de ansiedade cardíaca com os sintomas de depressão dos 58 pacientes submetidos à cirurgia para correção de valvopatia com os respectivos valores de probabilidade (*p*) associados ao teste de correlação de Spearman. Ribeirão Preto, 2018-2019.

**Variáveis**	**HADS* - Depressão**					
	**Pré-Operatório**		**Alta**		**Primeiro Retorno**	
	**r**	***p***	**r**	***p***	**r**	***p***
Ansiedade Cardíaca	0,336	**0,010****	0,153	0,252	0,066	0,623
**Domínios QAC*****						
Medo e Hipervigilância	0,327	**0,012****	0,178	0,181	0,069	0,608
Evitação	0,114	0,395	-0,044	0,744	-0,063	0,639

*HADS - *Hospital Anxiety and Depression Scale*; ** p<0,05: significância estatística; *** Domínios QAC: Domínios do Questionário de Ansiedade Cardíaca.
Fonte: autora, 2020

Foram encontradas correlações de moderada magnitude dos sintomas de AC (escore total) com os sintomas de depressão no pré-operatório, com significância estatística. Ao se analisar a correlação do domínio “Medo e hipervigilância” da AC com os sintomas de depressão no pré-operatório, observa-se que ela foi de moderada magnitude e significativa.

5. DISCUSSÃO

Retomando o objetivo de comparar os sintomas de ansiedade cardíaca em pacientes submetidos às cirurgias de revascularização do miocárdio e de correção cirúrgica de valvopatia no pré-operatório, no dia da alta hospitalar e no primeiro retorno após a alta hospitalar, observamos o efeito do tempo nos sintomas de ansiedade cardíaca dos pacientes submetidos à CRM no escore total e no domínio “Evitação”, sendo que esses pacientes apresentaram mais sintomas de AC (escore total e “Evitação”) no dia da alta quando comparados com o pré-operatório, assim como apresentaram mais sintomas no dia do primeiro retorno quando comparados com o pré-operatório.

Já nos pacientes submetidos à correção cirúrgica de valvopatias, observamos também o efeito do tempo nos sintomas de ansiedade cardíaca no escore total e no domínio “Evitação”, ou seja, os pacientes apresentaram mais sintomas de AC (escore total e “Evitação”) no primeiro retorno quando comparados com a sintomatologia no pré-operatório.

Não encontramos na literatura pesquisas que investigaram a AC de pacientes submetidos à CRM e à correção cirúrgica de valvopatias nos três tempos investigados no presente estudo.

Encontramos um estudo realizado na Alemanha, no qual os pesquisadores avaliaram a AC de 90 pacientes submetidos às cirurgias de CRM (n=60), troca de válvula (n=22) e cirurgias combinadas (n=8) em três momentos: no pré-operatório, duas semanas antes da cirurgia, seis semanas após a cirurgia e seis meses após a cirurgia. Os dados foram analisados considerando a amostra total, sendo encontrado que no escore total da AC os pacientes apresentaram diminuição dos sintomas seis semanas e seis meses após a cirurgia quando comparados com o pré-operatório (p<0,001). Quanto ao domínio “Medo”, os pacientes apresentaram melhora dos sintomas no PO quando comparados com o pré-operatório, mas sem diferença nos tempos de seis semanas e seis meses. Com relação ao domínio “Evitação”, não houve diferença no pré-operatório e seis semanas, mas os pacientes apresentaram melhoras nos sintomas seis meses após a cirurgia. Não encontraram diferenças na percepção dos sintomas nos três tempos no domínio “Atenção” (HOYER et al., 2008).

Conforme explanado na introdução, o instrumento original continha 18 itens, e esses itens eram distribuídos em três domínios, a saber: “Medo”, “Evitação” e “Atenção”, além de ser possível a avaliação da soma geral dos itens. No processo de validação para o Brasil, o instrumento permaneceu com 14 itens e os domínios “Medo” e “Atenção” foram unidos.

Mesmo que os tempos investigados no presente estudo diferem do estudo de Hoyer e colaboradores (2008), podemos observar a diferença na evolução dos sintomas. Na presente pesquisa, os pacientes vivenciaram piora da sintomatologia da AC no PO, ao passo que, no estudo de Hoyer e colaboradores (2008), os pacientes vivenciaram melhora dos sintomas. Não há descrição no artigo de Hoyer e colaboradores (2008) da rotina de atendimento aos pacientes submetidos às cirurgias cardíacas que são atendidos no hospital em que foi realizado o estudo. Mas vale ressaltar que no hospital que foi realizado o estudo em tela, é de rotina do hospital que todas as informações referentes à alta hospitalar são passadas no dia e no momento da alta hospitalar. Durante o primeiro retorno após a alta hospitalar, é realizada uma avaliação geral da condição de saúde do paciente. A rotina instituída do hospital pode ter favorecido os resultados encontrados.

Além disso, quando retomamos os itens que compõem especificamente o domínio "Evitação", a saber: *evito esforço*, *pego leve o máximo possível*, *evito fazer exercícios ou outras atividades físicas*, *evito atividades que acelerem o meu coração* e *evito atividades que me façam suar*, ressalta-se que no hospital do estudo o serviço de reabilitação cardíaca não absorve 100% desses pacientes. Todas as orientações relacionadas ao esforço e retomada das atividades físicas são fornecidas no dia da alta hospitalar e reforçadas no primeiro retorno, fatores esses que podem ter contribuído para a piora desses sintomas no PO. Essas informações são passadas, na sua grande maioria, pelos médicos e fisioterapeutas.

Encontramos também um estudo realizado na Bahia, cujo objetivo foi investigar os sintomas de AC de 25 pacientes submetidos à CRM, troca de válvula mitral e/ou aórtica e correção de comunicação interatrial no pré-operatório e sete dias de PO. Os pacientes apresentaram mais sintomas de AC cardíaca no pré-operatório (média=50; DP=17) quando comparados com o PO (média=29; DP=11), com diferença significativa (p=0,001), diferentemente dos resultados encontrados na presente pesquisa (CORDEIRO et al., 2015).

Diante da escassez de estudos de delineamento longitudinal sobre a sintomatologia da AC em pacientes submetidos às cirurgias cardíacas, bem como diante dos diferentes resultados encontrados, novas pesquisas são necessárias para elucidar o papel desse tipo específico de ansiedade dos pacientes no perioperatório dessas cirurgias.

Encontramos ainda na literatura estudos transversais nos quais a AC foi investigada em pacientes submetidos à cirurgias cardíacas. Aicher e colaboradores (2011) realizaram um estudo na Alemanha no qual compararam a qualidade de vida, os sintomas de ansiedade, a depressão e a AC de 166 pacientes jovens que foram submetidos à três tipos de cirurgias para correção de valvopatias: plastia valvar, troca de válvula por prótese mecânica e operação de

Ross (auto enxerto pulmonar). Pacientes submetidos à troca valvar por prótese mecânica apresentaram maior escore no domínio “Medo” quando comparados com pacientes submetidos à operação de Ross. Com relação ao domínio “Atenção”, pacientes submetidos à plastia e à operação de Ross apresentaram maior escore quando comparados com pacientes que tiveram a troca por prótese mecânica. Não foram encontradas diferenças significativas no domínio “Evitação”.

Já Moraes e colaboradores (2013) realizaram um estudo em Pernambuco com o objetivo de avaliar a AC de 91 pacientes que haviam sido submetidos à cirurgia cardíaca num período de até cinco anos. Os autores não especificaram o tipo de cirurgia cardíaca. Além disso, utilizaram a versão traduzida do instrumento, então, nos resultados constam os 18 itens (SARDINHA et al., 2008). Os autores utilizaram um ponto de corte para classificar os pacientes como “sintomas de leve a moderado” e de “moderado a grave”. Não foram encontradas associações dos sintomas de AC com a faixa etária e o sexo (MORAES et al., 2013).

Quanto aos resultados vinculados ao objetivo da investigação da associação dos sintomas de ansiedade cardíaca com sexo, idade, situação conjugal e vínculo empregatício em pacientes submetidos às cirurgias de revascularização do miocárdio e de correção de valvopatias no pré-operatório e no primeiro retorno, no pré-operatório os pacientes idosos submetidos à CRM apresentaram maior sintomatologia no domínio “Evitação” quando comparados com os adultos, bem como os pacientes que foram submetidos à cirurgia de correção de valvopatias inativos apresentaram maior sintomatologia no domínio “Evitação”. Não foram encontradas associações dos sintomas de ansiedade cardíaca dos pacientes submetidos à CRM e dos submetidos à correção cirúrgica de valvopatias no momento do primeiro retorno hospitalar após a cirurgia cardíaca com as variáveis acima descritas.

Van Beek e colaboradores (2016) desenvolveram um estudo na Holanda com o objetivo de investigar a associação da AC com o prognóstico de pacientes internados com IAM. Os pacientes foram entrevistados durante a internação e quatro meses após a alta. Embora os pacientes estivessem em atendimento clínico, não cirúrgico, durante a internação, os idosos também apresentaram maior sintomatologia de AC (p=0,004). A sintomatologia da AC avaliada na internação foi significativamente associada com maior risco de desenvolvimento de eventos cardíacos secundários graves, independentemente da idade e sexo, ou seja, quando maior a sintomatologia na internação, maior risco de desenvolvimento desses eventos após a alta.

Fischer e colaboradores (2012) desenvolveram um estudo na Alemanha com o objetivo de investigar a AC na população geral para identificar valores padrões dessa sintomatologia e

observaram um aumento significativo dos sintomas de AC com o aumento da idade ($p<0,001$), tanto para o escore total ($p<0,001$) quanto para as subescalas ($p<0,001$).

Com relação à associação da ausência de atividade laboral com piores sintomas de AC no domínio “Evitação” entre pacientes submetidos à correção cirúrgica de valvopatias, no estudo realizado para investigar a AC na população geral, os autores identificaram que a variável situação profissional, até na população geral, tem efeito sobre a sintomatologia de AC, tanto no escore total ($p<0,001$) quanto nos três domínios ($p<0,001$), ou seja, pessoas aposentadas apresentaram mais sintomatologia de AC ($<0,050$) quando comparadas com pessoas não aposentadas (FISCHER et al., 2012).

Ressalta-se mais uma vez a natureza das questões que constituem o domínio “Evitação” da AC, perguntas relacionadas ao esforço e à realização de atividades físicas. Após um evento cardíaco, os pacientes recebem orientações de restrições que podem ser excessivas e restritivas à prática de exercícios, o que pode interferir na falta de motivação e insegurança para o retorno às atividades rotineiras pelos pacientes idosos (ADAMS et al., 2006).

Autores alemães desenvolveram um estudo com o objetivo de investigar a associação da AC com a depressão e com hábitos de vida (tabagismo atual, consumo de álcool, participação em grupo de reabilitação cardíaca e realização de atividade física com frequência) de 1007 pacientes com doença arterial coronariana. Os pacientes foram entrevistados seis meses após a alta decorrente da internação pela DAC. Maiores escores no domínio “Evitação” foram significativamente associados com o tabagismo atual, baixa frequência de atividade física, não participação em grupo de reabilitação cardíaca e redução do consumo de álcool (HOHLS et al., 2020).

Já existem evidências na literatura de que pacientes com cardiopatias aderentes à realização de atividades físicas regulares apresentaram melhores avaliações da AC quando comparados com pacientes cardiopatas sedentários. Sardinha e colaboradores (2012) realizaram um estudo no Rio de Janeiro com 119 pacientes coronariopatas, cujo objetivo foi de comparar os sintomas de AC de pacientes sedentários com pacientes que realizavam atividades físicas regulares. O grupo de pacientes sedentários foi composto por 59 pacientes, ao passo que o grupo de praticantes de atividades físicas foi de 60. A média do escore de AC do grupo de pacientes sedentários foi significativamente maior do que a média dos pacientes que praticavam atividades físicas.

Ainda na presente investigação encontramos associação da AC com o uso de psicotrópicos, ou seja, os pacientes submetidos à CRM que não faziam uso de medicamentos psicotrópicos em casa apresentaram maior sintomatologia no domínio “Medo e hipervigilância”

quando comparados com aqueles que faziam uso de psicotrópicos antes da internação. Não encontramos na literatura comparações entre a sintomatologia de AC de pacientes submetidos à cirurgias cardíacas e o uso de psicotrópicos.

Sardinha e colaboradores (2011) desenvolveram um estudo no qual investigaram a presença de Ansiedade Relacionada à Saúde (ARS) e a ansiedade cardíaca em pacientes com DAC que frequentavam regularmente um programa de exercícios supervisionado e encontraram que 26% dos participantes do estudo relataram uso de benzodiazepínicos quando ansiosos, estressados ou com dificuldades para dormir, sendo que apenas um paciente afirmou receber tratamento para ansiedade por seu cardiologista.

Encontramos também correlação dos sintomas de AC em pacientes submetidos à CRM (escore total e domínio "Medo e hipervigilância") com os sintomas de ansiedade nos três tempos investigados. Já no grupo de pacientes submetidos à correção cirúrgica de valvopatias, encontramos correlação dos sintomas de AC com os sintomas de ansiedade, porém apenas o escore total no pré-operatório, sendo que a correlação foi de fraca magnitude.

Por outro lado, não encontramos correlação dos sintomas de AC com os sintomas de depressão nos pacientes submetidos à CRM, ao passo que identificamos correlações dos sintomas de AC (escore total e "Medo e hipervigilância") com os sintomas de depressão no pré-operatório de pacientes submetidos à correção cirúrgica de valvopatias.

No estudo de Hoyer e colaboradores (2008), os autores identificaram que o escore total e os três domínios da AC obtiveram correlação positiva com os sintomas de ansiedade e de depressão, tanto no pré-operatório quanto no pós-operatório de cirurgias cardíacas, exceto para o domínio "Atenção" da AC com os sintomas de depressão no pré-operatório, cuja correlação não foi significativa. Os autores ainda afirmaram que as correlações tendem a ser mais fortes e frequentes dos sintomas de AC com os sintomas de ansiedade, quando comparadas com as correlações dos sintomas de AC com sintomas de depressão.

Quanto à caracterização sociodemográfica e clínica dos pacientes, observamos que a maioria dos pacientes submetidos à CRM pertencia ao sexo masculino, resultado que corrobora com outros estudos da literatura (JANSSEN et al., 2015; SOUSA et al., 2015; SILVA, L. N. et al., 2018; KAFADAR et al., 2018; KRZEMIŃSKA et al., 2019; ADHIKARI; BARAL, 2018; SILVA; MELO; NEVES, 2019; CALLES et al., 2016; POOLE et al., 2017; ALEXANDRI et al., 2017; BANO et al., 2020;KSHIRSAGAR et al., 2020; NIELSEN et al., 2019; MURPHY et al., 2020; PERROTTI et al., 2019; AXELSSON et al., 2020; FORMENTINI et al., 2019; YANG et al., 2020; HOHLS et al., 2020; PACARIC et al., 2020).

Por outro lado, a maioria dos pacientes submetida à correção cirúrgica de valvopatias era do sexo feminino, resultado que corrobora com muitos estudos da literatura (ANJOS et al., 2016; CALÇA et al., 2019; FU et al., 2020; FUKUNAGA; SAKATA; KOYMA, 2018; DE LIMA JÚNIOR; MATIAS; STAHLKE JÚNIOR, 2019; KAPADIA et al., 2020; COSTA et al., 2016; OLIVEIRA, 2017; PEREIRA, 2019; FLORENTINO et al., 2018; LI et al., 2019; DORDETTO; PINTO; ROSA, 2016; CHEN et al., 2019), porém difere de outros (GUEDENEY et al., 2019; KWEDAR et al., 2017; LIN et al., 2019; MARTINS, 2016; MODICA et al., 2018; RODRIGUES et al., 2020).

Com relação a presença de companheiro e a situação profissional dos pacientes submetidos à CRM, a maior parte possuía companheiro e houve predominância de situação profissional inativa, corroborando com outros achados da literatura (JANSSEN et al., 2015; KRZEMIŃSKA et al., 2019; SILVA, L. D. C. et al., 2018; NIELSEN et al., 2019), assim como para os pacientes do grupo de correção valvar (ANJOS et al., 2016; OTERHALS et al., 2015; DORDETTO; PINTO; ROSA, 2016; RODRIGUES et al., 2020; CHEN et al., 2019; GIARETTA et al., 2018; HUSSAIN et al., 2018).

A média de idade dos pacientes submetidos à CRM foi de 62,4 anos, evidenciando pacientes mais idosos, achados estes que corroboram com a literatura, variando de 57,3 a 65 anos (PERROTTI et al., 2019; SILVA, L. D. C. et al., 2018; SILVA, L. N. et al., 2018; ADHIKARI; BARAL, 2018; ARAÚJO et al., 2017; SILVA; MELO; NEVES, 2019; FORMENTINI et al., 2019).

No grupo de pacientes submetidos à correção cirúrgica valvar, a média de idade foi de 54,7 anos, corroborando com achados da literatura, nos quais pesquisadores encontraram valores de 52 a 58 anos em média (FU et al., 2020; ANJOS et al., 2016; OLIVEIRA, 2017; RODRIGUES et al., 2020; CHEN et al., 2019). Entretanto, alguns estudos encontraram maiores médias de idade em pacientes valvopatas, com variação de 81,8 a 84,2 anos (CALÇA et al., 2019; GUEDENEY et al., 2019; KAPADIA et al., 2020; LI et al., 2019).

Em relação a escolaridade, os pacientes submetidos à CRM apresentaram, em média, 7,2 anos de estudo, indicando baixo nível educacional, corroborando com o resultado encontrado por Nielsen e colaboradores (2019), no qual 48,2% dos indivíduos coronariopatas possuíam menos de 10 anos de estudo, bem como o resultado encontrado por Rodrigues e colaboradores (2018), com uma média de 5,4 anos de estudo. Outras pesquisam mostram resultados semelhantes, nos quais os indivíduos não possuíam ensino fundamental completo, variando de 35,4 % a 72,9% (SILVA, L. D. C. et al., 2018; PRADO-OLIVARES; CHOVER-SIERRA, 2019; MERTINS et al., 2016).

A baixa escolaridade também foi encontrada no grupo de pacientes submetidos à correção cirúrgica de valvopatias, uma média de 6,4 anos de estudo, corroborando com o resultado encontrado por Oterhals e colaboradores (2015), no qual 47% dos indivíduos valvopatas possuíam menos de 9 anos de estudo, e com o estudo de Modica e colaboradores (2018), que evidenciou que 54,3% dos pacientes possuíam baixo nível educacional. No estudo de Anjos e colaboradores (2016), a média de escolaridade encontrada foi idêntica à do presente estudo.

Dentre as doenças associadas apresentadas pelos pacientes submetidos à CRM, a HAS foi a que apresentou maior frequência (97,1%), seguida por sobrepeso/obesidade (79,4%), dislipidemia (70,6%) e diabetes mellitus (58,8%). Em outros estudos presentes na literatura, a HAS também foi a doença associada mais frequentemente encontrada nesse tipo de paciente, variando entre 62,4% e 92,1% (SILVA; MELO; NEVES, 2019; SILVA, L. D. C. et al., 2018; KRZEMIŃSKA et al., 2019; RODRIGUES et al., 2018; JANSSEN et al., 2015; CALLES et al., 2016; SOUSA et al., 2015; RAMESH et al., 2017).

Os resultados encontrados por Sousa e colaboradores (2015) corroboram com os encontrados neste estudo, evidenciando o sobrepeso/obesidade como a segunda doença associada mais frequente (45,1%), porém, difere dos resultados encontrados por Mertins e colaboradores (2016), nos quais o sobrepeso e obesidade ficaram em sexto e sétimo lugar entre as doenças associadas mais frequentes, com 35,5% e 33,4%, respectivamente.

Barbosa e colaboradores (2018) investigaram o impacto dos fatores de risco para DAC nos custos com a cirurgia de revascularização do miocárdio e observaram que indivíduos que possuíam maior número de doenças associadas apresentaram IMC mais elevado quando comparados com indivíduos com menos fatores de risco ($p < 0,001$), de forma que o grupo de indivíduos com apenas uma doença associada possuía IMC de 25,7kg/m^2 e o grupo de indivíduos com seis doenças associadas possuía IMC de 31,4kg/m^2. O número de doenças associadas podem ser, então, um fator relacionado à variação da frequência de sobrepeso e obesidade nos diferentes estudos.

Quanto a dislipidemia, cuja porcentagem foi de 70,6%, no presente estudo foi ranqueada como a terceira doença associada mais frequente, diferentemente de outros estudos, nos quais encontraram porcentagens menores, variando de 21,2% a 18,5% (ADHIKARI; BARAL 2018; JASSEN et al., 2015). Em outro estudo, a dislipidemia foi encontrada como a segunda doença associada mais frequente, com uma porcentagem de 23,7% (SILVA, L. D. C. et al., 2018).

Já o diabetes mellitus, a quarta doença associada com maior frequência (58,8%) no nosso estudo, pode ser encontrado na literatura como a segunda mais frequente, quase sempre

após a HAS, variando de 34% a 50,8% (ADHIKARI; BARAL, 2018; JANSSEN et al., 2015; ARAÚJO et al., 2017; SILVA, L. D. C. et al., 2018).

Axelsson e colaboradores (2020) investigaram as complicações e mortalidade a longo prazo de pacientes submetidos à cirurgia do revascularização do miocárdio, segundo a presença ou ausência de diabetes mellitus, e encontraram que os pacientes portadores de diabetes mellitus tiveram pior sobrevida a longo prazo quando comparados com aqueles sem diabetes, tanto na avaliação da sobrevida em cinco anos (com diabetes= 85%; sem diabetes= 91%), quanto na avaliação de sobrevida em 10 anos (com diabetes= 64% e sem diabetes= 77%). Além disso, o risco relativo de mortalidade é quatro vezes maior para pacientes que possuem diabetes quando comparados com a população em geral e pode se tornar ainda mais alto na presença de outras doenças associadas (BERGER; STENSTRÖNM; SUNDKVIST, 1999; ÖSTGREN et al., 2002).

Quanto às doenças associadas apresentadas pelos pacientes submetidos à correção cirúrgica de valvopatias, a HAS foi a mais frequente (72,4%), seguida por sobrepeso/obesidade (67,2%) e dislipidemia (41,3%). Em outros estudos presentes na literatura, a HAS também foi a doença associada mais frequentemente encontrada, variando entre 32,1% e 85% (CALÇA et al., 2019; FLORENTINO et al., 2018; FUKUNAGA; SAKATA; KOYMA, 2018; GUEDENEY et al., 2019; KWEDAR et al., 2017; LIN et al., 2019; RODRIGUES et al., 2020; MARTINS, 2016; AUENSEN et al., 2017; HUSSAIN et al., 2018; PETERSEN et al., 2016; CETINKAYA et al., 2019).

Nossos resultados corroboram com os resultados encontrados por Rodrigues e colaboradores (2020) no que diz respeito ao sobrepeso/obesidade, sendo a doença associada mais frequente (48,5%). Já no estudo de Calça e colaboradores (2019), a obesidade foi a quarta doença associada mais encontrada (17,2%). Outros estudos evidenciaram uma média de Índice de Massa Corporal de 26 a 28 kg/m^2 em pacientes valvopatas, caracterizando, então, sobrepeso (FLORENTINO et al., 2018; AUENSEN et al., 2017; GUEDENEY et al., 2019; SANTOS et al., 2018; HUSSAIN et al., 2018).

A dislipidemia, terceira doença associada que apareceu com maior frequência (41,3%), foi também encontrada nesta posição em outros trabalhos, variando de 34,1% a 65,6% (RODRIGUES et al., 2020; FUKUNAGA; SAKATA; KOYMA, 2018; LIN et al., 2019). Porém, esses dados diferem dos encontrados por Florentino e colaboradores (2017) e Petersen e colaboradores (2016), nos quais a dislipidemia foi encontrada como a segunda doença associada mais frequente, com 65,6% e 66,7%, respectivamente.

Outros autores identificaram como segunda/terceira doença associada mais frequente o diabetes mellitus, com variação de 14% a 30,9% (CALÇA et al., 2019; FLORENTINO et al., 2018; FUKUNAGA; SAKATA; KOYMA, 2018; GUEDENEY et al., 2019; KWEDAR et al., 2017; PETERSEN et al., 2016), diferentemente do encontrado no nosso estudo, no qual o diabetes mellitus foi a quinta mais frequente, com 13,3%.

Com relação aos hábitos de vida dos pacientes submetidos à CRM, 52,9% dos participantes relataram tabagismo pregresso. Na literatura, a porcentagem encontrada para tabagismo pregresso em outros estudos realizados com coronariopatas variou de 12,5% a 36% (MERTINS et al., 2016; SOUSA et al., 2015; SCHLYTER et al., 2016). Quanto ao tabagismo ativo, relatado por 11,8% dos pacientes, a porcentagem encontrada em outros estudos variou de 13,8% a 46,4% (SILVA, L. D. C. et al., 2018; FORMENTINI et al., 2019; KSHIRSAGAR et al., 2020; MERTINS et al., 2016; RAMESH et al., 2017; SOUSA et al., 2015; MURPHY et al., 2020).

Yang e colaboradores (2020) realizaram um estudo com o objetivo de comparar os efeitos do tabagismo nos resultados clínicos de homens e mulheres submetidos à revascularização do miocárdio e encontraram que homens e mulheres tabagistas possuíam maior risco para eventos cardiovasculares adversos graves quando comparados com aqueles não tabagistas ($p=0,001$). Além disso, evidenciaram também que cessar o tabagismo foi mais desafiador para as mulheres do que para os homens ($p<0,001$), durante o seguimento de três anos, período no qual 48% dos homens e 30,7% das mulheres cessaram o tabagismo.

De acordo com a Organização Pan-Americana de Saúde (OPAS), fatores de risco comportamentais como, por exemplo, dietas inadequadas, sedentarismo e uso de tabaco podem levar ao aparecimento da HAS, hiperlipidemia, hiperglicemia, sobrepeso e obesidade, elevando assim o risco de desenvolvimento de doenças cardiovasculares (OPAS, 2020). Segundo a American Heart Association (AHA), possuir um estilo de vida saudável, com boa nutrição, controle de peso e prática de exercício físico, pode desempenhar um importante papel na prevenção de doenças coronarianas (AHA, 2020).

Com relação aos hábitos de vida dos pacientes submetidos ao tratamento cirúrgico das valvopatias, 41,4% relataram tabagismo pregresso. Na literatura, a porcentagem encontrada para tabagismo pregresso em pacientes valvopatas variou de 17,1% a 62% (YAFTIAN et al., 2020; SANTOS et al., 2018; RODRIGUES et al., 2020; OTERHALS et al., 2017). Em relação ao tabagismo ativo, relatado por 10,3% dos pacientes, a porcentagem encontrada em outros estudos variou de 8% a 25% (OTERHALS et al., 2017; PETERSEN et al., 2016; LIN et al., 2019; RODRIGUES et al., 2020).

Diante do exposto, observamos que a maioria dos pacientes submetidos à CRM já apresentava, no pré-operatório, HAS, sobrepeso/obesidade, dislipidemia, diabetes mellitus e idade mais avançada, elucidando o perfil de gravidade desses pacientes.

Encontramos também um comprometimento dos pacientes submetidos à cirurgia para correção de valvopatias, sendo que a maioria também já apresentava HAS e sobrepeso/obesidade, além de que uma grande porcentagem já apresentava dislipidemia.

5.1 LIMITAÇÕES DO ESTUDO

Uma das limitações do estudo está no tamanho da amostra. Mesmo realizando a coleta de dados por 18 meses, tendo em vista que o cronograma cirúrgico previa de três a quatro cirurgias por semana, muitas vezes os procedimentos foram cancelados por falta de material, queda de energia no centro cirúrgico, urgências e intercorrências com o paciente. Mudanças na escala cirúrgica também prejudicaram a coleta de dados.

Outra limitação está nos tempos investigados. Como a última coleta se encerrou no dia do primeiro retorno, não temos conhecimento dos sintomas de AC a longo prazo, como evidenciado em alguns estudos na literatura.

Durante a revisão de literatura evidenciamos que até o momento existem poucos artigos abordando essa temática. Assim, acreditamos na importância da realização de novos estudos com o objetivo de fornecer novas evidências que favoreçam tanto a recuperação fisiológica quanto a reabilitação psicossocial a longo prazo.

5.2 CONTRIBUIÇÕES DO ESTUDO PARA A PRÁTICA CLÍNICA

Considerando que a presença dos sintomas de AC por tempo prolongado pode acarretar complicações tanto para recuperação física, quanto para a reabilitação psicossocial (PETERSEN et al., 2016; BOTZET et al., 2018; HOYER et al., 2008; AICHER et al., 2011; RYMASZEWSKA; KIEJNA; HADRYS, 2003), acreditamos que a identificação precoce dos sintomas de AC se torna importante, não só pelo aumento da procura por atendimento médico, o que pode gerar um gasto elevado para sistema de saúde, mas também pelo fato de que pacientes que apresentaram sintomas de AC possuem maior risco para eventos adversos cardíacos graves (HOHLS et al, 2020; HOYER et al., 2008; TREMBLAY et al., 2018; FENG et al., 2016; VAN BEEK et al., 2016; MARKER; CARMIN; OWNBY, 2008).

Evidenciamos que pacientes idosos submetidos à CRM, bem como pacientes inativos submetidos à cirurgia para correção de valvopatias, apresentaram maior sintomatologia de AC, sendo assim, essa informação deve ser considerada pela equipe multidisciplinar ao elaborar os seus protocolos de atendimento no perioperatório.

Ressalta-se também que escores altos de AC podem trazer consequências financeiras, uma vez que pacientes que apresentam dor torácica e transtornos de ansiedade possuem mais consultas médicas reportadas, sugerindo que pacientes com sintomatologia de AC tem uma maior preocupação, além de mais atenção em sintomas cardíacos, com medo de apresentarem algum tipo de evento cardíaco, aumentando assim o uso do sistema de saúde (MOURAD et al., 2016; WHITE; CRAFT; GERVINO, 2010).

O aumento da procura por atendimento hospitalar relacionada com a sintomatologia de AC pode estar associado com o aumento da percepção dos sintomas de AC, o qual é representado pelo domínio "Atenção" (HOHLS et al., 2020). Mesmo quando corrigido pelas comorbidades, indivíduos com sintomatologia de AC apresentaram uma busca maior por atendimento médico ($p<0{,}001$), sendo AC a única variável independente associada com o aumento da procura por atendimento médico. (TREMBLAY et al., 2018; MOURAD et al., 2016).

6. CONCLUSÃO

Concluímos que pacientes submetidos à CRM apresentaram mais sintomas de AC total no dia da alta hospitalar quando comparados com o pré-operatório, assim como apresentaram maior sintomatologia de AC no dia do primeiro retorno comparados com o pré-operatório. Apresentaram também mais sintomatologia no domínio "Evitação" na alta e no primeiro retorno quando comparada com o pré-operatório.

Quanto aos pacientes submetidos à correção cirúrgica de valvopatia, os sintomas de AC total no primeiro retorno hospitalar foram maiores quando comparados com os do pré-operatório. Além disso, esses pacientes apresentaram maior sintomatologia de AC no domínio "Evitação" no primeiro retorno hospitalar quando comparados com o pré-operatório.

Com relação a associação dos sintomas de AC com as variáveis sociodemográficas e clínicas, pacientes idosos submetidos à CRM apresentaram maior sintomatologia no domínio "Evitação" quando comparados com pacientes adultos. Além disso, pacientes que não faziam uso de psicotrópicos em casa apresentaram maior sintomatologia no domínio "Medo e Hipervigilância" quando comparados com aqueles que faziam uso.

No grupo de pacientes submetidos à correção cirúrgica de valvopatias, pacientes com situação profissional inativa apresentaram maior sintomatologia no domínio "Evitação" quando comparados com pacientes com situação profissional ativa.

Não encontramos diferença na sintomatologia da AC quando comparamos o sexo, a idade, a situação conjugal e a situação profissional no dia do primeiro retorno após as cirurgias de CRM e correção cirúrgica de valvopatias.

No que diz respeito às correlações dos sintomas de AC com os sintomas de ansiedade e depressão nos pacientes submetidos à CRM, encontramos que quanto maior a percepção dos sintomas de AC no total, assim como no domínio "Medo e Hipervigilância", maior a percepção de ansiedade nos três tempos estudados. Não encontramos correlação dos sintomas de AC com os sintomas de depressão.

Quanto aos pacientes submetidos à cirurgias para correção de valvopatias, encontramos que quanto maior a percepção dos sintomas de AC no total, maior é a percepção de ansiedade no pré-operatório. Encontramos também correlação dos sintomas de AC com os sintomas de depressão, ou seja, quanto maior a percepção dos sintomas de AC no total, bem como no domínio "Medo e Hipervigilância", maior a sintomatologia de depressão no pré-operatório.

REFERÊNCIAS

ADAMS, J. et al. Importance of resistance training for patients after a cardiac event. **Baylor University Medical Center Proceedings**, v. 19, n. 3, p. 246-248, Jul. 2006. doi: 10.1080/08998280.2006.11928172.

ADHIKARI, G.; BARAL, D. Clinical profile of patients presenting with acute myocardial infarction. **Internacional Journal of Advances in Medicine**, v. 5, n. 2, p. 228-233, mar./abr. 2018. doi: 10.18203/23493933.ijam20181068.

AICHER, D. et al. Quality of life after aortic valve surgery: Replacement versus Reconstruction. **The Journal of Thoracic and Cardiovascular Surgery**, v. 142, n. 2, p. e19-e24, Ago. 2011. doi: 10.1016/j.jtcvs.2011.02.006.

AJZEN, I.; FISHBEIN, M. **Understanding attitudes and predicting social behavior**. New Jersey: Prentice-Hall, 1998, p. 97-99.

ALEXANDRI, A. et al. Factors associated with anxiety and depression in hospitalized patients with first episode of acute myocardial infarction. **Archives of Medical Science Atherosclerotic Diseases**, v. 2, p. e90-e99, Dec. 2017. doi: 10.5114/amsad.2017.72532.

AMERICAN HEART ASSOCIATION (AHA). **Coronary Artery Disease - Coronary Heart Disease**. 2020. Disponível em: <https://www.heart.org/en/health-topics/consumer-healthcare/what-is-cardiovascular-disease/coronary-artery-disease>. Acesso em: 15 fev. 2020.

ANDRADE, I. N. G. et al. Avaliação do EuroSCORE como preditor de mortalidade em cirurgia cardíaca valvar no Instituto do Coração de Pernambuco. **Brazilian Journal of Cardiovascular Surgery**, São José do Rio Preto, v. 25, n. 1, p. 11-18, Mar. 2010. doi:10.1590/S0102-76382010000100007.

ANJOS, D. B. M. et al. Influência das características sociodemográficas e clínicas no impacto da doença em valvopatias. **Revista Brasileira de Enfermagem**, Brasília, v. 69, n. 1, p. 40-46, jan./fev. 2016. doi:10.1590/0034-7167.2016690105i.

ARAÚJO, H. V. S. et al. Qualidade de vida de pacientes submetidos à cirurgia de revascularização do miocárdio. **Revista Brasileira de Enfermagem**, v. 70, n. 2, p. 273-281, mar./abr. 2017. doi: 10.1590/0034-7167-2016-0201.

ASSIS, C. C. et al. Acolhimento e sintomas de ansiedade em pacientes no pré-operatório de cirurgia cardíaca. **Revista Brasileira de Enfermagem**, Brasília, v. 67, n. 3, p. 401-407, maio/jun. 2014. doi:10.5935/0034-7167.20140053.

AUENSEN, A. et al. Morbidity outcomes after surgical aortic valve replacement. **Open Heart**, v. 4. n. 1, e000588, Abr. 2017. doi: 10.1136/openhrt-2017-000588.

AXELSSON, T. A. et al. Long-term outcomes after coronary artery bypass surgery in patients with diabetes. **Interactive CardioVascular and Thoracic Surgery**, v. 30, n.5, p. 685–690, Fev. 2020. doi: 10.1093/icvts/ivaa009.

BAGNATORI, R. S. et al. Síndromes coronárias agudas. In: QUILICI, A. P. et al. **Enfermagem em cardiologia**. 1a ed. São Paulo: Editora Atheneu, 2009. Cap. 19, p. 305-323.

BANO, T. et al. Immediate postoperative complications following coronary artery bypass grafting in patients with type 2 diabetes: A prospective cohort study. **Diabetes and Metabolic Syndrome: Clinical Research & Reviews**, v. 14, n. 1, p. 47-51, jan./fev. 2020. doi: 10.1016/j.dsx.2019.12.004.

BARBOSA, J. L. et al. Impacto dos Fatores de Risco para Doença Arterial Coronariana nos Gastos Hospitalares dos Pacientes Submetidos à Cirurgia de Revascularização do Miocárdio no SUS. **International Journal of Cardiovascular Sciences,** Rio de Janeiro, v. 31, n. 2, p. 90-96, Abr. 2018. doi: 10.5935/2359-4802.20170098.

BERGER, B.; STENSTRÖNM, G.; SUNDKVIST, G. Incidence, Prevalence, and Mortality of Diabetes in a Large Population. A report from the Skaraborg Diabetes Registry. **Diabetes Care,** v. 22, n. 5, p. 773-778, Maio. 1999. doi: 10.2337/diacare.22.5.773.

BLUMENTHAL, J. A. et al. Depression as a risk factor for mortality after coronary artery bypass surgery. **The Lancet**, v. 362, n. 9384, p. 604-609, Ago. 2003. doi:10.1016/S0140-6736(03)14190-6.

BOTEGA, N. J. et al. Transtornos do humor em enfermaria de clínica médica e validação de escala de medida (HAD) de ansiedade e depressão. **Revista de Saúde Pública**, São Paulo, v. 29, n. 5, p. 355-363, Out. 1995. doi: 10.1590/S0034-89101995000500004.

BOTZET, K. et al. Anxiety and Depression in Patients Undergoing Mitral Valve Surgery: A Prospective Clinical Study. **Thoracic and Cardiovascular Surgeon**, v. 66, n. 7, p. 530-536, Ago., 2018. doi: 10.1055/s-0037-1604461.

BRASIL. Ministério da Saúde. **Informações de Saúde. Informações epidemiológicas e morbidade**. Secretaria Executiva. Datasus. 2016. Disponível em: <http://www.datasus.gov.br>. Acesso em: 22 ago. 2016.

CABRERA, C. C.; SPONHOLZ JUNIOR, A. Ansiedade e insônia. In: BOTEGA, N. J. (Org.) **Prática psiquiátrica no hospital geral**. 3a ed. Porto Alegre: Artmed, 2012. Cap. 27, p. 411-429.

CALÇA, R. et al. Impacto do Implante Percutâneo de Válvula Aórtica na Função Renal. **Arquivos Brasileiros de Cardiologia**, São Paulo, v. 113, n. 6, p. 1104-1111, Dez. 2019 . doi: 10.36660/abc.20180356.

CALLES, A. C. N. et al. Pulmonary complications in patients undergoing coronary artery bypass grafting at a hospital in Maceio, Brazil. **Fisioterapia em Movimento**, v. 29, n. 4, p. 661-667, Dez. 2016. doi: 10.1590/19805918.029.004.ao01.

CAMPONOGARA, S. et al. Percepção de pacientes sobre o período pré-operatório de cirurgia cardíaca. **Revista Mineira de Enfermagem**, v. 16, n. 3, p. 382-390, jul./set. 2012. Disponível em: <http://www.reme.org.br/artigo/detalhes/541>. Acesso em: 29 jan. 2017.

CETINKAYA, A. et al. Long-term outcome after mitral valve replacement using biological versus mechanical valves. **Journal of Cardiothoracic Surgery**, v. 14, n. 1, p. 120, Jun. 2019. doi: 10.1186/s13019-019-0943-6.

CHEN, Y. et al. Symptoms, Hope, Self-Management Behaviors, and Quality of Life Among Chinese Preoperative Patient With Symptomatic Valvular Heart Diseases. **Journal of Transcultural Nursing**, v. 31, n. 3, p. 284-293, Jul. 2019. doi: 10.1177/1043659619864157.

CORDEIRO, A. L. L. et al. Aplicação do questionário de ansiedade cardíaca no pós-operatório de cirurgia cardíaca. **Revista Brasileira de Prescrição e Fisiologia do Exercício**, v. 9, n. 56, p. 592-596, 2015. Disponível em: <http://www.rbpfex.com.br/index.php/rbpfex/article/view/812>. Acesso em: 12 mar. 2020.

CORTEZ, C. M.; SILVA, D. Implicações do estresse sobre a saúde e a doença mental. **Arquivos Catarinenses de Medicina**, v. 36, n. 4, p. 96-108, out./dez. 2007. Disponível em: <https://pesquisa.bvsalud.org/portal/resource/pt/lil-479394>. Acesso em: 01 set. 2020.

COSTA, R. L. et al. Impacto da Hipertensão Pulmonar na Mortalidade Cirúrgica e na Sobrevida em Três Anos Pós-Cirurgia de Troca Valvar Aórtica. **International Journal of Cardiovascular Sciences**, v. 29, n. 1, p. 24-30, 2016. doi:10.5935/2359-4802.20160010.

CSERÉP, Z. et al. The impact of preoperative anxiety and education level on long-term mortality after cardiac surgery. **Journal of Cardiothoracic Surgery**, v. 7, 2012. doi: 10.1186/1749-8090-7-86.

CUNHA, M. N. F. **Desenvolvimento e validação preliminar de um instrumento breve para medir o estresse psicológico pré-operatório.** 2014. 100f. Tese (Doutorado em Medicina) - Faculdade de medicina, Universidade Federal do Rio Grande do Sul, Porto Alegre, 2014.

DE LIMA JÚNIOR, J. D.; MATIAS, J. E. F.; STAHLKE JÚNIOR, H. J. Risk factors associated with hospital mortality in mitral valve reoperation. **Revista do Colégio Brasileiro**

de Cirurgiões, Rio de Janeiro, v. 46, n. 3, p. e20192176, 2019. doi: 10.1590/0100-6991e-20192176.

DORDETTO, P. R.; PINTO, G. C.; ROSA, T. C. S. C. Pacientes submetidos à cirurgia cardíaca: caracterização sociodemográfica, perfil clínico-epidemiológico e complicações. **Revista da Faculdade de Ciências Médicas de Sorocaba**, v. 18, n. 3, p. 144-149, Set. 2016. doi: 10.5327/Z1984-4840201625868.

EIFERT, G. H. et al. The Cardiac Anxiety Questionnaire: development and preliminary validity. **Behaviour Research and Therapy**, v.38, n.10, p.1039-1053, Out. 2000. doi: 10.1016/s0005-7967(99)00132-1.

EIFERT, G.H. Cardiophobia: a paradigmatic behavioural model of heart-focused anxiety and non-anginal chest pain. **Behaviour Research and Therapy**, v.30, n.4, p.329-345, 1992. doi: 10.1016/0005-7967(92)90045-I.

FENG, H. et al. Risk of anxiety and depressive disorders in patients with myocardial infarction. **Medicine (Baltimore)**, v. 95, n. 34, p. e4464, Ago. 2016. doi: 10.1097/MD.0000000000004464.

FISCHER, D. et al. Heart-focused anxiety in the general population. **Clinical Research in Cardiology**, v. 101, n. 2, p. 109-116, Fev. 2012. doi:10.1007/s00392-011-0371-7.

FLORENTINO, T. M. et al. Evolução da insuficiência mitral primária em pacientes com estenose aórtica grave 1 ano após implante transcateter de valva aórtica: avaliação ecocardiográfica evolutiva. **Arquivos Brasileiros de Cardiologia**, São Paulo , v. 109, n. 2, p. 148-155, Ago. 2017. doi: 10.5935/abc.20170094.

FORMENTINI, F. S. et al. Coronary artery disease and body mass index: What is the relationship? **Clinical Nutrition ESPEN**, v. 34, p. 87-93, Dez. 2019. doi: 10.1016/j.clnesp.2019.08.008.

FU, J. et al. Outcomes of mitral valve repair compared with replacement for patients with rheumatic heart disease. **The Journal of Thoracic and Cardiovascular Surgery**, Fev. 2020. doi: 10.1016/j.jtcvs.2020.01.053.

FUKUNAGA, N.; SAKATA, R.; KOYAMA, T. Short- and long-term outcomes following redo valvular surgery. **Journal of Cardiac Surgery**, v. 33, n. 2, p. 56-63, Fev. 2018. doi: 10.1111/jocs.13534.

GIARETTA, D. G. et al. Qualidade de vida e resiliência em pacientes pós-operatório de cirurgia cardíaca. **Revista de Atenção à Saúde**, São Caetano do Sul, v. 16, n. 58, p. 5-11, out./dez., 2018. doi: 10.13037/ras.vol16n58.5399.

GLASER, R.; KIECOLT-GLASER, J. K. Stress-induced immune dysfunction: implications for health. **Nature Reviews Immunology**, v. 5, n. 3, p. 243-251, Mar. 2005. doi: 10.1038/nri1571.

GONÇALVES, T. F; MEDEIROS, V. C. C. The preoperative visit as the anxiety mitigating factor in surgical patients. **Revista Sobecc**, São Paulo, v. 21, n. 1, p. 22-27, jan./mar. 2016. doi: 10.5327/Z1414-4425201600010004.

GUEDENEY, P. et al. Incidence of, risk factors for and impact of readmission for heart failure after successful transcatheter aortic valve implantation. **Archives of Cardiovascular Disease**, v. 112, n.12, p. 765-772, Nov. 2019. doi: 10.1016/j.acvd.2019.09.008.

GUO, P. Preoperative education interventions to reduce anxiety and improve recovery among cardiac surgery patients: a review of randomised controlled trials. **Journal of Clinical Nursing**, v. 24, n. 1-2, p. 34-46, Jan. 2015. doi:10.1111/jocn.12618.

GUYTON, A. C.; HALL, J. E. **Tratado de fisiologia médica**. 12a ed. Rio de Janeiro: Elsevier, 2011, 1176 p.

HOHLS, J. K. et al. Association between heart-focused anxiety, depressive symptoms, health behaviors and healthcare utilization in patients with coronary heart disease. **Journal of Psychosomatic Research**, v. 131, p. 109958. 2020. doi: 10.1016/j.jpsychores.2020.109958.

HOYER, J. et al. Heart-focused anxiety before and after cardiac surgery. **Journal of Psychosomatic Research**, v. 64, n. 3, p. 291-297, Mar. 2008. doi:10.1016/j.jpsychores.2007.09.009.

HULLEY, S.B.; CUMMINGS, S.R.; NEWMAN, T.B. Delineando estudos transversais e de coorte. In: HULLEY, S.B.; CUMMINGS, S.R.; BROWNER, W.S.; GRDY, D.G.; NEWMAN, T.B. **Delineando a pesquisa clínica**. 4a ed. Porto Alegre: Artmed, 2015. Cap.7, p. 90-102.

HUSSAIN, A. I. et al. Age-dependent morbidity and mortality outcomes after surgical aortic valve replacement. **Interactive CardioVascular and Thoracic Surgery**, v. 27, n. 5, p. 650-656, Nov. 2018. doi: 10.1093/icvts/ivy154.

JANSSEN, A. M. S. et al. Perfil sociodemográfico e clínico de pacientes submetidos à cirurgia de revascularização do miocárdio. **Revista de Pesquisa em Saúde**, v. 16, n. 1, p. 29-23, jan./abri. 2015. Disponível em: < http://www.periodicoseletronicos.ufma.br/index.php/revistahuufma/article/view/4073/2155>. Acesso em: 15 de jan. 2020.

JONHSON, L. R. **Essencial Medical Physiology**. 3. ed. Philadelphia: Academic Press. 1998.

KAFADAR, D. et al. Association of Sociodemographic Profile, Dyslipidemias, and Obesity in Smoker, Former Smoker, and Nonsmoker Patients with Coronary Artery Disease. **Nigerian Journal of Clinical Practice**, v. 21, n. 9, p. 1190-1197, Ago. 2018. doi: 10.4103/njcp.njcp_171_17.

KALOGIANNI, A. et al. Can nurse-led preoperative education reduce anxiety and postoperative complications of patients undergoing cardiac surgery? **European Journal of Cardiovascular Nursing**, v. 15, n. 6, p. 447-458, Out. 2016. doi:10.1177/1474515115602678.

KAPADIA, M. et al. Cognition, Frailty, and Functional Outcomes of Transcatheter Aortic Valve Replacement. **The American Jornal of Medicine**, 2020. doi: 10.1016/j.amjmed.2020.01.041.

KSIAZEK, K. et al. Premature senescence of mesothelial cells is associated with non-telomeric DNA damage. **Biochemical and Biophysical Research Communications**, v. 362, n. 3, p. 707-711, Out. 2007. doi:10.1016/j.bbrc.2007.08.047.

KAZITANI, B. S. et al. Preoperative anxiety and depression: differences among patients submitted to the first cardiac surgery. **Revista da Rede de Enfermagem do Nordeste**, v.19, p.e3079, 2018. doi: 10.15253/2175-6783.2018193079.

KELPIS, T. G. et al. Prevalence of "distressed" personality in patients with coronary artery disease and its correlation with morbidity after coronary surgery. **Hellenic Journal of Cardiology**, v. 54, n. 5, p. 362-367, set./out. 2013. Disponível em: <http://www.hellenicjcardiol.org/archive/full_text/2013/5/2013_5_362.pdf>. Acesso em: 29 jan. 2017.

KOERICH, C. et al. Revascularização miocárdica: estratégias para o enfrentamento da doença e do processo cirúrgico. **Acta Paulista de Enfermagem**, São Paulo, v. 26, n. 1, p. 8-13, 2013. doi: 10.1590/S0103-21002013000100003.

KRANNICH, J. H. A. et al. Presence of depression and anxiety before and after coronary artery bypass graft surgery and their relationship to age. **BMC Psychiatry**, v. 47, n. 7, Set. 2007. doi:10.1186/1471-244X-7-47.

KRZEMIŃSKA, S. et al. Socio-demographic and clinical predictors of health-related quality of life in patients after myocardial infarction. **Journal of Pre-Clinical and Clinical Research**, v. 13, n. 1, p. 13-18, Mar. 2019. doi: 10.26444/jpccr/102758.

KSHIRSAGAR, D. et al. Pulmonary Function Tests in Patients Undergoing Coronary Artery Bypass Graft Surgery and its Correlation with Outcome. **Journal of The Association of Physicians of India**, v. 68, n. 2, p. 39-42, Fev. 2020. Disponível em: < https://www.ncbi.nlm.nih.gov/pubmed/32009361>. Acesso em: 20 mar. 2020.

KWEDAR, K. et al. Outcomes of Early Mitral Valve Reoperation in the Medicare Population. **The Annals of Thoracic Surgery**, v. 104, n. 5, p. 1516-1521, Jul. 2017. doi: 10.1016/j.athoracsur.2017.05.001.

LARAIA, M. T. Psicofarmacologia. IN: STUART, G. W.; LARAIA, M. T. **Enfermagem Psiquiátrica**: Princípios e prática. 6a ed. Porto Alegre: Artmed, 2001. Cap, 27. p 607 – 639.

LI, R. L. et al. Heart valve operations associated with reduced risk of death from mitral valve disease but other operations associated with increased risk of death: a national population-based case–control study. **Journal of Cardiothoracic Surgery**, v. 14, n. 1, p. 165, Set. 2019. doi: 10.1186/s13019-019-0984-x.

LIN, Y. S. et al. Predicting exercise capacity recovery immediately after mitral valve surgery. **Journal of Cardiac Surgery**, v. 34, n. 10, p. 889-894, Out. 2019. doi: 10.1111/jocs.14131.

LOTUFO, P. A. Doenças cardiovasculares no mundo. In: MAGALHÃES, C. C. et al. **Tratado de cardiologia SOCESP**. 3 ed. Barueri: Manole, 2015. Seção 1, Cap. 1, p. 2-10.

LUENG, K.; MUNCK, A. Peripheral actions of glucocorticoids. **Annual Review of Physiology**, v. 37, p. 245-272, Maio. 1975. doi: 10.1146/annurev.ph.37.030175.001333.

MAENG, L. Y.; MILAND, M. R. Post-Traumatic Stress Disorder: The Relationship Between the Fear Response and Chronic Stress. **Chronic Stress (Thousand Oaks)**, v. 1, Jun. 2017. doi: 10.1177/2470547017713297.

MARKER, C. D.; CARMIN, C. N.; OWNBY, R. L. Cardiac Anxiety in people with and without Coronary Atherosclerosis. **Depress Anxiety**, v. 25, n. 10, p. 824-831, Nov. 2008. doi: 10.1002/da.20348.

MARTINS, A. B. B. **Avaliação do desempenho de escores de prognóstico de cirurgia cardíaca em pacientes submetidos à troca valvar por endocardite infecciosa no Instituto Nacional de Cardiologia, anos de 2006 a 2016.** 2016. 74 p. Dissertação (Mestrado) - Instituto Nacional de Cardiologia, Rio de Janeiro, 2016.

MATHEVON, T. et al. Acute abdominal dilatation, a serious complication in the case of anorexia nervosa. **La Presse Médicale**, v. 33, n. 9, p. 601-603, Mai. 2004. doi: 10.1016/s0755-4982(04)98684-8.

MCEWEN, B.S.; GIANAROS, P.J. Central role of the brain in stress and adaptation: links o socioeconomic status, health, and disease. **Annals of the New York Academy of Sciences**, v. 1186, p. 190–222. Fev. 2010. doi: 10.1111/j.1749-6632.2009.05331.x

MCEWEN, B. S. Central effects of stress hormones in health and disease: Understanding the protective and damaging effects of stress and stress mediators. **European Journal of Pharmacology**, v. 583, n. 2–3, p. 174–185, Abri. 2008. doi: 10.1016/j.ejphar.2007.11.071.

MCEWEN, B. S. Mood disorders and allostatic load. **Biological Psychiatry**, v. 54, n. 3, p. 200–207, Ago. 2003. doi: 10.1016/s0006-3223(03)00177-x.

MERTINS, S. M. et al. Prevalência de fatores de risco em pacientes com infarto agudo do miocárdio. **Avances en Enfermería**, v. 34, n. 1, p. 30-38, Jan. 2016. doi: 10.15446/av.enferm.v34n1.37125.

MILLER, G.E.; CHEN, E.; ZHOU, E. S. If it goes up, must it come down? Chronic stress and the hypothalamic-pituitary-adrenocortical axis in humans. **Psychological Bulletin**, v. 133, n. 1, p. 25-45, Jan. 2007. doi: 10.1037/0033-2909.133.1.25.

MITRA, R. et al. Stress duration modulates the spatiotemporal patterns of spine formation in the basolateral amygdala. **Proceedings of the National Academy of Sciences**, v. 102, n. 26, p. 9371-9376, Jun. 2005. doi: 10.1073/pnas.0504011102

MODICA, M. et al. Psychological Profile in Coronary Artery By-Pass Graft Patients vs. Valve Replacement Patients Entering Cardiac Rehabilitation after Surgery. **Scientific Reports**, Set. 2018. doi: 10.1038/s41598-018-32696-5.

MONTELEONE, P. et al. Circulating brain-derived neurotrophic factor in anorexia and bulimia nervosa but not in women with binge-eating disorder: relationships to co-morbid depression, psychopathology and hormonal variables. **Psychological Medicine**, v. 35, n. 6, p. 897-905, Jun. 2005. doi: 10.1017/s0033291704003368.

MORAES, R. C. S. **Validação do EuroSCORE em valvopatas submetidos à cirurgia cardíaca.** 2013. 88 p. Tese (Doutorado) - Faculdade de Medicina, Universidade de São Paulo. São Paulo, 2013.

MORAES, V. C. S. et al. Avaliação da ansiedade cardíaca no pós-operatório de cirurgia cardíaca. **Revista Brasileira de Neurologia e Psiquiatria**, v. 7, n. 3, p. 91-102. Dez. 2013. Disponível em: < https://www.revneuropsiq.com.br/rbnp/article/view/24>. Acesso em: 5 jan. 2020.

MORENO-PERAL, P. et al. Risk factors for the onset of panic and generalised anxiety disorders in the general adult population: A systematic review of cohort studies. **Journal of Affective Disorders**, v. 168, p. 337-348, Out. 2014. doi: 10.1016/j.jad.2014.06.021.

MOURAD, G. et al. Depressive Symptoms, Cardiac Anxiety, and Fear of Body Sensations in Patients with Non-Cardiac Chest Pain, and Their Relation to Healthcare-Seeking Behavior: A

Cross-Sectional Study. **The Patient - Patient-Centered Outcomes Research**, v. 9, n. 1, p. 69-77, Fev. 2016. doi: 10.1007/s40271-015-0125-0.

MURPHY, B. et al. Anxiety and Depression After a Cardiac Event: Prevalence and Predictors. **Frontiers in Psychology**, v. 10. Jan. 2020. doi: 10.3389/fpsyg.2019.03010.

MYIN-GERMEYS, I. et al. Emotional reactivity to daily life stress in psychosis. **Archives of General Psychiatry**, v. 58, n. 12, p. 1137-1144, Dez. 2001. doi: 10.1001/archpsyc.58.12.1137.

NASSER, F. J. et al. Doenças psiquiátricas e o sistema cardiovascular: interação cérebro e coração. **International Journal of Cardiovascular Sciences**, v. 29, n. 1, p. 65-75, jan./fev. 2016. doi: 10.5935/2359-4802.20160003.

NIELSEN, S. et al. Social Factors, Sex, and Mortality Risk After Coronary Artery Bypass Grafting: A Population-Based Cohort Study. **Journal of the American Heart Association**, v. 8, n. 6, Mar. 2019. doi: 10.1161/JAHA.118.011490.

OLIVEIRA, G. S. **Análise da coagulação sanguínea com a administração profilática da desmopressina em cirurgias cardíacas valvares**. 2017. 125 p. Tese (Doutorado) - Faculdade de Medicina, São Paulo, 2017.

ORGANIZAÇÃO PAN-ANMERICANA DE SAÚDE. OPAS/OMS Brasil, 2020. **Doenças Cardiovasculares**. Disponível em: <https://www.paho.org/bra/index.php?option=com_content&view=article&id=5253:doencas-cardiovasculares&Itemid=1096>. Acesso em: 15 fev. 2020.

ÖSTGREN, C. J. et al. Survival in Patients With Type 2 Diabetes in a Swedish Community. **Diabetes Care**, v. 25, n. 8, p. 1297-1302, Ago. 2002. 10.2337/diacare.25.8.1297.

OTERHALS, K. et al. Factors associated with poor self-reported health status after aortic valve replacement with or without concomitant bypass surgery. **European Journal of Cardio-Thoracic Surgery**, v. 48, n. 2, p. 283-292, Ago. 2015. doi: 10.1093/ejcts/ezu425.

PACARIC, S. et al. Assessment of the Quality of Life in Patients before and after Coronary Artery Bypass Grafting (CABG): A Prospective Study. **International Journal of Environmental Research and Public Health**, v. 17, n. 4, Fev. 2020. doi: 10.3390/ijerph17041417.

PEREIRA, V. R. **A Cronicidade da Valvopatia:** Aspectos psicológicos na escolha da prótese valvular. 2019. 89 p. Dissertação (Mestrado) - Universidade Fernando Pessoa, Porto, 2019.

PERROTTI, A. et al. Quality of life 10 years after cardiac surgery in adults: a long-term follow-up study. **Health and Quality of Life Outcomes**, v. 17, n. 1, Maio. 2019. doi: 10.1186/s12955-019-1160-7.

PETERSEN, J. et al. Physical and mental recovery after conventional aortic valve surgery. **The Journal of Thoracic and Cardiovascular Surgery**, v. 152, n. 6, p. 1549-1556, Dez. 2016. doi: 10.1016/j.jtcvs.2016.07.072.

PFEIFFER, E. A Short Portable Mental Status Questionnaire for the Assessment of Organic Brain Deficit in Elderly Patients. **Journal of the American Geriatrics Society**, v. 23, n. 10, p. 433-441, Out. 1975. doi:10.1111/j.1532-5415.1975.tb00927.x.

PINTON, F. A. et al. Depressão como fator de risco de morbidade imediata e tardia pós-revascularização cirúrgica do miocárdio. **Brazilian Journal of Cardiovascular Surgery**, São José do Rio Preto, v. 21, n. 1, p. 68-74, Mar. 2006. doi: 10.1590/S0102-76382006000100013.

POOLE, L. et al. Pre-surgical depression and anxiety and recovery following coronary artery bypass graft surgery. **Journal of Behavioral Medicine**, v. 40, n. 2, p. 249-258, abr. 2017. doi:10.1007/s10865-016-9775-1.

POOLE, L. et al. The combined association of depression and socioeconomic status with length of post-operative hospital stay following coronary artery bypass graft surgery: Data from a prospective cohort study. **Journal of Psychosomatic Research**, v. 76, n. 1, p. 34-40, Jan. 2014. doi: 10.1016/j.jpsychores.2013.10.019.

PRADO-OLIVARES, J.; CHOVER-SIERRA, E. Preoperatory Anxiety in Patients Undergoing Cardiac Surgery. **Diseases**, v. 7, n. 2, p. 46, Jun. 2019. doi: 10.3390/diseases7020046.

RAMESH, C. et al. Pre-operative anxiety in patients undergoing coronary artery bypassgraft surgery – A cross-sectional study. **International Journal of Africa Nursing Sciences**, v. 7, p. 31-36, Jun. 2017. doi: 10.1016/j.ijans.2017.06.003.

REGGI, S.; STEFANINI, E.; CARVALHO, A. C. C. Diagnóstico e decisão terapêutica na doença coronariana crônica. In: MAGALHÃES, C. C. et al. **Tratado de cardiologia SOCESP**. Barueri: Manole, 3 ed., 2015. 526-535.

RICE, V. H. **Handbook of stress, coping and health: implications for nursing research, theory and practice**. 2nd ed. Los Angeles: SAGE, 2012, p. 624.

RODENBECK, A.; HAJAK, G. Neuroendocrine dysregulation in primary insomnia. **Revue Neurologique**, v. 157, n. 11, p. S57-61, Nov. 2001. Disponível em: <https://pubmed.ncbi.nlm.nih.gov/11924040/>. Acesso em: 23 ago. 2020.

RODRIGUES, A. J. et al. Fatores de risco para lesão renal aguda após cirurgia cardíaca. **Brazilian Journal of Cardiovascular Surgery**, São José do Rio Preto, v. 24, n. 4, p. 441-446, out./dez. 2009. doi: 10.1590/S0102-76382009000500003.

RODRIGUES, H. F. et al. Relationship between emotional states before cardiac valve surgeries with postoperative complications. **Revista Gaúcha de Enfermagem**, v. 41, p. e20190025, 2020. doi: 10.1590/1983-1447.2020.20190025.

RODRIGUES, H. F. et al. Association of preoperative anxiety and depression symptoms with postoperative complications of cardiac surgeries. **Revista Latino-Americana de Enfermagem**, v. 26, p. e3107, Nov. 2018. doi:10.1590/1518-8345.2784.3107.

RYMASZEWSKA, J.; KIEJNA, A.; HADRYS, T. Depression and anxiety in coronary artery bypass grafting patients. **European Psychiatry**, v. 18, n. 4, p. 155-160, Jun. 2003. doi:10.1016/s0924-9338(03)00052-x.

SANTOS, A. R. et al. Relação entre qualidade de vida e impacto da doença em valvopatas com diferentes graus de comprometimento ventricular. **Assobrafir Ciência**, v. 9, n. 1, p. 23-32, Abr., 2018. Disponível em: < http://www.uel.br/revistas/uel/index.php/rebrafis/article/view/27923>. Acesso em: 13 abr. 2020.

SAPOLSKY, R. M.; ROMERO, L. M.; MUNCK. A. U. How do glucocorticoids influence stress responses? Integrating permissive, suppressive, stimulatory, and preparative actions. **Endocrine Reviews**, v. 21, n. 1, p. 55–89. Fev. 2000. doi: 10.1210/edrv.21.1.0389.

SARAIVA, J. F. K.; GAGLIARDI, S. P. L. Doenças cardiovasculares no Brasil. In: MAGALHÃES, C. C. et al. **Tratado de cardiologia SOCESP**. 3a ed. Barueri: Manole, 2015. Seção 1, Cap. 2, p. 11-22.

SARDINHA, A. et al. Prevalência de transtornos psiquiátricos e ansiedade relacionada à saúde em coronariopatas participantes de um programa de exercício supervisionado. **Revista de Psiquiatria Clínica**, São Paulo, v. 38, n. 2, p. 61-65, 2011. doi: 10.1590/S010160832011000200004.

SARDINHA, A.; ARAUJO, C. G. S.; NARDI, A. E. Psychiatric disorders and cardiac anxiety in exercising and sedentary coronary artery disease patients: a case-control study. **Brazilian Journal of Medical and Biological Research**, v. 45, n. 12, p. 1320-1326, Dez. 2012. doi: 10.1590/S0100-879X2012007500156.

SARDINHA, A. et al. Validação da versão Brasileira do Questionário de Ansiedade Cardíaca. **Arquivos Brasileiros de Cardiologia**, v. 101, n. 6, p. 554-561, Dez. 2013. doi:10.5935/abc.20130207.

SARDINHA, A; NARDI, A.E.; EIFERT, G.H. Tradução e Adaptação transcultural da Versão Brasileira do Questionário de Ansiedade Cardíaca. **Revista de Psiquiatria do Rio Grande do Sul**, v.30, n.2, p.139-149, 2008. doi: 10.1590/S0101-81082008000300010.

SCHLYTER, M. et al. Smoking Cessation After Acute Myocardial Infarction in Relationto Depression and Personality Factors. **International Society of Behavioral Medicine**, v. 23, n. 2, p. 234-242, Abr. 2016. doi: 10.1007/s12529-015-9514-y.

SEILER, A.; FAGUNDES, C. P.; CHRISTIAN, L. M. The Impact of Everyday Stressors on the Immune System and Health. In: CHOUKÈR, A. (Org.). **Stress Challenges and Immunity in Space**. Berlin: Spring, 2011, p. 31-43.

SELYE, H. The general adaptation syndrome and the diseases of adaptation. **The Journal of Clinical Endocrinology and Metabolism**. v. 6, n. 2, p. 117-130, Fev. 1946. doi:10.1210/jcem-6-2-117.

SILVA, F. L.; MELO, M. A. B.; NEVES, R. A. et al. Perfil clínico-epidemiológico dos pacientes internados por infarto agudo do miocárdio em hospital de Goiás. **Revista Brasileira Militar de Ciências**, v. 5, n. 13, Dez. 2019. doi: 10.36414/rbmc.v5i13.15.

SILVA, L. D. C. et al. Intervenções de enfermagem em pacientes da unidade de terapia intensiva cardiológica de um hospital universitário submetidos à cirurgia de revascularização do miocárdio. **Journal of Management & Primary Health Care**, v. 9, Dez. 2018. doi: 10.14295/jmphc.v9i0.510.

SILVA, L. N. **Comparação de três instrumentos para avaliação da fadiga em pacientes com insuficiência cardíaca**. 2016. 99 f. Dissertação (Mestrado) - Escola de Enfermagem de Ribeirão Preto da Universidade de São Paulo, Ribeirão Preto, 2016.

SILVA, L. N. et al. Perfil epidemiológico e clínico de pacientes com síndrome coronariana aguda. **Revista de Enfermagem UFPE online**, v. 12, n. 2, p. 379-385, Fev. 2018. doi: 10.5205/1981-8963-v12i2a22563p379-385-2018.

SOUSA, A. G. et al. Epidemiologia da cirurgia de revascularização miocárdica do Hospital Beneficência Portuguesa de São Paulo. **Revista Brasileira de Cirurgia Cardiovascular**, v. 30, n. 1, p. 33-39, Fev. 2015. doi: 10.5935/1678-9741.20140062.

STANSFELD, S. A. et al. Psychological distress as a risk factor for coronary heart disease in the Whitehall II Study. **International Journal of Epidemiology**, v. 31, n. 1, p. 248-255, Fev. 2002. doi: 10.1093/ije/31.1.248

STENMAN, M.; HOLZMANN, M. J.; SARTIPY, U. Antidepressant use before coronary artery by-pass surgery is associated with long-term mortality. **International Journal of Cardiology**, v. 167, n. 6, p. 2958-2962, Set. 10, 2013. doi: 10.1016/j.ijcard.2012.08.010.

SZCZEPANSKA-GIERACHA, J. et al. The role of depressive and anxiety symptoms in the evaluation of cardiac rehabilitation efficacy after coronary artery bypass grafting surgery. **European Journal of Cardio-Thoracic Surgery**, v. 42, n. 5, p. e108-e114, Nov. 2012. doi: 10.1093/ejcts/ezs463.

TARASOUTCHI, F. et al. Diretriz Brasileira de Valvopatias - SBC 2011 / I Diretriz Interamericana de Valvopatias - SIAC 2011. **Arquivos Brasileiros de Cardiologia**, São Paulo, v. 97, n. 5 supl. 1, p. 1-67, Nov. 2011. doi: 0.1590/50066-782X2011002000001.

TORRATI, F. G. **Ansiedade, depressão, senso de coerência e estressores nos períodos pré e pós-operatório de cirurgias cardíacas**. 2009. 110 f. Dissertação (Mestrado em Enfermagem Fundamental) – Escola de Enfermagem de Ribeirão Preto, Universidade de São Paulo, Ribeirão Preto, 2009.

TREMBLAY, M. A. et al. Heart-focused anxiety and health care seeking in patients with non-cardiac chest pain: A prospective study. **General Hospital Psychiatry**, v. 50, p. 83-89, jan./fev. 2018. doi: 10.1016/j.genhosppsych.2017.10.007.

TULLY, P. J.; BAKER, R. A. Depression, anxiety, and cardiac morbidity outcomes after coronary artery bypass surgery: a contemporary and practical review. **Journal of Geriatric Cardiology**, v. 9, n. 2, p. 197-208, Jun. 2012. doi:10.3724/SP.J.1263.2011.12221.

TULLY, P. J.; NEWLAND, R. F.; BAKER, R. A. Cardiovascular risk profile before coronary artery bypass graft surgery in relation to depression and anxiety disorders: an age and sex propensity matched study. **Australian Critical Care**, v. 28, n. 1, p. 24-30, Fev. 2015. doi:10.1016/j.aucc.2014.04.006.

VAN BEEK, et al. Prognostic association of cardiac anxiety with new cardiac events and mortality following myocardial infarction. **The British Journal of Psychiatry**, v. 209, n. 5, p. 400-406, Nov. 2016. doi: 10.1192/bjp.bp.115.174870.

VAN DER KOOY, K. et al. Depression and the risk for cardiovascular diseases: systematic review and meta analysis. **International Journal of Geriatric Psychiatry**, v. 22, n. 7, p. 613-623, Jul. 2007. doi: 10.1002/gps.1723.

WHITE, K. S.; CRAFT, J. M.; GERVINO, E. V. Anxiety and hypervigilance to cardiopulmonary sensations in non-cardiac chest pain patients with and without psychiatric disorders. **Behaviour Research and Therapy**, v. 48, n. 5, p. 394-401, Maio. 2010. doi: 10.1016/j.brat.2010.01.001.

WILLIAMS, J. B. et al. Preoperative anxiety as a predictor of mortality and major morbidity in patients aged >70 years undergoing cardiac surgery. **American Journal of Cardiology**, v. 111, n. 1, p. 137-142, Jan. 2013. doi:10.1016/j.amjcard.2012.08.060.

YAFTIAN, N. et al. Long-term outcomes of mitral valve endocarditis: improved survival through collaborative management. **ANZ Journal of Surgery**, v.90, n. 5, Mar. 2020. doi: 10.1111/ans.15814.

YANG, L. X. et al. Differential Impact of Cigarette Smoking on Prognosis in Women and Men Undergoing Percutaneous Coronary Intervention. **Angiology**, v. 71, n. 3, p. 281-287, Mar. 2020. doi: 10.1177/0003319719889276.

ZIGMOND, A. S.; SNAITH, R. P. The hospital anxiety and depression scale. **Acta Psychiatrica Scandinavica**, v. 67, n. 6, p. 361-370, Jun. 1983. doi:10.1111/j.1600-0447.1983.tb09716.x.

ZORN, J. V. et al. Cortisol stress reactivity across psychiatric disorders: A systematic review and meta-analysis. **Psychoneuroendocrinology**, v. 77, p. 25-36, Mar. 2017. doi: 10.1016/j.psyneuen.2016.11.036.

APÊNDICES

APÊNDICE A – TERMO DE CONSENTIMENTO LIVRE ESCLARECIDO - PACIENTES

Gostaríamos de convidar o (a) senhor (a) para participar, como voluntário (a), em uma pesquisa. Após ser esclarecido (a) sobre as informações a seguir, no caso de aceitar fazer parte do estudo, assine ao final deste documento, que está em duas vias que também serão assinadas pela pesquisadora. Uma delas é do (a) senhor (a) e a outra é da pesquisadora. O (a) senhor (a) não é obrigado (a) a participar. Em caso de dúvidas éticas, o (a) senhor (a) pode procurar o Comitê de Ética em Pesquisa da Escola de Enfermagem de Ribeirão Preto da Universidade de São Paulo pelo telefone (16) 3315-9197, de segunda à sexta-feira das 10h – 12h e das 14h – 16h. O CEP-EERP-USP tem como finalidade proteger eticamente o participante de projetos de pesquisa que envolvam seres humanos.

Título do Projeto: "Comparação dos sintomas de ansiedade, depressão e ansiedade cardíaca em pacientes no pré e pós-operatório de cirurgias cardíacas"

Pesquisadora responsável:

Professora Doutora da Escola de Enfermagem de Ribeirão Preto – USP, Carina Aparecida Marosti Dessotte; Celular: (16) 9776-7665; telefone: (16) 3315-3410; Endereço: Avenida dos Bandeirantes, 3900, Campus Universitário, Bairro Monte Alegre, CEP: 14.040-902, Ribeirão Preto/SP.

Pesquisadores colaboradores:

Bruna Sonego Kazitani. Enfermeira. Telefone: (16) 99145-8358.

Esta pesquisa pretende estudar a presença de sintomas de ansiedade cardíaca, ansiedade e depressão antes da cirurgia do coração, no dia da alta hospitalar e no dia do primeiro retorno após a alta hospitalar. Depois dessas três avaliações, vamos comparar os sintomas relatados pelo (a) senhor (a) para ver se eles permaneceram da mesma forma ou de maneira diferente durante o recurso da cirurgia.

Caso aceite participar, um dia antes da cirurgia nós vamos aplicar um breve questionário para avaliarmos se o (a) senhor (a) apresenta as características necessárias para participar desta pesquisa. Se o (a) senhor (a) não apresentar as características necessárias, a sua participação será encerrada neste momento, não sendo necessário responder os demais questionários. Mas no caso do (a) senhor (a) apresentar as características necessárias, iremos pedir para que o (a) senhor (a) responda um questionário sobre dados pessoais de sua vida e da história da doença, assim como vamos pedir para o (a) senhor(a) responder outros dois questionários: um que investiga a presença de sintomas de ansiedade cardíaca e outro que investiga a presença de sintomas de ansiedade e depressão. É esperado que o (a) senhor (a) leve em torno de 40 minutos para responder todas as perguntas. No dia da alta hospitalar vamos pedir para o senhor (a) responder novamente o questionário da ansiedade cardíaca e o questionário de ansiedade e depressão. É esperado que o (a) senhor (a) leve em torno de 20 minutos para responder todas as perguntas. Além disso, gostaríamos também de solicitar a sua autorização para analisar os dados de sua cirurgia e de sua permanência na enfermaria, por meio de consulta aos prontuários. Após um mês da alta hospitalar, o (a) senhor (a) retornará ao HC para a primeira consulta no ambulatório, nesse dia, vamos pedir para o senhor (a) responder novamente o questionário da ansiedade cardíaca e o questionário de ansiedade e depressão. É esperado que o (a) senhor (a) leve em torno de 20 minutos para responder todas as perguntas.

Sua participação nesta pesquisa não trará gastos financeiros e todas as suas dúvidas sobre a pesquisa poderão ser esclarecidas a qualquer momento pelos pesquisadores, nos telefones citados acima. Teremos o compromisso de que o (a) senhor (a) será devidamente orientado (a) com relação ao tema, se isso for do seu interesse.

Não é esperado que o (a) senhor (a) receba benefícios diretos pela participação nesta pesquisa, entretanto, acreditamos que com este estudo poderemos planejar com melhor qualidade a assistência de enfermagem prestada aos pacientes que farão cirurgias no coração.

Os riscos esperados pela sua participação nesta pesquisa podem estar relacionados com a possibilidade do (a) senhor (a) sentir-se triste, ansioso ou desconfortável com perguntas de cunho pessoal, contidas nos questionários. Se o (a) senhor (a) se sentir triste ou ansioso ao responder as perguntas, estaremos ao seu lado para te ouvir e dar todo o apoio necessário, entretanto, se isso não for suficiente, entraremos em contato com a equipe médica para que eles te avaliem. Além disso, garantimos que o (a) senhor (a) terá total liberdade para aceitar ou não participar desta pesquisa e de deixar de participar a qualquer momento sem precisar se justificar, retirando seu consentimento em qualquer fase do estudo, sem ser prejudicado (a) no seu atendimento no Hospital das Clínicas e nem nenhuma outra forma de prejuízo.

Os resultados do estudo serão utilizados para fins científicos e serão divulgados em eventos, revistas e meios de comunicação, no entanto, o (a) senhor (a) terá a segurança de não ser identificado (a) em nenhum momento e de que será mantido o caráter confidencial de todas as informações relacionadas à sua privacidade.

Comprometemo-nos a prestar-lhe informação atualizada durante o estudo, mesmo que isso possa afetar sua vontade de continuar participando da pesquisa.

Se o (a) senhor (a) se sentir prejudicado por ter participado desta pesquisa, o (a) senhor (a) poderá buscar indenização de acordo com as leis vigentes no Brasil.

______________________________	___________________________	_____________
Nome do sujeito de pesquisa	Assinatura do sujeito de pesquisa	Data

______________________________	___________________________	_____________
Nome da pesquisadora	Assinatura da pesquisadora	Data

APÊNDICE B – CARACTERIZAÇÃO SOCIODEMOGRÁFICA E CLÍNICA[1]

ID: _________

PRÉ-OPERATÓRIO

Data da entrevista: ________________ **Data nascimento:** ________________

Data internação: ________________

Sexo: 1. ( ) feminino 0. ( ) masculino

Presença de companheiro: 1. ( ) sim 0. ( ) não

Escolaridade (em anos COMPLETOS): _____________________

Situação profissional: 0.( ) ativo 1. ( ) inativo

Renda mensal familiar (em reais): _____________ nº de pessoas que dependem da renda: ____________

Diagnóstico principal: Coronariopatia 1. ( ) sim 0. ( ) não **Valvopatia** 1. ( ) sim 0. ( ) não

Doença da aorta 1. ( ) sim 0. ( ) não

Diagnósticos no prontuário médico: ____

____

Hábitos de vida:

Tabagismo atual: 1.( ) sim 2.( ) não Tempo: ___________ Número cigarros/dia: __________

Tabagismo pregresso: 1.( ) sim 2.( ) não Tempo de tbg: ___________ Número cigarros/dia: __________

Patologias associadas:

Peso= _______________ Altura= _________________

Dislipidemia 1.( ) sim 0.( ) não Hipotireoidismo 1.( ) sim 0.( ) não

Diabetes mellitus 1.( ) sim 0.( ) não Doença Pulmonar Obstrutiva Crônica 1.( ) sim 0.( ) não

Doenças neurológicos 1.( ) sim 0.( ) não Insuficiência renal aguda 1.( ) sim 0.( ) não

Insuficiência renal crônica 1.( ) sim 0.( ) não Hipertensão arterial sistêmica 1.( ) sim 0.( ) não

Insuficiência cardíaca 1.( ) sim 0.( ) não

Fibrilação atrial 1.( ) sim 0.( ) não

Outras patologias associadas 1.( ) sim 0.( ) não Qual (s): ______________________________

Medicamentos prescritos: ____

____

Uso de medicamentos controlados (com receita azul) em casa: 1. ( ) sim 0. ( ) não

Nome do medicamento: __________________________ **Faz uso há quanto tempo?** ___________

Frequência (vezes por dia): 1. ( ) 1 vez 2. ( ) 2 vezes 3. ( ) mais de duas vezes 4. ( ) esporadicamente -88. ( ) não se aplica

Cirurgia remarcada: 1. ( ) sim 2. ( ) não

Frequência: 1. ( ) 1 vez 2. ( ) 2 vezes 3.( ) mais de duas vezes

Motivo: ____________________________

Presença de dor precordial durante a internação: 1. ( ) sim 2. ( ) não

Número de cirurgia: 1. ( ) primeira cirurgia 2. ( ) reoperação Quantas vezes:_________________

[1] Apêndice-B: Instrumento de caracterização sociodemográfica e clínica dos participantes, nos períodos pré e pós-operatórios

INTRAOPERATÓRIO

Data da cirurgia: _______________

Cirurgia realizada: ____

PÓS-OPERATÓRIO MEDIATO – ENFERMARIA

Data da admissão PO: _________________ h

Data da alta da enfermaria: _____________________

Data do primeiro retorno no ambulatório: _____________________

ANEXOS

ANEXO A – Ofício de aprovação do Comitê de Ética em Pesquisa da Escola de Enfermagem de Ribeirão Preto

UNIVERSIDADE DE SÃO PAULO
ESCOLA DE ENFERMAGEM DE RIBEIRÃO PRETO

Centro Colaborador da OPAS/OMS para o Desenvolvimento da Pesquisa em Enfermagem

Avenida Bandeirantes, 3900 - Ribeirão Preto - São Paulo - Brasil - CEP 14040-902
Fone: 55 16 3315.3382 / 55 16 3315.3381 - Fax: 55 16 3315.0518
www.eerp.usp.br - eerp@usp.br

Ofício CEP-EERP/USP nº 110/2018, de 22/05/2018

Prezada Senhora,

Comunicamos que as alterações no projeto de pesquisa abaixo especificado foram analisadas e consideradas **aprovadas "ad referendum"** pelo Comitê de Ética em Pesquisa da Escola de Enfermagem de Ribeirão Preto da Universidade de São Paulo (CEP-EERP/USP) em 22 de maio de 2018.

Protocolo CAAE: 75120717.9.0000.5393

Projeto: Comparação dos sintomas de ansiedade, depressão e ansiedade cardíaca em pacientes no pré e pós-operatório de cirurgias cardíacas

Pesquisadores: Carina Aparecida Marosti Dessotte

Em atendimento à Resolução 466/12, deverá ser encaminhado ao CEP o relatório final da pesquisa e a publicação de seus resultados, para acompanhamento, bem como comunicada qualquer intercorrência ou a sua interrupção.

Atenciosamente,

Prof.ª Dra. Angelita Maria Stabile
Coordenadora do CEP-EERP/USP

Ilma. Sra.
Prof.ª Dra. Carina Aparecida Marosti Dessotte
Departamento de Enfermagem Geral e Especializada
Escola de Enfermagem de Ribeirão Preto - USP

ANEXO B – Questionário de Ansiedade Cardíaca[2]

ID: _____

Por favor, avalie cada item marcando a resposta que melhor corresponde ao que acontece com você:

	Nunca	Raramente	Às vezes	Frequentemente	Sempre
1. Presto atenção nas batidas do meu coração					
2. Evito esforço físico					
3. Meu coração acelerado me acorda à noite					
4. Dor ou desconforto no peito me acordam à noite					
5. Pego leve o máximo possível					
6. Evito fazer exercícios ou outras atividades físicas					
7. Evito atividades que acelerem o meu coração					
8. Mesmo que os exames estejam normais, eu continuo me preocupando com o meu coração					
9. Evito atividades que me façam suar					
10. Preocupa-me que os médicos não acreditem que meus sintomas sejam verdadeiros					
11. Quando tenho desconforto no peito ou meu coração está acelerado, preocupa-me que posso ter um ataque cardíaco					
12. Quando tenho desconforto no peito ou meu coração está acelerado, tenho dificuldade de me concentrar em qualquer outra coisa					
13. Quando tenho desconforto no peito ou meu coração está acelerado, fico com medo					
14. Quando tenho desconforto no peito ou meu coração está acelerado, gosto de ser examinado por um médico					

[2] SARDINHA, A.; NARDI, A.E.; ARAÚJO, C.G.S.; FERREIRA, M.C.; EIFERTT, G.H. Validação da versão Brasileira do Questionário de Ansiedade Cardíaca. **Arquivos Brasileiros de Cardiologia**, vol.101, n.6, p.554-561, 2013.

ANEXO C – Escala Hospitalar de Ansiedade e Depressão (HADS)[3]

ID: _________

Marque com um X a resposta que melhor corresponder a como você se sente agora.
Não é preciso ficar pensando muito em cada questão. Neste questionário as respostas espontâneas têm mais valor do que aquelas em que se pensa muito.
Marque apenas uma resposta para cada pergunta.

A Eu me sinto tenso ou contraído:
3 ( ) A maior parte do tempo
2 ( ) Boa parte do tempo
1 ( ) De vez em quando
0 ( ) Nunca

D Eu ainda sinto gosto pelas mesmas coisas de antes:
0 ( ) Sim, do mesmo jeito que antes
1 ( ) Não tanto quanto antes
2 ( ) Só um pouco
3 ( ) Já não sinto mais prazer em nada

A Eu sinto uma espécie de medo, como se alguma coisa ruim fosse acontecer:
3 ( ) Sim, e de um jeito muito forte
2 ( ) Sim, mas não tão forte
1 ( ) Um pouco, mas isso não me preocupa
0 ( ) Não sinto nada disso

D Dou risada e me divirto quando vejo coisas engraçadas:
0 ( ) Do mesmo jeito que antes
1 ( ) Atualmente um pouco menos
2 ( ) Atualmente bem menos
3 ( ) Não consigo mais

A Estou com a cabeça cheia de preocupações:
3 ( ) A maior parte do tempo
2 ( ) Boa parte do tempo
1 ( ) De vez em quando
0 ( ) Raramente

D Eu me sinto alegre:
3 ( ) Nunca
2 ( ) Poucas vezes
1 ( ) Muitas vezes
0 ( ) A maior parte do tempo

A Consigo ficar sentado à vontade e me sentir relaxado:
0 ( ) Sim, quase sempre
1 ( ) Muitas vezes
2 ( ) Poucas vezes
3 ( ) Nunca

---

[3] BOTEGA, N. J.; PONDÉ, M. P.; MEDEIROS, P.; LIMA, M. G.; GUERREIRO, C. A. M. Validação da escala hospitalar de ansiedade e depressão (HAD) em pacientes epiléticos ambulatoriais. **Jornal Brasileiro de Psiquiatria**, v. 47, n. 6, p. 285-289, jun.1998.

D Eu estou lento para pensar e fazer as coisas:
3 ( ) Quase sempre
2 ( ) Muitas vezes
1 ( ) De vez em quando
0 ( ) Nunca

A Eu tenho uma sensação ruim de medo, como um frio na barriga ou um aperto no estômago:
0 ( ) Nunca
1 ( ) De vez em quando
2 ( ) Muitas vezes
3 ( ) Quase sempre

D Eu perdi o interesse em cuidar da minha aparência:
3 ( ) Completamente
2 ( ) Não estou mais me cuidando como eu deveria
1 ( ) Talvez não tanto quanto antes
0 ( ) Me cuido do mesmo jeito que antes

A Eu me sinto inquieto, como se eu não pudesse ficar parado em lugar nenhum:
3 ( ) Sim, demais
2 ( ) Bastante
1 ( ) Um pouco
0 ( ) Não me sinto assim

D Fico esperando animado as coisas boas que estão por vir:
0 ( ) Do mesmo jeito que antes
1 ( ) Um pouco menos do que antes
2 ( ) Bem menos do que antes
3 ( ) Quase nunca

A De repente, tenho a sensação de entrar em pânico:
3 ( ) A quase todo momento
2 ( ) Várias vezes
1 ( ) De vez em quando
0 ( ) Não sinto isso

D Consigo sentir prazer quando assisto um bom programa de televisão, de rádio, ou quando leio alguma coisa:
0 ( ) Quase sempre
1 ( ) Várias vezes
2 ( ) Poucas vezes
3 ( ) Quase nunca
